# Risk factors for inadequate and excessive gestational weight gain in 25 low- and middle-income countries: An individual-level participant meta-analysis

**DOI:** 10.1371/journal.pmed.1004236

**Published:** 2023-07-24

**Authors:** Anne Marie Darling, Dongqing Wang, Nandita Perumal, Enju Liu, Molin Wang, Tahmeed Ahmed, Parul Christian, Kathryn G. Dewey, Gilberto Kac, Stephen H. Kennedy, Vishak Subramoney, Brittany Briggs, Wafaie W. Fawzi, members of the GWG Pooling Project Consortium

**Affiliations:** 1 Department of Global Health and Population, Harvard T.H. Chan School of Public Health, Harvard University, Boston, Massachusetts, United States of America; 2 Department of Global and Community Health, College of Health and Human Services, George Mason University, Fairfax, Virginia, United States of America; 3 Institutional Centers for Clinical and Translational Research, Boston Children’s Hospital, Boston, Massachusetts, United States of America; 4 Division of Gastroenterology, Hepatology and Nutrition, Boston Children’s Hospital, Harvard Medical School, Boston, Massachusetts, United States of America; 5 Department of Epidemiology, Harvard T.H. Chan School of Public Health, Harvard University, Boston, Massachusetts, United States of America; 6 Department of Biostatistics, Harvard T.H. Chan School of Public Health, Harvard University, Boston, Massachusetts, United States of America; 7 Nutrition & Clinical Services, International Centre for Diarrheal Disease Research, Bangladesh; 8 Department of International Health, Johns Hopkins Bloomberg School of Public Health, Baltimore, Maryland, United States of America; 9 Department of Nutrition, University of California, Davis, Davis, California, United States of America; 10 Nutritional Epidemiology Observatory, Josué de Castro Nutrition Institute, Federal University of Rio de Janeiro, Rio de Janeiro, Brazil; 11 Nuffield Department of Women’s & Reproductive Health, University of Oxford, Oxford, United Kingdom; 12 Certara Canada, Montreal, Quebec, Canada; 13 Certara USA, Inc. on behalf of the Bill & Melinda Gates Foundation, Seattle, Washington, United States of America; 14 Department of Nutrition, Harvard T.H. Chan School of Public Health, Harvard University, Boston, Massachusetts, United States of America

## Abstract

**Background:**

Many women experience suboptimal gestational weight gain (GWG) in low- and middle-income countries (LMICs), but our understanding of risk factors associated with GWG in these settings is limited. We investigated the relationships between demographic, anthropometric, lifestyle, and clinical factors and GWG in prospectively collected data from LMICs.

**Methods and findings:**

We conducted an individual participant-level meta-analysis of risk factors for GWG outcomes among 138,286 pregnant women with singleton pregnancies in 55 studies (27 randomized controlled trials and 28 prospective cohorts from 25 LMICs). Data sources were identified through PubMed, Embase, and Web of Science searches for articles published from January 2000 to March 2019. Titles and abstracts of articles identified in all databases were independently screened by 2 team members according to the following eligibility criteria: following inclusion criteria: (1) GWG data collection took place in an LMIC; (2) the study was a prospective cohort or randomized trial; (3) study participants were pregnant; and (4) the study was not conducted exclusively among human immunodeficiency virus (HIV)-infected women or women with other health conditions that could limit the generalizability of the results. The Institute of Medicine (IOM) body mass index (BMI)-specific guidelines were used to determine the adequacy of GWG, which we calculated as the ratio of the total observed weight gain over the mean recommended weight gain. Study outcomes included severely inadequate GWG (percent adequacy of GWG <70), inadequate GWG (percent adequacy of GWG <90, inclusive of severely inadequate), and excessive GWG (percent adequacy of GWG >125). Multivariable estimates from each study were pooled using fixed-effects meta-analysis. Study-specific regression models for each risk factor included all other demographic risk factors measured in a particular study as potential confounders, as well as BMI, maternal height, pre-pregnancy smoking, and chronic hypertension. Risk factors occurring during pregnancy were further adjusted for receipt of study intervention (if any) and 3-month calendar period. The INTERGROWTH-21st standard was used to define high and low GWG among normal weight women in a sensitivity analysis. The prevalence of inadequate GWG was 54%, while the prevalence of excessive weight gain was 22%. In multivariable models, factors that were associated with a higher risk of inadequate GWG included short maternal stature (<145 cm), tobacco smoking, and HIV infection. A mid-upper arm circumference (MUAC) of ≥28.1 cm was associated with the largest increase in risk for excessive GWG (risk ratio (RR) 3.02, 95% confidence interval (CI) [2.86, 3.19]). The estimated pooled difference in absolute risk between those with MUAC of ≥28.1 cm compared to those with a MUAC of 24 to 28.09 cm was 5.8% (95% CI 3.1% to 8.4%). Higher levels of education and age <20 years were also associated with an increased risk of excessive GWG. Results using the INTERGROWTH-21st standard among normal weight women were similar but attenuated compared to the results using the IOM guidelines among normal weight women. Limitations of the study’s methodology include differences in the availability of risk factors and potential confounders measured in each individual dataset; not all risk factors or potential confounders of interest were available across datasets and data on potential confounders collected across studies.

**Conclusions:**

Inadequate GWG is a significant public health concern in LMICs. We identified diverse nutritional, behavioral, and clinical risk factors for inadequate GWG, highlighting the need for integrated approaches to optimizing GWG in LMICs. The prevalence of excessive GWG suggests that attention to the emerging burden of excessive GWG in LMICs is also warranted.

## Abstract 2

Using individual participant data from more than 140,000 pregnant women across 25 low- and middle-income countries, Anne Marie Darling and colleagues explore the risk factors associated with both inadequate and excessive gestational weight gain.

## Author summary

**Why was this study done?:**

- Gestational weight gain (GWG) during pregnancy is a useful indicator for detecting potential maternal and infant health concerns.
- GWG below the recommended range, termed “inadequate,” has been found to be associated with higher risk of stillbirth, small for gestational age (SGA), and preterm birth.
- GWG above the recommended range, termed “excessive,” has been found to be associated with higher risk of large for gestational age (LGA), macrosomia, cesarean delivery, postpartum weight retention, and child overweight.
- Identifying modifiable risk factors for inadequate and excessive GWG is necessary for the development of evidence-based policies and programs that promote GWG within recommended ranges, but the evidence base for these risk factors is limited in in low- and middle-income countries (LMICs).

**What did the researchers do and find?:**

- We pooled data on pregnancy weight gain and potential risk factors from 55 prospective cohort and randomized clinical trials contributed by members of the GWG Pooling Project consortium to create a large dataset of 138,286 pregnant women from 25 countries.
- The pooled prevalence of severely inadequate, inadequate (inclusive of severely inadequate), and excess GWG was 34.2%, 53.9%, and 22.0%, respectively.
- Anthropometric factors such as body mass index (BMI), mid-upper arm circumference (MUAC), and height were strongly associated with inadequate, severely inadequate, and excessive weight gain.
- Smoking and HIV infection were associated with a higher risk of inadequate and severely inadequate weight gain, while higher levels of education were associated with a lower risk. Higher levels of education were also associated with a higher risk of excessive weight gain.

**What do these findings mean?:**

- Inadequate GWG is a major public health concern in LMICs, and several demographic, nutritional, substance use, and clinical factors may perpetuate its occurrence.
- Comprehensive interventions to improve maternal health and nutrition status and promote healthy behaviors are needed.
- The extent of excessive GWG and its determinants is also a public health concern and warrants additional research.

## Introduction

Gestational weight gain (GWG) is defined in terms of the amount of weight gained between conception and just before birth. Adequacy of GWG is commonly determined in relation to body mass index (BMI) category-specific recommended ranges established by the Institute of Medicine (IOM) [1]. GWG during pregnancy is a useful indicator for detecting potential maternal and infant health concerns. GWG below the recommended range, termed “inadequate,” has been found to be associated with higher risk of stillbirth [2], small for gestational age (SGA) [3,4], and preterm birth [3]. GWG above the recommended range, termed “excessive,” has been found to be associated with higher risk of large for gestational age (LGA) [3,4,5], macrosomia [3,4,5], cesarean delivery [3,4,5], postpartum weight retention [5], and child overweight. Furthermore, through their detrimental impact on offspring nutritional status, inadequate and excessive GWG can contribute to intergenerational cycles of undernutrition and obesity [6,7].

Identifying modifiable risk factors for inadequate and excessive GWG is necessary for the development of evidence-based policies and programs that promote GWG within recommended ranges. In previous studies, predominantly conducted in high-income settings, several individual risk factors for inadequate GWG have been identified, most notably both lower and higher BMI [8,9,10]. Other identified risk factors include younger maternal age [8,11,12], short stature [13], multiparity [8,13–15], single motherhood [12], smoking [9], gestational diabetes [16], and reduced food intake during pregnancy [9]. Repeated observed associations between measures of low socioeconomic status (SES) and inadequate weight gain [9,13,14,17] also suggest that, beyond individual risk factors, broader social inequalities may play a role. Higher BMI [9,10,12,15,16,18–21] is a well-documented individual risk factor for excessive GWG, again primarily from research conducted in high-income settings. Younger maternal age [11,12,18], tall stature [11], nulliparity [19], single motherhood [12], alcohol consumption [22], and a decline in physical activity during pregnancy [8,20,21] have also been associated with excessive GWG. Similar to inadequate GWG, observed associations between measures of low SES and excessive GWG suggest that social inequalities may contribute.

Though most research to date on risk factors for inadequate and excessive GWG has been conducted in high-income countries (HICs), the topic is particularly salient in low- and middle-income countries (LMICs). Population-based data on GWG in LMICs are largely unavailable, but a recent modeling analysis using nationally representative data from the Demographic and Health Surveys of LMICs estimated that mean GWG in 2015 was lower than the minimum recommended GWG for women with normal weight in most regions [23] and that estimated mean GWG in sub-Saharan Africa and North Africa and the Middle East was below 60% of the minimum recommendation. Inadequate GWG was also associated with adverse birth outcomes such as preterm birth, SGA, and low birthweight prevalent in resource-limited settings [24,25]. At the same time, the proportion of individuals living with overweight and obesity is increasing in LMICs [26], which may lead to a corresponding increase in the prevalence of excessive GWG. Due to the double burden of malnutrition, women entering pregnancy in LMICs are vulnerable to a range of nutritional concerns, including undernutrition, micronutrient deficiencies, nutrition-related chronic disease, overweight, obesity, or combinations of these [27]. More research is therefore needed to determine which risk factors for inadequate and excessive GWG are most relevant to the design of effective public health interventions promoting healthy pregnancy GWG in these settings.

In this study, we pooled individual-level data from randomized controlled trials and prospective cohort studies previously conducted in LMICs with the aim of characterizing the associations between selected demographic, anthropometric, lifestyle, and clinical factors and inadequate and excessive GWG.

## Methods

### Ethics statement

The Harvard T.H. Chan School of Public Health Institutional Review Board determined this secondary analysis of existing data was not human participants research because all data had been deidentified prior to receipt. Informed consent was therefore not considered applicable.

### Systematic literature review

In February and March 2019, members of the analytic team conducted a systematic search using PubMed, Embase, and Web of Science databases to identify prospective longitudinal studies that performed multiple weight measurements during pregnancy in countries classified as being low- or middle-income by the World Bank in 2019. Search terms included MeSH headings and keywords related to pregnancy, weight gain, randomized trials or prospective cohort studies, and names of individual LMICs (Table A in S1 Appendix). We imposed a publication date restriction of the year 2000 and later to capture relatively recent studies for the purpose of generalizability. Titles and abstracts of articles identified in all databases were independently screened by 2 team members. Abstracts were screened to ensure the study included repeated weight measures during pregnancy in an LMIC. Full-text reviews were performed on all selected abstracts independently by 2 team members.

The final selection of potential datasets was based on the following inclusion criteria: (1) GWG data collection took place in an LMIC; (2) the study was a prospective cohort or randomized trial; (3) study participants were pregnant; and (4) the study was not conducted exclusively among human immunodeficiency virus (HIV)-infected women or women with other health conditions that could limit the generalizability of the results. This study is reported as per the Preferred Reporting Items for Systematic Reviews and Meta-Analyses (PRISMA) guideline (S1 PRISMA Checklist). It does not have a preregistered protocol.

### Dataset eligibility, contribution, and harmonization

**Fig 1:**
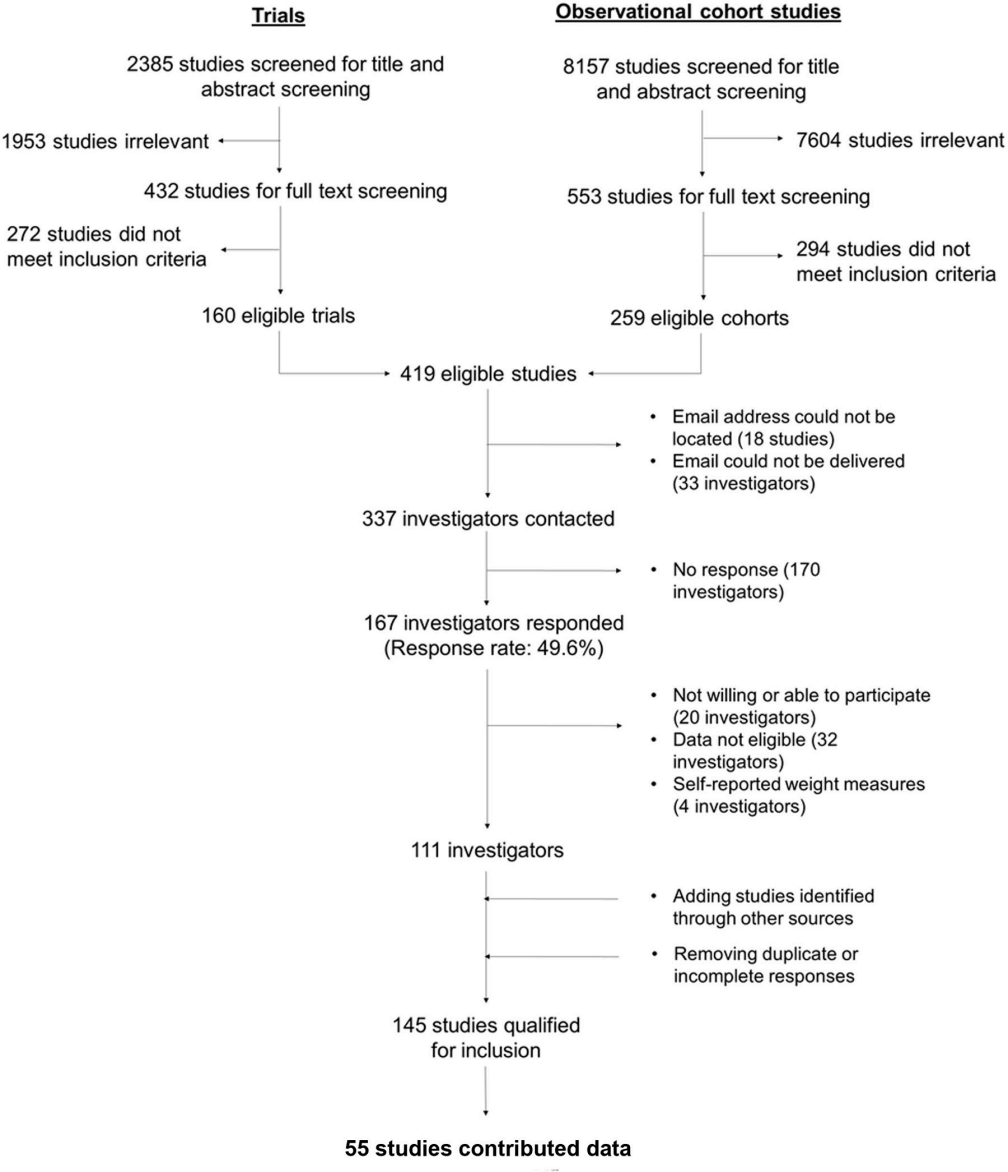
PRISMA flow chart.

Principal investigators of the identified studies were e-mailed a questionnaire about their potentially eligible datasets and any others they had collected. Those who confirmed the eligibility of their data were invited to collaborate and contribute to the pooled analysis. Among the 337 investigators contacted, 50% responded to the survey, of whom 145 were eligible for the pooled analysis and invited to contribute data (Fig 1). Two investigators additionally contributed data from unpublished studies that met the eligibility criteria. The analysis was further restricted to participants with singleton pregnancies and measured heights.

### Risk factors for inadequate and excessive GWG

Potential risk factors for GWG were selected based on findings from the literature [8–22] and examined in pooled analyses if they had been collected in 3 or more studies. Within these studies, risk factor values were categorized as missing if unknown. Demographic variables included age (<20, 20 to 29, ≥30 years), woman’s education (0 to 7, 8 to 11, ≥12 years based on data distribution), partner’s education (0 to 7, 8 to 11, ≥12 years based on data distribution), woman’s employment outside the home (none, informal/agricultural, formal), partner’s employment outside the home (none, informal/agricultural, formal), married or cohabiting (yes/no), and parity (0, 1, 2, 3, ≥4 previous live births based on data distribution). We classified raw data on employment according to our best judgment about whether they most closely aligned with agricultural, informal, or formal sectors based on published operational definitions [28].

Anthropometric variables included women’s first-trimester BMI, mid-upper arm circumference (MUAC), and height. BMI, derived from measured or imputed first-trimester weight, was categorized into underweight (<18.5 kg/m^2^), normal weight (18.5 to 24.9 kg/m^2^), overweight (25.0 to 29.9 kg/m2), or obese (≥30 kg/m2). For women less than 20 years old, BMI was classified according to the WHO adolescent growth reference [29], in which a BMI for age of <−2 standard deviations (SD) was defined as underweight, −2 SD to <1 SD was defined as normal weight, 1 SD to <2 SD was defined as overweight, and ≥2 SD was defined as obese. If participants’ first-trimester weight was not available, we imputed this value using the method described below. Participants’ first MUAC measurement was categorized based on published cutoff points. Cutoff points of <24 cm [30] and ≥28.1 [31] were categorized as underweight and overweight/obesity, respectively. Height was categorized as <145, 145 to <150, 150 to <155, and ≥155 [32].

Substance use risk factors included smoking and alcohol. Smoking status was categorized as use or nonuse during the following 4 time periods: pre-pregnancy, first trimester, second trimester, and third trimester. Alcohol consumption was categorized into use or nonuse during the first trimester, second trimester, or third trimester.

Clinical variables included the presence or absence of chronic hypertension, HIV infection, malaria at ≤36 weeks of gestation, nausea or vomiting, diarrhea, and anemia. Chronic hypertension was defined as systolic blood pressure of ≥140 mm Hg or diastolic blood pressure of ≥90 mm Hg before 20 weeks of gestation [33]. Malaria at or before 36 weeks was ascertained through a thick blood film examination using microscopy, polymerase chain reaction, or rapid diagnostic test on peripheral blood. Report of acute or chronic diarrhea at any time during pregnancy and nausea or vomiting of any severity at any time during pregnancy were assessed dichotomously. Anemia was defined as at least one hemoglobin measurement <11.0 g/dL in accordance with the WHO definition [34].

### Outcome definitions

Assessment of GWG using the IOM criteria requires a weight measurement during pre-pregnancy or the first trimester, which was often unavailable in the datasets. Therefore, in these cases, we imputed weight at 9 weeks of gestation for the 33% of participants for whom a first-trimester weight was not available. We chose the 9-week time point to balance the degree of extrapolation (i.e., imputing values further away from the center of the available data for studies with no first-trimester weight). Gestational age was ascertained through ultrasound or date of last menstrual period. We performed the imputation by deriving subject-specific slopes and intercepts from a mixed-effects restricted cubic spline model regressing weight on gestational age with 3 knots based on the pooled database stratified by geographic region. We previously developed and validated this imputation approach and compared it with alternative strategies [35]. Validation suggested that the accuracy of this imputation approach is high since the mean absolute error was 1 to 2 kg. Using this method, imputed weights approximated measured weights in 2 pregnancy cohorts with a mean absolute error of 1.60 kg and 1.99 kg. This imputation method will, therefore, lead to only a minimal amount of outcome misclassification since our GWG outcome is categorical (i.e., this degree of error is unlikely to result in substantial misplacement of a large number of participants in weight categories).

We calculated total GWG as the difference in kilograms between the last available weight measure and imputed or observed first-trimester weight. We used this measurement to calculate the percent adequacy of GWG based on IOM guidelines [1].

To ensure that GWG was independent of gestational duration, we undertook a 2-step process. First, we estimated the amount of weight a woman was expected to gain up to the last observed weight measurement according to the IOM 2009 recommendations using the following formula:

Expected GWG = (Expected first-trimester weight gain / 13.86) * (13.86 –gestational age at first observed or imputed weight measurement) + [(gestational age at the last weight measurement– 13weeks, 6 days (equivalent to 13.86 weeks)) × mean recommended rate of GWG for the second and third trimester by BMI category based on the IOM guidelines]. The expected GWG for the first trimester was defined as 2 kg for women with underweight and women with normal weight, 1 kg for women with overweight, and 0.5 kg for women with obesity. Mean recommended rates of GWG for the second and third trimesters were 0.51, 0.42, 0.28, and 0.22 kg per week for women with underweight, normal weight, overweight, and obesity, respectively. We then calculated the adequacy by dividing the actual GWG by the expected GWG at the last observed weight measurement (i.e., the amount of weight a woman was supposed to gain/week), multiplied by 100. We further classified the adequacy of GWG as severely inadequate (<70%), inadequate (<90% (inclusive of severely inadequate)), adequate (90 to 125%), or excessive (>125%). The cutoffs of <90% and >125% were chosen because they correspond to the lower and upper limits of the IOM recommended weekly GWG range, which represent approximately 90% and 125% of the recommended mean rate of GWG [36]. Because these IOM-based categorizations, which were developed based on research from HICs, did not fully capture the severity of inadequate GWG in these data from LMICs, we created an additional category (<70%) to reflect this.

### Statistical analyses

We conducted 2-stage pooled analyses for risk factors of interest that were measured in at least 3 studies. In a few cases, we excluded studies if the risk factor had an extremely high number of missing values. These analyses involved first estimating study-specific regression coefficients using modified Poisson regression with robust variance estimates and pooling all regression coefficients in a meta-analysis. Outcomes included severely inadequate GWG, inadequate GWG (inclusive of severely inadequate GWG), and excessive GWG, which were modeled as dichotomous variables such that participants in all other GWG categories were included in the reference group. We chose to include participants in all other GWG categories rather than solely the adequate category since this category represented a minority of participants in most studies. Study-specific regression models included all other demographic risk factors noted above that were measured in a particular study as potential confounders, as well as BMI, height, pre-pregnancy smoking, and chronic hypertension. Because height and BMI are included in the same model, the coefficient for BMI is interpreted as overall adiposity, while the coefficient for height is interpreted as a surrogate of childhood and adolescent nutritional status [37]. Due to concerns regarding unclear temporal relationships, risk factors occurring during pregnancy were not adjusted for in any models with the exception of receipt of study intervention (if any). These models were also adjusted for the 3-month calendar period when the participant reached 9 weeks of gestation to account for seasonal weight gain patterns within studies. In addition, models containing MUAC were not adjusted for pre-pregnancy BMI, given that these measures are highly correlated (r = 0.75 in these data). We used a missing indicator approach to account for confounder missingness [38].

To minimize nonconvergence of regression models due to 0 cell counts, all analyses were limited to studies in which at least 3 or more participants experienced the outcomes of interest. When modified Poisson models did not produce robust confidence intervals due to model instability, Wald confidence intervals were calculated instead. Regression parameters were then pooled in a fixed-effects meta-analysis to obtain pooled risk ratios (RRs) and 95% confidence intervals (CIs). The I^2^ statistic assessed the percentage of variance attributable to the heterogeneity of the included studies. The 2-stage analyses are considered the primary results since they enable pooling with maximal adjustment for covariates in each study.

To evaluate the robustness of the associations estimated through the 2-stage method, in which each study was adjusted for a different set of confounders, we also conducted 1-stage analyses for all risk factors of interest among studies that had collected the following minimal set of participant characteristics: maternal age, education, height, and BMI. These covariates were chosen because they had been reported across the largest number of studies. Studies that did not contain all these variables were excluded. The 1-stage analyses consisted only of modified Poisson regression models and included data from all studies simultaneously.

Each risk factor was examined in a separate model adjusted for the minimal set of covariates listed above. A covariate with 55 levels representing the 55 individual studies was also included in the 1-stage models. Although a risk factor had to be measured in at least 3 studies to be included in the 2-stage analysis, this criterion was relaxed to 2 or more studies in the 1-stage analyses since not all studies measured the minimal set of potential confounders.

We conducted some additional sensitivity analyses. Where 2-stage analyses were unfeasible due to prohibitively small subsamples in some studies, we used 1-stage models. We examined effect measure modification of the associations between risk factors and GWG outcomes by first-trimester BMI category. We also examined the association between risk factors and high and low weight gain as defined by the INTERGROWTH-21st maternal weight gain standards [39]. Unlike the IOM guidelines, these standards are based on WHO recommendations for the production of international, prescriptive standards; weight gain patterns were observed in optimally healthy pregnant women from 8 geographically diverse populations from HICs and LMICs. The INTERGROWTH-21st standards may therefore be more generalizable to women in LMIC settings than the IOM guidelines, but we did not use them in the primary analysis since their applicability to pregnant women living with underweight or overweight/obesity is unknown. For this secondary analysis, which was limited to women with normal weight based on early pregnancy BMI, we used SD-based cutoff points as is common for anthropometric measures since no prescribed cutoff points are available. Very low weight gain was defined as a maternal weight gain z-score of <−2, low weight gain as a z-score of <−1 and high weight gain as a z-score ≥1. Categories were necessarily asymmetrical due to the small number of participants with a z-score above 2. Low and high weight gain were modeled as dichotomous outcomes in this analysis, with all participants gaining above and below these thresholds, respectively, included in the reference category. We also repeated the main 1-stage analysis while restricting to participants with measured weight values during the third trimester to ascertain associations with total GWG adequacy. In addition, we repeated the main 1-stage analysis using the lower limits of the IOM-recommended mean rate of weight gain in the second and third trimester rather than the mean itself to define expected weight gain. These values were 0.44 kg/week for women of underweight, 0.35 kg/week for women of normal weight, 0.23 kg/week for women with overweight, and 0.17 kg/week women with obesity. To ensure our results were robust to the use of lower BMI cutoffs for Asian participants, we repeated the primary 2-stage analyses using a cutoff of >23 kg/m^2^ [40] to define overweight for all participants from Asian countries. We repeated the primary 2-stage analysis limiting the reference category for each outcome to those with adequate GWG. Lastly, we conducted a 1-stage analysis limited to those in observational studies or who did not receive interventions in clinical trials, since these interventions could theoretically modify the association between the risk factors of interest and GWG. All statistical analyses were conducted in SAS 9.4 and Stata 14.

## Results

We included 55 studies in the analysis for a total sample size of 148,130 pregnant women. Twenty-seven of these studies were randomized trials, and 28 were prospective cohort studies (Table 1). Interventions provided in the trials are shown in Table B in S1 Appendix. The locations of these studies included 25 countries in Asia, Latin America, the Middle East, and sub-Saharan Africa. The median age of participants was 25 (interquartile range (IQR): 21, 30). The study prevalence of early pregnancy underweight was 20% (29,023/148,130), and the prevalence of early pregnancy overweight/obesity was 18% (27,007/148,130). Approximately four-fifths (79% (117,502/148,430)) of participants’ last weight values were measured in the third trimester. We excluded 9,844 participants without weight measurements after 13.86 weeks of gestation, since their GWG adequacy ratio could not be calculated. Therefore, the sample size for analytic purposes was 138,286.

**Table 1:** Characteristics of studies included in pooled analyses (*n =* 148,310).

Study acronym	Author, publication year	Country	Study type	Sample size	Age range	Mean (SD) BMI	Mean (SD) Height	Median (IQR) number of study visits	Median GA at last measured weight
LCSS	Espo 2002 [41]	Malawi	Cohort	598	13–49	20.1 (2)	155.2 (5.5)	2 (2, 2)	36.0 (32.9, 38.6)
NNIPS-3	Christian 2003 [42]	Nepal	Trial	2,960	10–45	19 (1.9)	150.2 (5.6)	2 (2, 2)	33.0 (31.3, 34.9)
EU-MMN	Ramakrishnan 2003 [43]	Mexico	Trial	457	10–39	24.2 (4.0)	148.9 (4.9)	2 (1,3)	29.3 (25.1, 32.1)
USP-MatStress	Rondó 2003 [44]	Brazil	Cohort	926	13–42	23.3 (3.9)	158.4 (6.1)	3 (3, 3)	34.0 (32.3, 35.7)
UZ-MatNutri	Friis 2004 [45]	Zimbabwe	Trial	425	15–45	22.9 (3.3)	161.6 (5.4)	1 (1, 1)	26.0 (24.9, 27.0)
Mira-Janakpur	Osrin 2005 [46]	Nepal	Trial	1,132	13–50	21 (3)	151.1 (5.4)	1 (1, 2)	19.4 (12.6, 36.6)
PNS	Fawzi 2007 [47]	Tanzania	Trial	7,577	14–46	23.3 (3.8)	155.5 (6)	5 (4, 5)	36.9 (34.0,38.9)
AKU-FatGDM	Iqbal 2007 [48]	Pakistan	Cohort	612	17–42	23.1 (4.0)	159.0 (5.5)	2 (2, 2)	29.0 (27.0, 31.0)
ICDDR-MINIMat	Tofail 2008 [49]	Bangladesh	Trial	3,560	14–50	20.1 (2.7)	149.8 (5.4)	4 (4, 4)	31.4 (30.6, 32.6)
MISAME-1	Roberfroid 2008 [50]	Burkina Faso	Trial	1,158	14–48	20.3 (2.2)	162.2 (5.9)	3 (2, 3)	35.0 (30.4, 37.3)
XJU-RuralChina	Zeng 2008 [51]	China	Trial	4,578	15–43	20.3 (2.1)	158.8 (5.2)	3 (2, 3)	32.1 (29.6, 32.6)
AKU-MMN	Bhutta 2009 [52]	Pakistan	Trial	1,560	14–45	21.2 (3.7)	152.9 (5.9)	11 (9, 12)	36.4 (32.8, 38.9)
Arg-GWG Curves	Calvo 2009 [53]	Argentina	Cohort	1,090	19–46	23.6 (4.8)	159.7 (6.7)	7 (6, 8)	37.0 (36.0, 38.0)
MISAME-2	Huybregts 2009 [54]	Burkina Faso	Trial	1,186	14–46	20.4 (2.1)	162.6 (5.9)	3 (2, 4)	35.3 (32.4, 37.3)
FU-GWG	Rodrigues 2010 [55]	Brazil	Cohort	176	18–40	23.7 (4.6)	159.4 (6.2)	3 (2, 4)	27.8 (22.0, 36.1)
UMan-MatHealth	Ayoola 2012 [56]	Nigeria	Cohort	351	15–44	23.5 (4.2)	160 (5.8)	5 (4, 6)	37.0 (34.0, 38.9)
MRCG@LSHTM-ENID	Moore 2012 [57]	The Gambia	Trial	836	17–48	21.2 (3.5)	161.9 (5.9)	3 (3, 3)	30.0 (29.7, 30.4)
USM-PregCohort	Loy 2014 [58]	Malaysia	Cohort	153	19–41	22.6 (4.1)	155.3 (5.6)	3 (3, 3)	39.4 (38.6, 40.1)
JiVitA3	West 2014 [59]	Bangladesh	Trial	24,059	10–47	19.2 (2.3)	149.7 (5.2)	2 (2, 2)	32.1 (31.9, 32.7)
ILINS-DYAD-G	Adu-Afarwuah 2015 [60]	Ghana	Trial	1,190	18–45	24 (4.4)	158.8 (5.7)	3 (2, 3)	36.1 (36.0, 36.7)
ILINS-DYAD-M	Ashorn 2015 [61]	Malawi	Trial	1,362	14–48	21.5 (2.7)	156.1 (5.7)	3 (3, 3)	36.1 (35.3, 36.7)
MAL1	Etheredge 2015 [62]	Tanzania	Trial	1,402	18–39	23.8 (4.5)	156.2 (6)	5 (3, 6)	35.7 (29.6, 38.1)
JHU-MothersGift	Tielsch 2015 [63]	Nepal	Trial	3,246	13–44	20.8 (2.8)	151.6 (5.6)	5 (4, 7)	36.7 (34.3, 38.3)
SPAZ-IPTp	Unger 2015 [64]	Papua New Guinea	Trial	1,983	15–45	21.2 (2.7)	154.3 (5.9)	3 (1, 3)	28.4 (23.9, 32.4)
FU-LEPTINGWG	Franco-Sena 2016 [65]	Brazil	Cohort	275	20–40	24.5 (4.6)	159.6 (6.3)	4 (3, 4)	37.0 (31.0, 38.9)
AKU-VITD	Khan 2016 [66]	Pakistan	Trial	545	16–40	22.5 (3.7)	154.5 (5.5)	4 (3, 5)	35.0 (32.4, 36.7)
UC-RDNS	Matias 2016 [67]	Bangladesh	Trial	3,819	14–50	19.8 (2.6)	150.5 (5.4)	2 (1, 2)	35.6 (21.3, 36.0)
MAL2	Darling 2017 [68]	Tanzania	Trial	2,128	18–45	23.2 (4.4)	154.6 (6.1)	6 (4, 8)	33.9 (26.1, 37.3)
XJU-Tibet	Kang 2017 [69]	China	Trial	1,039	17–42	20.1 (2.3)	160.9 (6.2)	3 (3, 3)	36.4 (32.8, 38.9)
MAHE-SCFPPP	Ramachandra 2017 [70]	India	Cohort	70	22–36	21.9 (3.5)	156.7 (5.1)	3 (3, 3)	32.0 (32.0, 32.0)
INPer-FICA	Sámano 2017 [71]	Mexico	Cohort	168	12–17	21.4 (3.4)	155.4 (3.7)	1 (1, 1)	38.9 (38.0, 39.9)
SHU-BMIGWG	Soltani 2017 [72]	Indonesia	Cohort	563	15–47	21.3 (3.6)	153.2 (5.6)	3 (2, 3)	34.0 (29.0,37.1)
NWU-PreNAPS	Widen 2017 [73]	Uganda	Cohort	240	18–39	22 (2.8)	163 (6)	5 (4, 6)	36.9 (35.1, 38.6)
UHAS-AHPI	Yeboah 2017 [74]	Ghana	Cohort	290	15–46	25.4 (4.3)	158.5 (4.9)	2 (2, 2)	24.0 (24.0, 24.0)
IRD-RECIPAL	Accrombessi 2018 [75]	Benin	Cohort	258	18–40	22.8 (4.1)	158.4 (6.1)	7 (6, 7)	37.7 (35.3, 38.7)
MDIG	Roth 2018 [76]	Bangladesh	Trial	1,283	18–40	22.1 (3.6)	151 (5.4)	3 (2, 3)	38.0 (30.3, 39.4)
INPer-REDES	Sámano 2018 [77]	Mexico	Cohort	335	12–18	21.3 (3)	155.6 (5.2)	1 (1, 1)	38.9 (37.9, 39.7)
UCL-LBWSAT	Saville 2018 [78]	Nepal	Trial	2,8	12–42	19.4 (2.1)	150.4 (5.5)	1 (1, 1)	23.4 (18.1, 27.7)
IMIP-GestDM	do Nascimento 2019 [79]	Brazil	Cohort	518	14–45	25.3 (4.4)	161.4 (6.8)	2 (2, 2)	30.0 (29.0, 32.0)
LAIS	Hallamaa 2019 [80]	Malawi	Trial	1,307	15–49	20.7 (2.1)	155.1 (5.5)	4 (4, 5)	35.9 (34.1, 37.7)
WomenFirst	Hambidge 2019 [81]	Guatemala, India, and Pakistan	Trial	1,985	16–37	21.9 (4.4)	149.8 (6.4)	7 (2, 9)	35.3 (32.9, 37.1)
SMRU	Hashmi 2019 [82]	Thailand^1^	Cohort	26,138	13–50	21 (3)	151.1 (5.4)	13 (7, 21)	19.4 (12.6, 36.6)
TU-Aflatoxin	Lauer 2019 [83]	Uganda	Cohort	246	18–45	23.3 (3.5)	158.5 (5.9)	2 (2, 2)	37.6 (37.0, 38.3)
ROSE	Isanaka 2019 [84]	Niger	Trial	2,182	14–51	21.4 (2.8)	157.4 (6.4)	3 (2. 3)	34.2 (30.0, 37.6)
SBUMS-GDM	Tehrani 2019 [85]	Iran	Trial	26,199	18–47	25.6 (4.7)	159.7 (5.8)	2 (2, 2)	38.1 (37.3, 39.0)
NWU-PMPEN	Widen 2019 [86]	Kenya	Cohort	209	18–41	23.5 (6.1)	160.8 (10)	2 (2, 2)	33.0 (31.1, 34.0)
HUST-TMCHC	Zhong 2019 [87]	China	Cohort	7,329	17–45	20.8 (2.7)	160.5 (4.9)	9 (5, 12)	39.5 (38.6, 40.4)
MINA-Brazil	Cardoso 2020 [88]	Brazil	Cohort	1,327	13–45	24.1 (4.4)	156.9 (6.1)	7 (5, 8)	37.7 (35.9, 39.0)
INPer-GDM	Samano 2020^2^	Mexico	Cohort	215	13–44	24.9 (5.4)	156.3 (5.6)	6 (5, 7)	35.6 (34.1, 37.1)
INPer-NeuroObesity	Samano 2020^2^	Mexico	Cohort	309	18–43	27.1 (5.2)	157.5 (5.9)	3 (3, 3)	29.4 (27.9, 31.0)
INPer-Poli	Samano 2020^2^	Mexico	Cohort	140	13–20	22.1 (3.1)	154.5 (5.1)	5 (2, 8)	38.9 (37.7, 39.6)
St-Johns	Dwarkanath 2020^2^	India	Cohort	2,001	16–41	21.8 (3.7)	155.3 (5.9)	3 (1, 3)	33.0 (15.7, 34.3)
IMIP-BRAMAG	de Araújo 2020 [89]	Brazil	Trial	928	18–41	25.7 (5)	161.7 (6.3)	3 (2, 3)	30.0 (26.0, 34.0)
HERO-G	Moore 2020 [90]	The Gambia	Cohort	249	18–45	21.6 (3.8)	162.5 (5.3)	4 (4, 5)	35.7 (35.4, 36.0)
INPer-CAR	Samano 2021 [91]	Mexico	Cohort	408	12–22	21.5 (3.6)	156.1 (5.6)	1 (1, 1)	38.9 (37.9, 39.9)

GA, gestational age; IQR, interquartile range; SD, standard deviation.

^1^Although data were collected in Thailand, the study population was composed of 99% Karen and Burmese women from Myanmar.

^2^These data were contributed by consortium members who had been contacted based on published datasets and determined that these unpublished data additionally met the eligibility criteria

**Fig 2:**
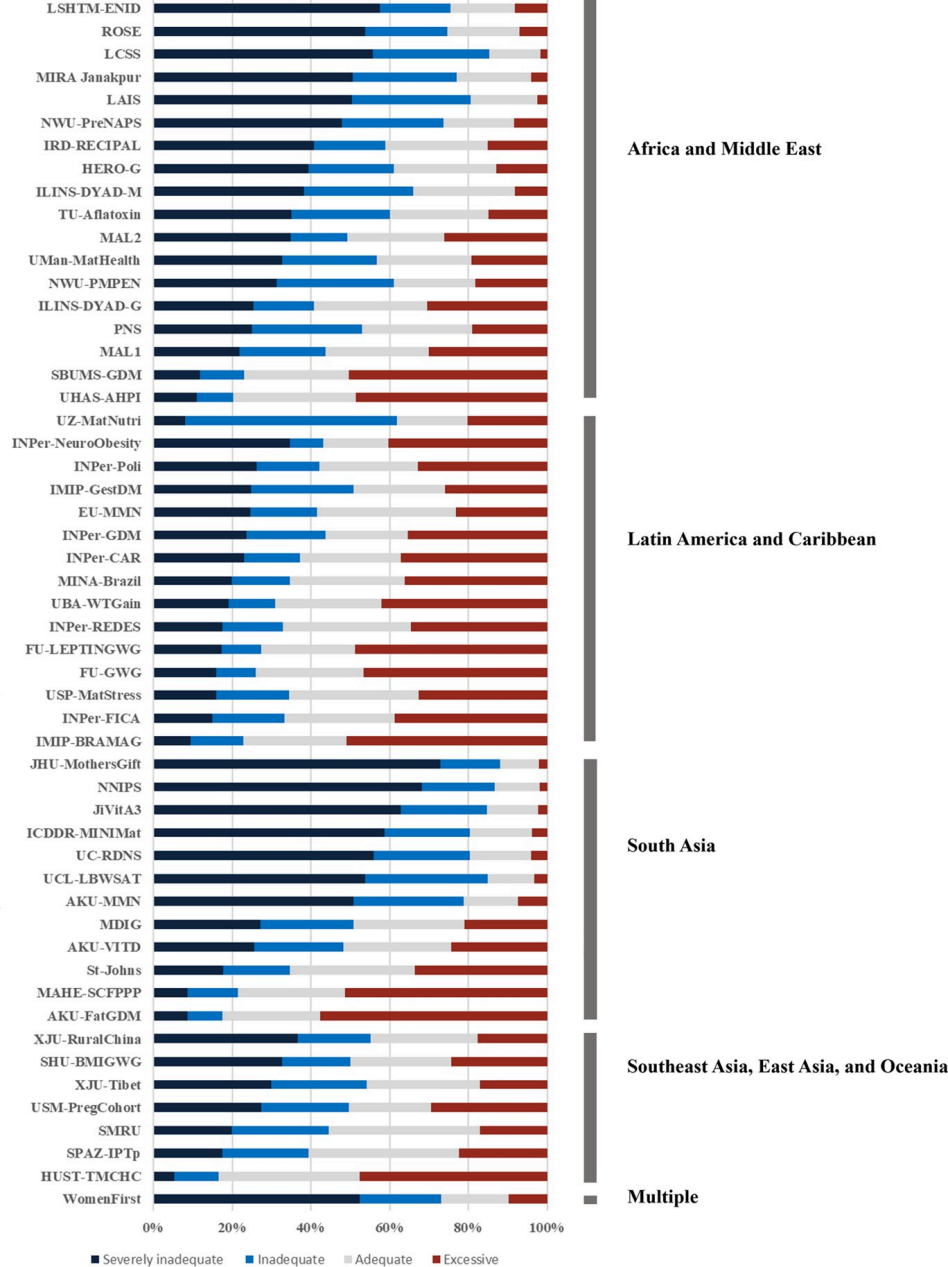
Distribution of GWG categories by study and geographic region (severely inadequate = <70% of IOM recommendations; inadequate = <90%; adequate– 90%–125%; excessive = >125%).

The proportion of participants experiencing inadequate or severely inadequate GWG ranged from 16% (1,207/7,325) in a study from China to 88% (2,801/3,182) in a study from Nepal, and the proportion of participants experiencing excessive GWG ranged from 2% (10/597) in a study from Malawi to 58% in a study from Pakistan. Fig 2 shows the distribution of GWG categories across studies by geographic region. The pooled prevalence of severely inadequate, inadequate (inclusive of severely inadequate), and excess GWG was 34.2% (47,302/138,286), 53.9% (74,524/138,286), and 22.0% (30,368/138,286), respectively. When restricting to data sources from middle-income countries, these proportions were 30.8% (33,434/108,573), 49.4% (53,655/108,573), and 25.1% (27,202/108,573). When restricting to data sources from low-income countries, they were 46.7% (13,868/29,713), 70.2% (20,869/29,713), and 10.7% (3,166/29,713). Table C in S1 Appendix provides the frequencies and percentages of all examined risk factors across studies.

### Demographic risk factors

Of the demographic factors we examined, women’s education and their partner’s education showed some of the largest associations with severely inadequate GWG (Table 2) and inadequate GWG (Table 3) in the 2-stage analysis. The risk of severely inadequate GWG was reduced among women with ≥12 years of education (RR 0.82, 95% CI [0.78, 0.86]) and those whose partners had ≥12 years of education (RR 0.85, 95% CI [0.79, 0.90]) compared to those with 0 to 7 years. These associations, albeit somewhat attenuated, were also observed in the 1-stage analysis (Figures A1-B2 in S1 Appendix). At the same time, women with ≥12 years of education or whose partners had ≥12 years of education had a higher risk of excessive GWG (RR 1.22, 95% CI [1.14, 1.31] and RR 1.34 95% CI [1.45, 1.57], respectively) (Table 4). However, associations were not observed in the 1-stage analysis (Figures C1-C2 in S1 Appendix).

**Table 2:** Two-stage pooled multivariable^1^ associations between participant characteristics and severely inadequate weight gain (*n =* 138,286)^2^.

	Number of participants	Number of studies	Crude RR (95% CI)	I^2^ (%)	Multivariable RR^1^	I^2^ (%)
Characteristic						
Woman’s age (years)	134,880	53				
<20			0.99 (0.97, 1.00)	0.0	0.97 (0.96, 0.99)	0.0
20–24			1.00 (Ref)		1.00 (Ref)	
25–29			0.99 (0.98, 1.01)	91.5	1.00 (0.98, 1.02)	90.4
30–34			0.99 (0.97, 1.02)	54.8	1.01 (0.99, 1.04)	29.0
≥35			1.12 (1.09, 1.15)	84.6	1.08 (1.05, 1.12)	49.5
Women’s educational level (years)	81,489	47				
0–7			1.00 (Ref)		1.00 (Ref)	
8–11			0.89 (0.88, 0.91)	99.2	0.94 (0.92, 0.95)	99.0
≥12			0.75 (0.72, 0.78)	64.0	0.82 (0.78, 0.86)	40.3
Partner’s educational level (years)	23,939	17				
0–7			1.00 (Ref)		1.00 (Ref)	
8–11			0.87 (0.84, 0.90)	20.3	0.93 (0.90, 0.96)	0.0
≥12			0.74 (0.70, 0.79)	0.0	0.85 (0.79, 0.90)	0.0
Woman’s occupation	30,860	31				
Does not work outside home			1.00 (Ref.)		1.00 (Ref.)	
Agriculture/informal sector			0.99 (0.94, 1.05)	48.9	1.02 (0.96, 1.08)	17.3
Formal sector			0.84 (0.78, 0.89)	31.1	0.95 (0.89, 1.02)	0.0
Partner’s occupation	15,119	13				
Does not work outside home			1.00 (Ref.)	21.9	1.00 (Ref.)	
Agriculture/informal sector			1.14 (0.99, 1.32)	6.8	1.04 (0.88, 1.23)	0.0
Formal sector			0.89 (0.84, 0.94)		0.93 (0.87, 0.98)	0.0
Married/cohabiting	30,403	25	0.95 (0.90, 1.00)	49.8	0.98 (0.92, 1.04)	16.1
Parity (previous live births)	83,289	39				
0			1.00 (Ref)		1.00 (Ref)	
1			1.04 (1.02, 1.06)	46.8	1.03 (1.01, 1.06)	38.6
2			1.07 (1.05, 1.09)	53.7	1.05 (1.02, 1.08)	50.7
3			1.07 (1.04, 1.10)	69.0	1.04 (1.01, 1.07)	64.9
≥4			1.10 (1.07, 1.13)	86.1	1.07 (1.03, 1.11)	73.4
HIV positive	8,378	10	1.75 (1.68, 1.81)	91.5	1.46 (1.38, 1.56)	80.5
Chronic hypertension	57,117	28	1.25 (1.15, 1.35)	40.8	1.18 (1.07, 1.30)	28.1
Woman’s BMI	138,286	55				
Underweight			1.18 (1.16, 1.19)	97.2	1.18 (1.16, 1.20)	97.5
Normal weight			1.00 (Ref.)		1.00 (Ref.)	
Overweight/obese			0.74 (0.72, 0.76)	87.3	0.74 (0.72, 0.77)	86.9
Woman’s MUAC	36,260	25				
Underweight			1.39 (1.36, 1.43)	94.3	1.45 (1.41, 1.49)	96.4
Adequate			1.00 (Ref.)		1.00 (Ref.)	
Overweight/obese			0.66 (0.62, 0.69)	84.5	0.64 (0.61, 0.68)	83.1
Woman’s height (cm)	138,286	55				
<145			1.49 (1.47, 1.52)	86.9	1.38 (1.35, 1.41)	86.1
145–<150			1.25 (1.23, 1.28)	69.7	1.22 (1.19, 1.25)	69.0
150–<155			1.12 (1.10, 1.14)	54.3	1.10 (1.08, 1.12)	48.6
≥155			1.00 (Ref)		1.00 (Ref)	
Pre-pregnancy smoking	46,806	12	2.72 (2.54, 2.89)	84.3	1.94 (1.69, 2.23)	78.8
First-trimester smoking	34,873	19	1.62 (1.56, 1.68)	96.2	1.28 (1.22, 1.34)	97.2
Second-trimester smoking	20,743	12	1.18 (1.07, 1.31)	39.9	0.99 (0.98, 1.11)	97.9
Third-trimester smoking	8,019	9	1.06 (1.04, 1.08)	75.6	1.04 (1.02, 1.07)	98.5
First-trimester alcohol consumption	19,705	16	1.02 (0.96, 1.09)	30.0	1.01 (0.95, 1.08)	0.0
Second-trimester alcohol consumption	18,573	15	0.96 (0.90, 1.04)	0.0	0.97 (0.90, 1.05)	0.0
Third-trimester alcohol consumption	8,827	14	1.05 (1.03, 1.07)	11.2	1.04 (1.02, 1.07)	0.0
Any anemia during pregnancy	40,661	35	1.04 (1.02, 1.07)	39.2	1.04 (1.01, 1.06)	22.0
Any diarrhea during pregnancy	5,700	12	1.82 (1.74, 1.92)	84.5	1.51 (1.37, 1.65)	60.4
Any nausea during pregnancy	6,873	11	1.09 (1.03, 1.16)	0.0	1.11 (1.03, 1.18)	0.0
Any malaria before 36 weeks	18,780	14	1.15 (1.10, 1.21)	54.8	1.13 (1.07, 1.18)	51.1

BMI, body mass index; CI, confidence interval; cm, centimeter; HIV, human immunodeficiency virus; MUAC, mid-upper arm circumference; RR, risk ratio.

^1^Models of pre-pregnancy exposures adjusted for the following covariates, if available: age, woman’s education, partner’s education, woman’s occupation, partner’s occupation, marital status, parity, HIV status, chronic hypertension, woman’s BMI (except woman’s MUAC), and pre-pregnancy smoking. Models for exposures measured during pregnancy were additionally adjusted for season at 9 weeks of gestation and intervention where applicable.

^2^Defined as <70% of IOM recommendations.

**Table 3:** Two-stage pooled multivariable^1^ associations between participant characteristics and inadequate weight gain (*n* = 138,286)^2^.

	Number of participants	Number of studies	Crude RR (95% CI)	I^2^(%)	Multivariable-adjusted RR^1^ (95% CI)	I^2^(%)
**Characteristic**						
Woman’s age (years)	134,880	53				
<20			0.99 (0.99, 1.00)	67.5	0.99 (0.98, 1.00)	12.4
20–24			1.00 (Ref)		1.00 (Ref)	
25–29			0.99 (0.98, 1.00)	58.0	1.00 (0.99, 1.01)	30.3
30–34			0.98 (0.97, 1.00)	63.7	1.01 (0.99, 1.02)	34.4
≥35			1.04 (1.02, 1.05)	79.1	1.04 (1.02, 1.05)	56.8
Women’s educational level (years)	81,489	47				
0–7			1.00 (Ref)		1.00 (Ref)	
8–11			0.93 (0.92, 0.94)	65.6	0.96 (0.95, 0.97)	31.5
≥12			0.84 (0.82, 0.86)	79.6	0.91 (0.89, 0.94)	66.6
Partner’s educational level (years)	23,939	17				
0–7			1.00 (Ref)		1.00 (Ref)	
8–11			0.93 (0.91, 0.95)	0.0	0.96 (0.94, 0.98)	0.0
≥12			0.83 (0.80, 0.60)	0.0	0.90 (0.86, 0.93)	0.0
Woman’s occupation	30,860	31				
Does not work outside home			1.00 (Ref.)		1.00 (Ref.)	
Agriculture/informal sector			1.00 (0.97, 1.03)	63.9	1.03 (1.00, 1.06)	8.5
Formal sector			0.90 (0.86, 0.93)	28.7	0.99 (0.95, 1.03)	0.0
Partner’s occupation	15,119	13				
Does not work outside home			1.00 (Ref.)		1.00 (Ref.)	
Agriculture/informal sector			1.04 (0.96, 1.12)	0.0	0.98 (0.90, 1.07)	0.0
Formal sector			0.94 (0.91, 0.97)	0.0	0.96 (0.93, 0.99)	0.0
Married/cohabiting	30,403	25	0.82 (0.81, 0.83)	80.2	0.93 (0.90, 0.96)	27.6
Parity (previous live births)	83,289	39				
0			1.00 (Ref)		1.00 (Ref)	
1			1.02 (1.01, 1.03)	67.6	1.02 (1.01, 1.03)	59.8
2			1.01 (1.00, 1.02)	85.8	1.03 (1.02, 1.05)	52.6
3			1.01 (1.00, 1.02)	82.0	1.02 (1.00, 1.04)	68.1
≥4			1.03 (1.02, 1.04)	88.2	1.05 (1.03, 1.07)	66.1
HIV positive	8,378	10	1.29 (1.26, 1.32)	94.2	1.15 (1.11, 1.19)	81.5
Chronic hypertension	57,117	28	1.09 (1.04, 1.15)		1.10 (1.03, 1.16)	16.0
Woman’s BMI	138,286	55				
Underweight			1.13 (1.12, 1.14)	97.6	1.13 (1.12, 1.13)	97.8
Normal weight			1.00 (Ref.)		1.00 (Ref.)	
Overweight/obese			0.62 (0.60, 0.63)	93.8	0.61 (0.60, 0.63)	93.9
Woman’s MUAC	36,260	25				
Underweight			1.24 (1.23, 1.26)	93.1	1.25 (1.23, 1.27)	96.4
Adequate			1.00 (Ref.)		1.00 (Ref.)	
Overweight/obese			0.63 (0.61, 0.65)	91.3	0.62 (0.60, 0.64)	90.8
Woman’s height (cm)	138,286	55				
<145			1.07 (1.06, 1.09)	70.0	1.19 (1.18, 1.21)	93.1
145–<150			1.16 (1.15, 1.17)	87.5	1.14 (1.12, 1.15)	87.1
150–<155			1.21 (1.20, 1.22)	93.5	1.07 (1.05, 1.08)	65.8
≥155			1.00 (Ref)		1.00 (Ref)	
Pre-pregnancy smoking	46,806	12	1.36 (1.33, 1.38)	98.3	1.24 (1.18, 1.29)	86.1
First-trimester smoking	34,873	19	1.36 (1.34, 1.39)	97.7	1.13 (1.11, 1.16)	99.2
Second-trimester smoking	20,743	12	1.43 (1.37, 1.49)	96.4	0.99 (0.94, 1.05)	99.5
Third-trimester smoking	8,019	9	1.02 (1.01, 1.03)	76.5	1.01 (0.99, 1.02)	99.6
First-trimester alcohol consumption	19,705	16	0.99 (0.96, 1.03)	39.8	1.00 (0.96, 1.03)	8.5
Second-trimester alcohol consumption	18,573	15	0.99 (0.95, 1.03)	42.6	1.00 (0.96, 1.04)	0.0
Third-trimester alcohol consumption	8,827	14	1.02 (1.01, 1.04)	19.5	1.02 (1.01, 1.04)	9.3
Any anemia during pregnancy	40,661	35	1.03 (1.02, 1.05)	63.5	1.04 (1.02, 1.05)	56.4
Any diarrhea during pregnancy	5,700	12	1.24 (1.21, 1.27)	83.3	1.18 (1.13, 1.22)	0.0
Any nausea during pregnancy	6,873	11	1.04 (1.01, 1.08)	0.0	1.05 (1.01, 1.09)	0.0
Any malaria during before 36 weeks	18,780	14	1.09 (1.06, 1.12)	66.7	1.07 (1.04, 1.10)	70.3

BMI, body mass index; CI, confidence interval; cm, centimeter; HIV, human immunodeficiency virus; MUAC, mid-upper arm circumference; RR, risk ratio.

^1^Models of pre-pregnancy exposures adjusted for the following covariates, if available: age, woman’s education, partner’s education, woman’s occupation, partner’s occupation, marital status, parity, HIV status, chronic hypertension, woman’s BMI (except woman’s MUAC), and pre-pregnancy smoking. Models of exposures measured during pregnancy were additionally adjusted for season at 9 weeks of gestation and intervention where applicable.

^2^Defined as <90% of IOM recommendations ADMIN_MA Boston.

**Table 4:** Two-stage pooled multivariable^1^ associations between participant characteristics and excessive weight gain (*n =* 138,286)^2^.

	Number of participants	Number of studies	Crude RR^1^ (95% CI)	I^2^ (%)	Multivariable RR^1^ (95% CI)	I^2^ (%)
**Characteristic**						
Woman’s age (years)	134,880	53				
<20			0.95 (0.91, 1.00)	43.2	1.05 (1.01, 1.10)	0.0
20–24			1.00 (Ref)		1.00 (Ref)	
25–29			1.06 (1.03, 1.08)	53.6	0.96 (0.94, 0.99)	0.0
30–34			1.10 (1.07, 1.13)	72.6	0.93 (0.90, 0.96)	4.4
≥35			1.10 (1.06, 1.13)	69.1	0.87 (0.85, 0.90)	9.2
Women’s educational level (years)	81,489	47				
0–7					1.00 (Ref)	
8–11			1.47 (1.40, 1.54)	99.1	1.33 (1.26, 1.40)	99.0
≥12			1.58 (1.48, 1.68)	79.6	1.22 (1.14, 1.31)	30.6
Partner’s educational level (years)	23,939	17				
0–7			1.00 (Ref)		1.00 (Ref)	
8–11			1.33 (1.19, 1.49)	0.0	1.18 (1.04, 1.34)	14.7
≥12			1.68 (1.48, 1.92)	51.8	1.34 (1.14, 1.57)	23.2
Woman’s occupation	30,860	31				
Does not work outside home			1.00 (Ref.)		1.00 (Ref.)	
Agriculture/informal sector			1.11 (1.04, 1.20)	0.0	1.02 (0.95, 1.12)	0.0
Formal sector			1.20 (1.15, 1.26)	31.4	1.08 (1.02, 1.14)	22.2
Partner’s occupation	15,119	13				
Does not work outside home			1.00 (Ref.)		1.00 (Ref.)	
Agriculture/informal sector			0.99 (0.80, 1.22)	13.0	1.10 (0.90, 1.35)	0.7
Formal sector			1.29 (1.13, 1.47)	0.0	1.10 (0.97, 1.26)	0.3
Married/cohabiting	30,403	25	1.09	0.0	1.04 (0.97, 1.12)	0.0
Parity (previous live births)	83,289	39				
0			1.00 (Ref)		1.00 (Ref)	
1			0.96 (0.92, 1.00)	94.1	0.86 (0.82, 0.90)	92.0
2			1.12 (1.06, 1.19)	95.6	0.86 (0.80, 0.93)	93.6
3			1.07 (0.98, 1.17)	95.3	0.85 (0.77, 0.95)	93.9
≥4			1.16 (1.07, 1.25)	82.8	0.71 (0.63, 0.80)	18.2
HIV positive	8,378	10	2.54 (2.37, 2.73)	86.8	1.45 (1.23, 1.71)	67.0
Chronic hypertension	57,117	28	1.14 (1.32, 1.51)	75.4	0.97 (0.89, 1.06)	0.0
Woman’s BMI	138,286	55				
Underweight			0.53 (0.49, 0.58)	87.0	0.57 (0.54, 0.60)	79.3
Normal weight			1.00 (Ref.)		1.00 (Ref.)	
Overweight/obese			2.94 (2.81, 3.10)	92.2	2.55 (2.50, 2.60)	97.5
Woman’s MUAC	36,260	25				
Underweight			0.59 (0.56, 0.61)	78.5	0.51 (0.47, 0.56)	85.3
Adequate			1.00 (Ref.)		1.00 (Ref.)	
Overweight/obese			2.47 (2.43, 2.52)	98.3	3.02 (2.86, 3.19)	91.1
Woman’s height (cm)	138,286	55				
<145			0.85 (0.83, 0.87)	59.3	0.55 (0.50, 0.60)	77.9
145–<150			0.65 (0.63, 0.68)	69.8	0.67 (0.64, 0.70)	68.5
150–<155			0.82 (0.76, 0.88)	92.0	0.83 (0.86, 0.88)	56.2
≥155			1.00 (Ref)		1.00 (Ref)	
Pre-pregnancy smoking	46,806	12	1.19 (1.06, 1.34)	61.7	1.20 (1.07, 1.36)	3.8
First-trimester smoking	34,873	19	0.76 (0.69, 0.85)	62.9	0.69 (0.61, 0.78)	27.2
Second-trimester smoking	20,743	12	0.97 (0.80, 1.16)	5.7	0.89 (0.70, 1.14)	0.0
Third-trimester smoking	8,019	9	0.85 (0.70, 1.03)	14.2	0.75 (0.58, 0.97)	0.0
First-trimester alcohol consumption	19,705	16	1.39 (1.29, 1.50)	92.0	1.08 (0.98, 1.18)	54.3
Second-trimester alcohol consumption	18,573	15	1.19 (1.10, 1.29)	64.7	1.08 (0.99, 1.18)	31.7
Third-trimester alcohol consumption	8,827	14	0.92 (0.81, 1.03)	0.0	0.89 (0.78, 1.02)	0.0
Any anemia during pregnancy	40,661	35	0.80 (0.76, 0.83)	47.9	0.80 (0.76, 0.84)	81.5
Any diarrhea during pregnancy	5,700	12	1.35 (0.99, 1.85)	0.0	1.32 (0.94, 1.86)	0.0
Any nausea during pregnancy	6,873	11	1.07 (0.93, 1.22)	0.0	1.07 (0.93, 1.04)	95.4
Any malaria before 36 weeks of gestation	18,780	14	0.93 (0.83, 1.05)	0.0	0.96 (0.85, 1.08)	0.0

BMI, body mass index; CI, confidence interval; cm, centimeter; HIV, human immunodeficiency virus; MUAC, mid-upper arm circumference; RR, risk ratio.

^1^Models of pre-pregnancy exposures adjusted for the following covariates, if available: age, woman’s education, partner’s education, woman’s occupation, partner’s occupation, marital status, parity, HIV status, chronic hypertension, woman’s BMI (except woman’s MUAC), and pre-pregnancy smoking. Models of exposures measured during pregnancy were additionally adjusted for season at 9 weeks of gestation and intervention where applicable.

^2^Defined as >125% of IOM recommendations.

Parity and age showed associations with GWG outcomes solely in the 2-stage analyses. Having had 4 or more previous live births was associated with a marginally higher risk of severely inadequate GWG (RR 1.07, 95% CI [1.03, 1.11]) compared to those with no previous live births, but the heterogeneity of this pooled estimate was high (I^2^ = 73.4). A reduction in the risk of excessive GWG (RR 0.71, 95% CI [0.63, 0.80]) was observed among those in this category. While adolescents had a marginally higher risk of excessive GWG (RR 1.05, 95% CI [1.01, 1.10]) compared to those aged 20 to 24, those aged 35 and older had a marginally lower risk (RR 0.87, 95%% CI [0.85, 0.90]).

### Anthropometric risk factors

Participants with underweight based on early pregnancy BMI or first MUAC measurement had higher risks of inadequate and severely inadequate GWG compared to participants with normal weight, but underweight classification based on MUAC showed stronger associations with these outcomes (RR 1.25, 95% CI [1.23, 1.27] and RR 1.45, 95% CI [1.41, 1.49], respectively). Participants with overweight/obesity based on early pregnancy BMI or first MUAC measurement had lower risks of inadequate and severely inadequate GWG but substantially higher risks of excessive GWG compared to those with normal weight (RR 2.55, 95% CI [2.50, 2.60] for BMI ≥25 and RR 3.02, 95% CI [2.86, 3.19] for MUAC ≥28.1), but the large I^2^ values for these models indicated considerable heterogeneity. Height of <145 cm was associated with a higher risk for inadequate (RR 1.19, 95% CI [1.18, 1.21]) and severely inadequate GWG (1.38, 95% CI [1.35, 1.41]) and a lower risk for excessive GWG (RR 0.55, 95% CI [0.50, 0.60]), but corresponding I^2^ values were similarly large. Comparable, though attenuated, associations between these anthropometric risk factors and GWG outcomes were observed in the 1-stage analyses.

### Substance use risk factors

Although only a minority of studies collected information about smoking status (22%, 35%, 22%, and 1% of studies for pre-pregnancy, first trimester, second trimester, and third trimester, respectively), participants who reported any pre-pregnancy smoking were more likely to experience inadequate (RR 1.24, 95% [1.19, 1.28]), severely inadequate (RR 1.94, 95% CI [1.69, 2.23]), and excessive GWG (RR 1.20, 95% CI [1.07, 1.36]). Any smoking during the first trimester was similarly associated with a higher risk of severely inadequate GWG (RR 1.28, 95% CI [1.22, 1.34]) but a lower risk of excessive GWG (RR 0.69, 95% CI [0.61, 0.78]). I^2^ values were high for these associations, however, and they were somewhat attenuated in the 1-stage analyses.

### Clinical risk factors

Participants living with HIV infection had an increased risk of inadequate (RR 1.15, 95% CI [1.11, 1.19]) and severely inadequate GWG (RR 1.46, 95% CI [1.38, 1.56]), though the I^2^ values exceeded 80% for these models. These associations were present, though more modest in the 1-stage models. Participants living with HIV infection also had an increased risk of excessive GWG (RR 1.45, 95% CI [1.23, 1.71]) that was not observed in the 1-stage analysis. Participants who were anemic at any point during pregnancy had a lower risk of excessive GWG (RR 0.80, 95% CI [0.76, 0.84]), but this association was not shown in the 1-stage analysis. Participants who reported any diarrhea or any nausea during pregnancy had an increased risk of severely inadequate GWG (RR 1.51, 95% CI [1.37, 1.65] and 1.11, 95% CI [1.03, 1.18], respectively) compared to participants who did not, but these associations were somewhat attenuated in the 1-stage analysis. Those diagnosed with malaria before 36 weeks of gestation had an increased risk of inadequate weight gain (RR 1.07, 95% CI [1.04, 1.10]) and severely inadequate weight gain (RR 1.13, 95% CI [1.07, 1.18]) with high heterogeneity. Similar results were observed in the 1-stage analyses.

### Effect measure modification by BMI and sensitivity analyses

The association between most risk factors and inadequate GWG, severely inadequate GWG, or excessive GWG did not differ substantially by first-trimester BMI category (Appendix Figures D1-L2 in S1 Appendix). Most notably, HIV infection was associated with a lower risk of excessive GWG only among participants with overweight or obesity (RR 0.78, 95% CI [0.68, 0.89]). Another difference was the presence of a positive association between chronic hypertension and severely inadequate GWG among participants with underweight (RR 1.16, 95% CI [1.06, 1.28]) and normal weight (RR 1.14, 95% CI [1.07, 1.22]) but a negative association among those with overweight/obesity (RR 0.96, 95% CI [0.92, 1.00]).

Associations between the various risk factors and outcomes were largely of similar direction to the overall 1-stage analysis results when using the INTERGROWTH-21st standards to categorize low GWG among women with normal weight, though some attenuation in magnitude was seen for risk factors such as height and MUAC that had shown some of the strongest associations with severely inadequate and inadequate weight gain among women with normal weight in the 1-stage analyses that defined GWG using IOM recommendations (Figures M1-N2 S1 Appendix). On the other hand, whereas participants with normal weight and MUAC measurements <24.0 cm had a slightly lower risk for excessive GWG (RR 0.95, 95% CI [0.95, 0.96]) as defined by IOM recommendations compared to those with MUAC measurements between 24.0 and 28.1, they had a higher risk of a GWG z-score as defined by the INTERGROWTH-21st standards (RR 1.08, 95% CI [1.06, 1.09]) (Figures O1-O2 in S1 Appendix). Limiting the analyses to participants with weight measures in the third trimester also led to the attenuation of some associations, as did using the lower limits of the IOM recommended mean rate of weight gain in the second and third trimester rather than the mean itself to define expected weight gain, but most remained present (Figures P1-U2 in S1 Appendix). Applying a lower cutoff of >23 kg/m^2^ to define overweight among Asian participants produced minimal changes to the results (Figures V1-X2 in S1 Appendix). Restricting the reference category for each outcome to those with adequate GWG led to some attenuation of most relative risks, but the overall trends remained consistent (Figures Y1-AA2 in S1 Appendix). These trends also largely remained consistent when we limited the analytic cohort to those who had not received any interventions (Figures BB1-DD2 in S1 Appendix).

## Discussion

This large-scale assessment of risk factors for inadequate and excessive GWG among 145,949 women from 55 studies in LMICs found that over half (54%) of women experienced inadequate GWG and approximately one-third (34%) experienced severely inadequate GWG. Excessive GWG was observed among 22% of pregnant women. Anthropometric factors such as BMI, MUAC, and height were strongly associated with all 3 outcomes. We also observed that smoking and HIV infection were associated with a higher risk of inadequate and severely inadequate weight gain, while higher levels of education were associated with a lower risk. Higher levels of education were also associated with a higher risk of excessive weight gain.

Although both women with underweight and women with a MUAC measurement of <24 cm had an increased risk of inadequate and severely inadequate weight gain, a MUAC measurement of <24 cm was associated with a larger increase. MUAC is a useful measure of undernutrition not only because it changes little over pregnancy [92] but also because it is easy and fast to measure using only a simple tool. At the same time, the interpretability of this measurement may be limited given that the ratio of its components (bone, muscle, and fat) may differ between populations and age groups.

Our finding that participants with first-trimester overweight or obesity had a substantially increased risk of excessive weight gain agrees with multiple previous reports [9,10,12,15,16,18,19,21,22]. Potential mechanisms for this link include lower levels of resting energy expenditure in this group [93,94] and a higher likelihood of developing complications that lead to increased weight gain such as gestational hypertension [10,12]. At the same time, these women may be more likely to exceed recommendations because the recommendations themselves are lower for women in this category. We also observed that MUAC measurements of <24 cm were associated with an increased risk of INTERGROWTH-21st GWG z-scores of >1 among women with normal weight, which suggests that high GWG may be compensatory in the context of low nutritional stores.

Although being underweight was associated with a higher risk of severely inadequate weight gain, short stature (<145 cm) showed a somewhat stronger association in models that were adjusted for maternal early pregnancy BMI. An association between short stature and lower GWG has been observed previously in both HIC and LMIC contexts [13,95–97]. Because short stature may be an indicator of chronic undernutrition during the intrauterine period, childhood, and early adolescence, these findings emphasize the long-term, cumulative, and potentially intergenerational impact of nutritional deficiencies [6]. At the same time, this finding raises questions about whether future GWG guidelines should take stature into account given that those of short stature are underrepresented in the current guidelines.

Previous studies have indicated that younger mothers are at greater risk of both inadequate [8,11,12] and excessive weight gain [11,12,18]. Our results are more in line with the latter finding. This finding may be attributable to temporal improvements in female educational status and SES that have led to increased access to energy-dense foods and more sedentary lifestyles, though we were unable to examine temporal trends in this analysis. Because adolescents, who account for up to 30% to 40% of all pregnancies in parts of sub-Saharan Africa and Southeast Asia [98], may be at an increased risk for excessive weight gain, they may benefit from increased weight monitoring, nutritional education, and nutritional and physical activity interventions. It should also be noted, however, that some adolescents in this study were still in the phase of linear growth, and existing GWG recommendations do not account for the increased nutritional requirements among adolescents in this phase that may lead them to gain a larger than recommended amount of weight.

Our results are consistent with previous literature showing that lower educational status is associated with a greater risk for inadequate GWG [9,13,14,17]. In our analysis, education level was the indicator of SES most frequently measured across studies. Lack of access to education may be a manifestation of wealth inequalities [99] that also impede access to adequate nutrition and healthcare, which may, in turn, contribute to lower weight gain. At the other end of the educational spectrum, however, having 12 or more years of education was associated with a higher risk of excessive weight gain, which suggests that socioeconomic advantage alone does not protect against deviations from healthy GWG, and more targeted education regarding healthy weight gain during pregnancy may be needed across the socioeconomic gradient in LMICs.

Multiparity was associated with only a slightly higher risk of severely inadequate GWG, but a more substantial reduction in the risk of excessive GWG. Previous findings regarding parity and GWG are conflicting in both HICs and LMICs. A meta-analysis reported both positive and negative relationships between parity and GWG [100]. The authors of the meta-analysis concluded that parity likely has an indirect, complex association with GWG that may be mediated by weight gain in prior pregnancies, interpregnancy interval, and other factors associated with entering parenthood, such as alterations in diet and physical activity. A hypothesized explanation for the inverse association between parity and excessive GWG that we observed is that an adaptive physiological or metabolic response may take place during a first pregnancy that reduces the amount of weight gain required for subsequent pregnancies [101].

A pooled study of Demographic and Health Survey data has suggested that the prevalence of smoking among pregnant women in LMICs is low overall but varies widely between countries, and the authors noted that the tobacco industry has been expanding marketing efforts that target women of reproductive age in these settings [102]. In our study, 12% of participants reported smoking in studies that collected this information. Our observation that smoking during the pre-pregnancy and pregnancy period is associated with a higher risk of inadequate and severely inadequate GWG is, therefore, relevant. Smoking has previously been linked to inadequate weight gain [9], and though potential mechanisms for this association are not well understood, it may relate to appetite suppression caused by nicotine [103]. We were unable to measure exposure to smokeless tobacco; smokeless tobacco use is reported by an estimated 10.4% (SD 8.9) of females in LMICs [104], and such products may have a higher nicotine content than cigarettes [105], so future studies would benefit from including assessments of their use. Ultimately, our findings emphasize the need for smoking cessation initiatives during pregnancy in LMICs. We were also unable to measure exposure to indoor air pollution from cooking stoves, which is common in LMICs [106] and another important area for further investigation.

Our findings also build on those from the few previous studies that have examined the association between maternal comorbidities and GWG. That HIV infection was associated with an increased risk of inadequate and severely inadequate GWG is consistent with a study from South Africa [107], which observed this finding independent of antiretroviral therapy (ART) initiation. We were unable to account for ART status in this analysis, but previous studies have shown that average weight gain was far below IOM recommendations among pregnant women with HIV who received ART and women with HIV whose pregnancies occurred in the pre-ART era [108,109]. This association is biologically plausible since HIV infection is known to interfere with nutrient absorption and metabolism [110].

We additionally observed a higher risk of excessive weight gain among women with HIV infection. Though this association was largely influenced by 1 study and was not present in the 1-stage analyses, it is also somewhat concordant with previous findings. A retrospective cohort study from the United States found that newer ART regimens are associated with an increased risk of excessive GWG [111]. Overall, our findings regarding HIV infection highlight the need for additional research examining how to support optimal GWG in this group. Malaria infection during pregnancy also appeared to increase the risk of inadequate and severely inadequate weight gain, which is in line with an observation that malaria infection was associated with a lower rate of weight gain during the second trimester among HIV-infected pregnant women in Tanzania [109]. This suggests a possible role for malaria prevention measures in strategies to improve pregnancy weight gain.

Strengths of the study include the large sample size and participants from diverse populations in LMICs and the use of rigorous methodology to define and model GWG independent of gestational duration. However, some limitations should be noted. First, the 50% response rate among contacted investigators could have introduced bias if the associations between these risk factors and GWG differ in datasets that were not contributed to the project. Second, the considerable degree of heterogeneity in measures used across studies meant that categorization of risk factors was necessarily broad. The variability within some exposure categories may have attenuated observed associations and may also be reflected in the high degree of heterogeneity in results across studies observed for some risk factors. One major source of this heterogeneity was the differences in potential confounders that could be included in multivariable models across studies, which is a limitation of all meta-analyses of observational data. An analysis of the sources of heterogeneity was beyond the scope of the present study. Such heterogeneity warrants some caution in the interpretation of our findings, though they are largely consistent with previous literature. Second, many risk factors of interest were measured in only a few studies, which limits the precision and generalizability of the results. Third, the lack of detailed data on socioeconomic factors across studies may have led to some residual confounding. Fourth, we were unable to evaluate factors that may be more proximally related to energy balance, such as dietary intake, physical activity, and psychosocial factors, due to the difficulty of collecting and harmonizing these data within the timeframe of the project. Future analyses of these factors are planned.

Fifth, the imputed first trimester weight values likely introduced some error into the classification of GWG category, especially since missingness was somewhat associated with BMI category. Imputed weights were used for 32% of women with underweight, 37% of women with normal weight, and 25% of women with overweight or obesity, which may have led to more women with normal weight being placed in an incorrect BMI category and having had an incorrect expected rate of GWG applied to their adequacy ratio calculation. We believe that these errors would have resulted in an increased similarity between participants in the outcome and reference categories with respect to GWG and therefore caused us to underestimate the associations between each risk factor and GWG outcome.

Lastly, the applicability of the IOM guidelines to women in LMICs may be questionable, since such women are not represented in those guidelines. In a sensitivity analysis in which we used the lower limit of the mean recommended second and third trimester gain to define expected GWG, however, our results were similar to those of the main 1-stage analysis. Furthermore, our findings were for the most part consistent for GWG defined by the INTERGROWTH-21st among women with normal weight. Low MUAC, however, showed opposite associations with excessive GWG and an INTERGROWTH-21st GWG z-score of >1 in this group. The discrepancy between these findings highlights the conceptual differences between these outcomes. Whereas INTERGROWTH-21st GWG z-scores measure GWG compared to a geographically diverse population standard, the IOM categorizations measure GWG compared to an expected amount of gain based on BMI category with the assumptions that expected GWG does not differ within a given category. Given the wide variations in body composition that have been observed in different geographic areas [112] that likely reflect a combination of genetic, epigenetic, and environmental influences, the assumption used for IOM guidelines, which were developed based on populations from HICs only, may not hold across all populations. A robust meta-analysis of over 1.4 million pregnancies demonstrated, however, that GWG outside IOM guidelines is associated with adverse outcomes among women in East Asia as well as those in the USA and Europe [3]. These findings lend support for applying IOM recommendations across geographic regions. Still, future research is needed to determine healthy weight gain ranges for all body sizes that are applicable to all areas of the globe.

We conclude that inadequate GWG is a major public health concern in LMICs, and several demographic, nutritional, substance use, and clinical factors may perpetuate its occurrence. Thus, our results suggest that comprehensive interventions to improve maternal health and nutrition status and promote healthy behaviors are needed. Since long-term nutritional status as measured by short maternal stature was strongly related to inadequate and severely inadequate GWG, efforts should be made to improve nutritional status before childbearing is initiated, probably beginning in childhood and adolescence. The extent of excessive GWG and its determinants is also a public health concern and warrants additional research.

## Supporting information

S1 Text List of GWG Pooling Project Consortium Members. (DOCX)

S1 PRISMA Checklist PRISMA 2020 Checklist. (DOCX)

S2 PRISMA Checklist PRISMA 2020 for Abstracts Checklist. (DOCX)

S1 Appendix Supporting information. Table A. Systematic search strategy. Table B. Interventions received in trials included in pooled analyses. Table C. Overall participant characteristics. Figure A1. Unadjusted RRs and 95% CIs for the associations between demographic, anthropometric, substance use, and clinical risk factors and severely inadequate GWG (1-stage model, *n* = 79,948). Circles represent RRs and bars represent 95% CIs. BMI, body mass index; CI, confidence interval; cm, centimeter; GWG, gestational weight gain; HIV, human immunodeficiency virus; MUAC, mid-upper arm circumference; RR, risk ratio. Figure A2. Adjusted RRs and 95% CIs for the associations between demographic, anthropometric, substance use, and clinical risk factors and severely inadequate GWG (1-stage model, *n =* 79,948). Circles represent RRs and bars represent 95% CIs. BMI, body mass index; CI, confidence interval; cm, centimeter; GWG, gestational weight gain; HIV, human immunodeficiency virus; MUAC, mid-upper arm circumference; RR, risk ratio. Figure B1. Unadjusted RRs and 95% CIs for the associations between demographic, anthropometric, substance use, and clinical risk factors and inadequate GWG (1-stage model, *n =* 79,948). Circles represent RRs and bars represent 95% CIs. BMI, body mass index; CI, confidence interval; cm, centimeter; GWG, gestational weight gain; HIV, human immunodeficiency virus; MUAC, mid-upper arm circumference; RR, risk ratio. Figure B2. Adjusted RRs and 95% CIs for the associations between demographic, anthropometric, substance use, and clinical risk factors and inadequate GWG (1-stage model, *n =* 79,948). Circles represent RRs and bars represent 95% CIs. BMI, body mass index; CI, confidence interval; cm, centimeter; GWG, gestational weight gain; HIV, human immunodeficiency virus; MUAC, mid-upper arm circumference; RR, risk ratio. Figure C1. Unadjusted RRs and 95% CIs for the associations between demographic, anthropometric, substance use, and clinical risk factors and excessive GWG (1-stage model, *n =* 79,948). Circles represent RRs and bars represent 95% CIs. BMI, body mass index; CI, confidence interval; cm, centimeter; GWG, gestational weight gain; HIV, human immunodeficiency virus; MUAC, mid-upper arm circumference; RR, risk ratio. Figure C2. Adjusted RRs and 95% CIs for the associations between demographic, anthropometric, substance use, and clinical risk factors and excessive GWG (1-stage model, *n =* 79,948). Circles represent RRs and bars represent 95% CIs. BMI, body mass index; CI, confidence interval; cm, centimeter; GWG, gestational weight gain; HIV, human immunodeficiency virus; MUAC, mid-upper arm circumference; RR, risk ratio. Figure D1. Unadjusted RRs and 95% CIs for the associations between demographic, anthropometric, and clinical risk factors and severely inadequate GWG (1-stage model) among participants with underweight (*n =* 19,735). Circles represent RRs and bars represent 95% CIs. BMI, body mass index; CI, confidence interval; cm, centimeter; GWG, gestational weight gain; HIV, human immunodeficiency virus; MUAC, mid-upper arm circumference; RR, risk ratio. Figure D2. Adjusted RRs and 95% CIs for the associations between demographic, anthropometric, and clinical risk factors and severely inadequate GWG (1-stage model) among participants with underweight (*n =* 19,735). Circles represent RRs and bars represent 95% CIs. BMI, body mass index; CI, confidence interval; cm, centimeter; GWG, gestational weight gain; HIV, human immunodeficiency virus; MUAC, mid-upper arm circumference; RR, risk ratio. Figure E1. Unadjusted RRs and 95% CIs for the associations between demographic, anthropometric, and clinical risk factors and inadequate GWG (1-stage model) among participants with underweight. Circles represent RRs and bars represent 95% CIs. BMI, body mass index; CI, confidence interval; cm, centimeter; GWG, gestational weight gain; HIV, human immunodeficiency virus; MUAC, mid-upper arm circumference; RR, risk ratio. Figure E2. Adjusted RRs and 95% CIs for the associations between demographic, anthropometric, and clinical risk factors and inadequate GWG (1-stage model) among participants with underweight (*n =* 19,735). Circles represent RRs and bars represent 95% CIs. BMI, body mass index; CI, confidence interval; cm, centimeter; GWG, gestational weight gain; HIV, human immunodeficiency virus; MUAC, mid-upper arm circumference; RR, risk ratio. Figure F1. Unadjusted RRs and 95% CIs for the associations between demographic, anthropometric, and clinical risk factors and excessive GWG (1-stage model) among participants with underweight (*n =* 19,735). Circles represent RRs and bars represent 95% CIs. BMI, body mass index; CI, confidence interval; cm, centimeter; GWG, gestational weight gain; HIV, human immunodeficiency virus; MUAC, mid-upper arm circumference; RR, risk ratio. Figure F2. Adjusted RRs and 95% CIs for the associations between demographic, anthropometric, and clinical risk factors and excessive GWG (1-stage model) among participants with underweight (*n =* 19,735). Circles represent RRs and bars represent 95% CIs. BMI, body mass index; CI, confidence interval; cm, centimeter; GWG, gestational weight gain; HIV, human immunodeficiency virus; MUAC, mid-upper arm circumference; RR, risk ratio. Figure G1. Unadjusted RRs and 95% CIs for the associations between demographic, anthropometric, and clinical risk factors and severely inadequate GWG (1-stage model) among participants with normal weight (*n =* 51,047). Circles represent RRs and bars represent 95% CIs. BMI, body mass index; CI, confidence interval; cm, centimeter; GWG, gestational weight gain; HIV, human immunodeficiency virus; MUAC, mid-upper arm circumference; RR, risk ratio. Figure G2. Adjusted RRs and 95% CIs for the associations between demographic, anthropometric, and clinical risk factors and severely inadequate GWG (1-stage model) among participants with normal weight (*n =* 51,047). Circles represent RRs and bars represent 95% CIs. BMI, body mass index; CI, confidence interval; cm, centimeter; GWG, gestational weight gain; HIV, human immunodeficiency virus; MUAC, mid-upper arm circumference; RR, risk ratio. Figure H1. Unadjusted RRs and 95% CIs for the associations between demographic, anthropometric, and clinical risk factors and inadequate GWG (1-stage model) among participants with normal weight (*n =* 51,047). Circles represent RRs and bars represent 95% CIs. BMI, body mass index; CI, confidence interval; cm, centimeter; GWG, gestational weight gain; HIV, human immunodeficiency virus; MUAC, mid-upper arm circumference; RR, risk ratio. Figure H2. Adjusted RRs and 95% CIs for the associations between demographic, anthropometric, and clinical risk factors and inadequate GWG (1-stage model) among participants with normal weight (*n =* 51,047). Circles represent RRs and bars represent 95% CIs. BMI, body mass index; CI, confidence interval; cm, centimeter; GWG, gestational weight gain; HIV, human immunodeficiency virus; MUAC, mid-upper arm circumference; RR, risk ratio. Figure I1. Unadjusted RRs and 95% CIs for the associations between demographic, anthropometric, and clinical risk factors and excessive GWG (1-stage model) among participants with normal weight (*n =* 51,047). Circles represent RRs and bars represent 95% CIs. BMI, body mass index; CI, confidence interval; cm, centimeter; GWG, gestational weight gain; HIV, human immunodeficiency virus; MUAC, mid-upper arm circumference; RR, risk ratio. Figure I2. Adjusted RRs and 95% CIs for the associations between demographic, anthropometric, and clinical risk factors and excessive GWG (1-stage model) among participants with normal weight (*n =* 51,047). Circles represent RRs and bars represent 95% CIs. BMI, body mass index; CI, confidence interval; cm, centimeter; GWG, gestational weight gain; HIV, human immunodeficiency virus; MUAC, mid-upper arm circumference; RR, risk ratio. Figure J1in S1 Appendix. Unadjusted RRs and 95% CIs for the associations between demographic, anthropometric, and clinical risk factors and severely inadequate GWG (1-stage model) among participants with overweight and obesity. Circles represent RRs and bars represent 95% CIs. BMI, body mass index; CI, confidence interval; cm, centimeter; GWG, gestational weight gain; HIV, human immunodeficiency virus; MUAC, mid-upper arm circumference; RR, risk ratio. Figure J2. Adjusted RRs and 95% CIs for the associations between demographic, anthropometric, and clinical risk factors and severely inadequate GWG (1-stage model) among participants with overweight and obesity (*n =* 9,166). Circles represent RRs and bars represent 95% CIs. BMI, body mass index; CI, confidence interval; cm, centimeter; GWG, gestational weight gain; HIV, human immunodeficiency virus; MUAC, mid-upper arm circumference; RR, risk ratio. Figure K1. Unadjusted RRs and 95% CIs for the associations between demographic, anthropometric, and clinical risk factors and inadequate GWG (1-stage model) among participants with overweight and obesity (*n =* 9,166). Circles represent RRs and bars represent 95% CIs. BMI, body mass index; CI, confidence interval; cm, centimeter; GWG, gestational weight gain; HIV, human immunodeficiency virus; MUAC, mid-upper arm circumference; RR, risk ratio. Figure K2. Adjusted RRs and 95% CIs for the associations between demographic, anthropometric, and clinical risk factors and inadequate GWG (1-stage model) among participants with overweight and obesity (*n =* 9,166). Circles represent RRs and bars represent 95% CIs. BMI, body mass index; CI, confidence interval; cm, centimeter; GWG, gestational weight gain; HIV, human immunodeficiency virus; MUAC, mid-upper arm circumference; RR, risk ratio. Figure L1. Unadjusted RRs and 95% CIs for the associations between demographic, anthropometric, and clinical risk factors and excessive GWG (1-stage model) among participants with overweight and obesity (*n =* 9,166). Circles represent RRs and bars represent 95% CIs. BMI, body mass index; CI, confidence interval; cm, centimeter; GWG, gestational weight gain; HIV, human immunodeficiency virus; MUAC, mid-upper arm circumference; RR, risk ratio. Figure L2. Adjusted RRs and 95% CIs for the associations between demographic, anthropometric, and clinical risk factors and excessive GWG (1-stage model) among participants with overweight and obesity (*n =* 9,166). Circles represent RRs and bars represent 95% CIs. BMI, body mass index; CI, confidence interval; cm, centimeter; GWG, gestational weight gain; HIV, human immunodeficiency virus; MUAC, mid-upper arm circumference; RR, risk ratio. Figure M1. Unadjusted RRs and 95% CIs for the associations between demographic, anthropometric, and clinical risk factors and weight gain z-score <−2 based on the INTERGROWTH-21st standard (1-stage model) among women with normal weight (*n =* 51,047). Circles represent RRs and bars represent 95% CIs. BMI, body mass index; CI, confidence interval; cm, centimeter; GWG, gestational weight gain; HIV, human immunodeficiency virus; MUAC, mid-upper arm circumference; RR, risk ratio. Figure M2. Adjusted RRs and 95% CIs for the associations between demographic, anthropometric, and clinical risk factors and weight gain z-score <−2 based on the INTERGROWTH-21st standard (1-stage model) among women with normal weight (*n =* 51,047). Circles represent RRs and bars represent 95% CIs. BMI, body mass index; CI, confidence interval; cm, centimeter; GWG, gestational weight gain; HIV, human immunodeficiency virus; MUAC, mid-upper arm circumference; RR, risk ratio. Figure N1. Adjusted RRs and 95% CIs for the associations between demographic, anthropometric, and clinical risk factors and weight gain z-score <−1 based on the INTERGROWTH-21st standard (1-stage model) among women with normal weight (*n =* 51,047). Circles represent RRs and bars represent 95% CIs. BMI, body mass index; CI, confidence interval; cm, centimeter; GWG, gestational weight gain; HIV, human immunodeficiency virus; MUAC, mid-upper arm circumference; RR, risk ratio. Figure N2. Unadjusted RRs and 95% CIs for the associations between demographic, anthropometric, and clinical risk factors and weight gain z-score <−1 based on the INTERGROWTH-21st standard (1-stage model) among women with normal weight (*n =* 51,047). Circles represent RRs and bars represent 95% CIs. BMI, body mass index; CI, confidence interval; cm, centimeter; GWG, gestational weight gain; HIV, human immunodeficiency virus; MUAC, mid-upper arm circumference; RR, risk ratio. Figure O1. Unadjusted RRs and 95% CIs for the associations between demographic, anthropometric, and clinical risk factors and weight gain z-score >1 based on the INTERGROWTH-21st standard (1-stage model) among women with normal weight (*n =* 51,047). Circles represent RRs and bars represent 95% CIs. BMI, body mass index; CI, confidence interval; cm, centimeter; GWG, gestational weight gain; HIV, human immunodeficiency virus; MUAC, mid-upper arm circumference; RR, risk ratio. Figure O2. Adjusted RRs and 95% CIs for the associations between demographic, anthropometric, and clinical risk factors and weight gain z-score >1 based on the INTERGROWTH-21st standard (1-stage model) among women with normal weight (*n =* 51,047). Circles represent RRs and bars represent 95% CIs. BMI, body mass index; CI, confidence interval; cm, centimeter; GWG, gestational weight gain; HIV, human immunodeficiency virus; MUAC, mid-upper arm circumference; RR, risk ratio. Figure P1. Unadjusted RRs and 95% CIs for the associations between demographic, anthropometric, substance use, and clinical risk factors and severely inadequate GWG (1-stage model) among women with a third trimester weight measurement. Circles represent RRs and bars represent 95% CIs. BMI, body mass index; CI, confidence interval; cm, centimeter; GWG, gestational weight gain; HIV, human immunodeficiency virus; MUAC, mid-upper arm circumference; RR, risk ratio. Figure P2. Adjusted RRs and 95% CIs for the associations between demographic, anthropometric, substance use, and clinical risk factors and severely inadequate GWG (1-stage model) among women with a third trimester weight measurement (*n =* 69,659). Circles represent RRs and bars represent 95% CIs. BMI, body mass index; CI, confidence interval; cm, centimeter; GWG, gestational weight gain; HIV, human immunodeficiency virus; MUAC, mid-upper arm circumference; RR, risk ratio. Figure Q1. Unadjusted RRs and 95% CIs for the associations between demographic, anthropometric, substance use, and clinical risk factors and inadequate GWG (1-stage model) among women with a third trimester weight measurement (*n =* 69,659). Circles represent RRs and bars represent 95% CIs. BMI, body mass index; CI, confidence interval; cm, centimeter; GWG, gestational weight gain; HIV, human immunodeficiency virus; MUAC, mid-upper arm circumference; RR, risk ratio. Figure Q2. Adjusted RRs and 95% CIs for the associations between demographic, anthropometric, substance use, and clinical risk factors and inadequate GWG (1-stage model) among women with a third trimester weight measurement (*n =* 69,659). Circles represent RRs and bars represent 95% CIs. BMI, body mass index; CI, confidence interval; cm, centimeter; GWG, gestational weight gain; HIV, human immunodeficiency virus; MUAC, mid-upper arm circumference; RR, risk ratio. Figure R1. Unadjusted RRs and 95% CIs for the associations between demographic, anthropometric, substance use, and clinical risk factors and excessive GWG (1-stage model) among women with a third trimester weight measurement (*n =* 69,659). Circles represent RRs and bars represent 95% CIs. BMI, body mass index; CI, confidence interval; cm, centimeter; GWG, gestational weight gain; HIV, human immunodeficiency virus; MUAC, mid-upper arm circumference; RR, risk ratio. Figure R2. Adjusted RRs and 95% CIs for the associations between demographic, anthropometric, substance use, and clinical risk factors and excessive GWG (1-stage model) among women with a third trimester weight measurement (*n =* 69,659). Circles represent RRs and bars represent 95% CIs. BMI, body mass index; CI, confidence interval; cm, centimeter; GWG, gestational weight gain; HIV, human immunodeficiency virus; MUAC, mid-upper arm circumference; RR, risk ratio. Figure S1. Unadjusted RRs and 95% CIs for the associations between demographic, anthropometric, substance use, and clinical risk factors and severely inadequate GWG (1-stage model) using the lower limit of the IOM recommendations to calculate expected GWG (*n =* 79,748). Circles represent RRs and bars represent 95% CIs. BMI, body mass index; CI, confidence interval; cm, centimeter; GWG, gestational weight gain; HIV, human immunodeficiency virus; MUAC, mid-upper arm circumference; RR, risk ratio. Figure S2. Adjusted RRs and 95% CIs for the associations between demographic, anthropometric, substance use, and clinical risk factors and severely inadequate GWG (1-stage model) using the lower limit of the IOM recommendations to calculate expected GWG (*n =* 79,748). Circles represent RRs and bars represent 95% CIs. BMI, body mass index; CI, confidence interval; cm, centimeter; GWG, gestational weight gain; HIV, human immunodeficiency virus; MUAC, mid-upper arm circumference; RR, risk ratio. Figure T1. Unadjusted RRs and 95% CIs for the associations between demographic, anthropometric, substance use, and clinical risk factors and inadequate GWG (1-stage model) using the lower limit of the IOM recommendations to calculate expected GWG (*n =* 79,948). Circles represent RRs and bars represent 95% CIs. BMI, body mass index; CI, confidence interval; cm, centimeter; GWG, gestational weight gain; HIV, human immunodeficiency virus; MUAC, mid-upper arm circumference; RR, risk ratio. Figure T2. Adjusted RRs and 95% CIs for the associations between demographic, anthropometric, substance use, and clinical risk factors and inadequate GWG (1-stage model) using the lower limit of the IOM recommendations to calculate expected GWG (*n =* 79,948). Circles represent RRs and bars represent 95% CIs. BMI, body mass index; CI, confidence interval; cm, centimeter; GWG, gestational weight gain; HIV, human immunodeficiency virus; MUAC, mid-upper arm circumference; RR, risk ratio. Figure U1. Unadjusted RRs and 95% CIs for the associations between demographic, anthropometric, substance use, and clinical risk factors and excessive GWG (1-stage model) using the lower limit of the IOM recommendations to calculate expected GWG (*n =* 79,948). Circles represent RRs and bars represent 95% CIs. BMI, body mass index; CI, confidence interval; cm, centimeter; GWG, gestational weight gain; HIV, human immunodeficiency virus; MUAC, mid-upper arm circumference; RR, risk ratio. Figure U2. Adjusted RRs and 95% CIs for the associations between demographic, anthropometric, substance use, and clinical risk factors and excessive GWG (1-stage model) using the lower limit of the IOM recommendations to calculate expected GWG (*n =* 79,948). Circles represent RRs and bars represent 95% CIs. BMI, body mass index; CI, confidence interval; cm, centimeter; GWG, gestational weight gain; HIV, human immunodeficiency virus; MUAC, mid-upper arm circumference; RR, risk ratio. Figure V1. Unadjusted RRs and 95% CIs for the associations between demographic, anthropometric, substance use, and clinical risk factors and severely inadequate GWG (2-stage model) using an Asia-specific BMI cutoff to define overweight/obesity for Asian participants (*n =* 138,286). Circles represent RRs and bars represent 95% CIs. BMI, body mass index; CI, confidence interval; cm, centimeter; GWG, gestational weight gain; HIV, human immunodeficiency virus; MUAC, mid-upper arm circumference; RR, risk ratio. Figure V2. Adjusted RRs and 95% CIs for the associations between demographic, anthropometric, substance use, and clinical risk factors and severely inadequate (2-stage model) using an Asia-specific BMI cutoff to define overweight/obesity for Asian participants (*n =* 138,286). Circles represent RRs and bars represent 95% CIs. BMI, body mass index; CI, confidence interval; cm, centimeter; GWG, gestational weight gain; HIV, human immunodeficiency virus; MUAC, mid-upper arm circumference; RR, risk ratio. Figure W1. Unadjusted RRs and 95% CIs for the associations between demographic, anthropometric, substance use, and clinical risk factors and inadequate GWG (2-stage model) using an Asia-specific BMI cutoff to define overweight/obesity for Asian participants (*n =* 138,286). Circles represent RRs and bars represent 95% CIs. BMI, body mass index; CI, confidence interval; cm, centimeter; GWG, gestational weight gain; HIV, human immunodeficiency virus; MUAC, mid-upper arm circumference; RR, risk ratio. Figure W2. Adjusted RRs and 95% CIs for the associations between demographic, anthropometric, substance use, and clinical risk factors and inadequate GWG (2-stage model) using an Asia-specific BMI cutoff to define overweight/obesity for Asian participants (*n =* 138,286). Circles represent RRs and bars represent 95% CIs. BMI, body mass index; CI, confidence interval; cm, centimeter; GWG, gestational weight gain; HIV, human immunodeficiency virus; MUAC, mid-upper arm circumference; RR, risk ratio. Figure X1. Unadjusted RRs and 95% CIs for the associations between demographic, anthropometric, substance use, and clinical risk factors and excessive GWG (2-stage model) using an Asia-specific BMI cutoff to define overweight/obesity for Asian participants (*n =* 138,286). Circles represent RRs and bars represent 95% CIs. BMI, body mass index; CI, confidence interval; cm, centimeter; GWG, gestational weight gain; HIV, human immunodeficiency virus; MUAC, mid-upper arm circumference; RR, risk ratio. Figure X2. Adjusted RRs and 95% CIs for the associations between demographic, anthropometric, substance use, and clinical risk factors and excessive GWG (2-stage model) using an Asia-specific BMI cutoff to define overweight/obesity for Asian participants (*n =* 138,286). Circles represent RRs and bars represent 95% CIs. BMI, body mass index; CI, confidence interval; cm, centimeter; GWG, gestational weight gain; HIV, human immunodeficiency virus; MUAC, mid-upper arm circumference; RR, risk ratio. Figure Y1. Unadjusted RRs and 95% CIs for the associations between demographic, anthropometric, substance use, and clinical risk factors and severely inadequate GWG (2-stage model) using those with adequate GWG as the reference category (*n =* 138,286). Circles represent RRs and bars represent 95% CIs. BMI, body mass index; CI, confidence interval; cm, centimeter; GWG, gestational weight gain; HIV, human immunodeficiency virus; MUAC, mid-upper arm circumference; RR, risk ratio. Figure Y2. Adjusted RRs and 95% CIs for the associations between demographic, anthropometric, substance use, and clinical risk factors and severely inadequate GWG (2-stage model) using those with adequate GWG as the reference category (*n =* 138,286). Circles represent RRs and bars represent 95% CIs. BMI, body mass index; CI, confidence interval; cm, centimeter; GWG, gestational weight gain; HIV, human immunodeficiency virus; MUAC, mid-upper arm circumference; RR, risk ratio. Figure Z1. Unadjusted RRs and 95% CIs for the associations between demographic, anthropometric, substance use, and clinical risk factors and inadequate GWG (2-stage model) using those with adequate GWG as the reference category (*n =* 138,286). Circles represent RRs and bars represent 95% CIs. BMI, body mass index; CI, confidence interval; cm, centimeter; GWG, gestational weight gain; HIV, human immunodeficiency virus; MUAC, mid-upper arm circumference; RR, risk ratio. Figure Z2. Adjusted RRs and 95% CIs for the associations between demographic, anthropometric, substance use, and clinical risk factors and inadequate GWG (2-stage model) using those with adequate GWG as the reference category (*n =* 138,286). Circles represent RRs and bars represent 95% CIs. BMI, body mass index; CI, confidence interval; cm, centimeter; GWG, gestational weight gain; HIV, human immunodeficiency virus; MUAC, mid-upper arm circumference; RR, risk ratio. Figure AA1. Unadjusted RRs and 95% CIs for the associations between demographic, anthropometric, substance use, and clinical risk factors and excessive GWG (2-stage model) using those with adequate GWG as the reference category (*n =* 138,286). Circles represent RRs and bars represent 95% CIs. BMI, body mass index; CI, confidence interval; cm, centimeter; GWG, gestational weight gain; HIV, human immunodeficiency virus; MUAC, mid-upper arm circumference; RR, risk ratio. Figure AA2. Adjusted RRs and 95% CIs for the associations between demographic, anthropometric, substance use, and clinical risk factors and excessive GWG (2-stage model) using those with adequate GWG as the reference category (*n =* 138,286). Circles represent RRs and bars represent 95% CIs. BMI, body mass index; CI, confidence interval; cm, centimeter; GWG, gestational weight gain; HIV, human immunodeficiency virus; MUAC, mid-upper arm circumference; RR, risk ratio. Figure BB1. Unadjusted RRs and 95% CIs for the associations between demographic, anthropometric, substance use, and clinical risk factors and severely inadequate GWG (1-stage model) among those who did not receive randomized interventions (*n =* 38,741). Circles represent RRs and bars represent 95% CIs. BMI, body mass index; CI, confidence interval; cm, centimeter; GWG, gestational weight gain; HIV, human immunodeficiency virus; MUAC, mid-upper arm circumference; RR, risk ratio. Figure BB2. Adjusted RRs and 95% CIs for the associations between demographic, anthropometric, substance use, and clinical risk factors and severely inadequate GWG (1-stage model) among those who did not receive randomized interventions (*n =* 38,741). Circles represent RRs and bars represent 95% CIs. BMI, body mass index; CI, confidence interval; cm, centimeter; GWG, gestational weight gain; HIV, human immunodeficiency virus; MUAC, mid-upper arm circumference; RR, risk ratio. Figure CC1. Unadjusted RRs and 95% CIs for the associations between demographic, anthropometric, substance use, and clinical risk factors and inadequate GWG (1-stage model) among those who did not receive randomized interventions (*n =* 38,741). Circles represent RRs and bars represent 95% CIs. BMI, body mass index; CI, confidence interval; cm, centimeter; GWG, gestational weight gain; HIV, human immunodeficiency virus; MUAC, mid-upper arm circumference; RR, risk ratio. Figure CC2 in S1 Appendix. Adjusted RRs and 95% CIs for the associations between demographic, anthropometric, substance use, and clinical risk factors and inadequate GWG (1-stage model) among those who did not receive randomized interventions (*n =* 38,741). Circles represent RRs and bars represent 95% CIs. BMI, body mass index; CI, confidence interval; cm, centimeter; GWG, gestational weight gain; HIV, human immunodeficiency virus; MUAC, mid-upper arm circumference; RR, risk ratio. Figure DD1. Unadjusted RRs and 95% CIs for the associations between demographic, anthropometric, substance use, and clinical risk factors and excessive GWG (1-stage model) among those who did not receive randomized interventions (*n =* 38,741). Circles represent RRs and bars represent 95% CIs. BMI, body mass index; CI, confidence interval; cm, centimeter; GWG, gestational weight gain; HIV, human immunodeficiency virus; MUAC, mid-upper arm circumference; RR, risk ratio. Figure DD2. Adjusted RRs and 95% CIs for the associations between demographic, anthropometric, substance use, and clinical risk factors and excessive GWG (1-stage model) among those who did not receive randomized interventions (*n =* 38,741). Circles represent RRs and bars represent 95% CIs. BMI, body mass index; CI, confidence interval; cm, centimeter; GWG, gestational weight gain; HIV, human immunodeficiency virus; MUAC, mid-upper arm circumference; RR, risk ratio.
(DOCX)

## Abbreviations

ART: antiretroviral therapy
BMI: body mass index
CI: confidence interval
GWG: gestational weight gain
HIC: high-income country
HIV: human immunodeficiency virus
IOM: Institute of Medicine
IQR: interquartile range
LGA: large for gestational age
LMIC: low- and middle-income country
MUAC: mid-upper arm circumference
RR: risk ratio
SD: standard deviation
SES: socioeconomic status
SGA: small for gestational age
