# Supplementary material for: Risk factors for inadequate and excessive gestational weight gain in 25 low- and middle-income countries: An individual-level participant meta-analysis
Source: PLoS Med. 2023 Jul 24;20(7):e1004236. doi: 10.1371/journal.pmed.1004236 (PMC10406332; doi:10.1371/journal.pmed.1004236)
Supplement: S1 Text — (DOCX) [file pmed.1004236.s001.docx]

**Members of the GWG Pooling Project Consortium**

1. Ajibola Ibraheem Abioye, Department of Global Health and Population, Harvard T.H. Chan School of Public Health, Boston, MA, USA
2. Seth Adu-Afarwuah, Department of Nutrition and Food Science, University of Ghana, Legon, Accra, Ghana
3. Hasmot Ali, The JiVitA Maternal & Child Health and Nutrition Research Institute, Rangpur, Bangladesh
4. Huseini Wiisibie Alidu, Department of Medical Laboratory Sciences, School of Allied Health Sciences, University of Health and Allied Sciences, PMB 31, Ho Ghana
5. Joao Guilherme Alves, Instituto de Medicina Integral Prof. Fernando Figueira (IMIP)
6. Carla Adriane Leal de Araújo, Instituto de Medicina Integral Prof. Fernando Figueira (IMIP)
7. Shams Arifeen, International Centre for Diarrheal Disease Research, Dhaka, Bangladesh
8. Rinaldo Artes, INSPER - Instituto de Ensino e Pesquisa, Sao Paulo, Brazil
9. Per Ashorn, Center for Child, Adolescent and Maternal Health Research, Faculty of Medicine and Health Technology, Tampere University and Tampere University Hospital, Tampere, Finland
10. Ulla Ashorn, Center for Child, Adolescent and Maternal Health Research, Faculty of Medicine and Health Technology, Tampere University and Tampere University Hospital, Tampere, Finland
11. Omolola Olukemi Ayoola, Lancashire Teaching Hospitals NHS Foundation Trust, Preston, UK
12. Fereidoun Azizi, Endocrine Research Center, Research Institute for Endocrine Sciences, Shahid Beheshti University of Medical Sciences
13. Ahmed Tijani Bawah, Department of Medical Laboratory Sciences, School of Allied Health Sciences, University of Health and Allied Sciences, PMB 31, Ho Ghana
14. Samira Behboudi-Gandevani, Nursing and Health Sciences, Nord University
15. Robin Bernstein, Department of Anthropology, University of Colorado, Boulder, Colorado, United States of America
16. Zulfiqar Bhutta, Centre for Global Child Health, Hospital for Sick Children, Toronto, Ontario, Canada & Institute for Global Health & Development, the Aga Khan University, Karachi, Pakistan
17. Valérie Briand, Institut de Recherche Pour le Développement (IRD), University of Bordeaux, Inserm, UMR 1219, 146 rue Léo-Saignat, 33076, Bordeaux Cedex, France
18. Elvira Beatriz Calvo, Former Head, Department of Nutrition, Mother & Child Health Direction. Ministry of Health, Argentina
19. Marly Augusto Cardoso, School of Public Health, University of Sao Paulo. Av. Dr. Arnaldo 715, 01246-904, Sao Paulo/SP, Brazil
20. Verena I Carrara, Shoklo Malaria Research Unit, Mahidol-Oxford, Tropical Medicine Research Unit, Mahidol University, Mae Sot, Thailand, Centre for Tropical Medicine and Global Health, University of Oxford, Oxford, UK, Institute of Global Health, Faculty of Medicine, University of Geneva, Geneva, Switzerland
21. Thaís Rangel Bousquet Carrilho, Nutritional Epidemiology Observatory, Josué de Castro Institute of Nutrition, Federal University of Rio de Janeiro; Av. Carlos Chagas Filho, 373 - Bloco J2 - sala 29, Rio de Janiero, Brazil

Cidade Universitária - Rio de Janeiro/RJ - 21941-902

1. Yue Cheng, Department of Nutrition and Food Safety Research, School of Public Health, Xi’an Jiaotong University Health Science Center, Xi’an, Shaanxi 710061, P.R. China,
2. Gabriela Chico-Barba, Nutrition and Bioprogramming Coordination, Instituto Nacional de Perinatología, Montes Urales 800, Lomas de Virreyes cp11000, Mexico City, Mexico
3. Peter Ellis Clayton, Faculty of Biology, Medicine & Health, University of Manchester, UK
4. Shalean M. Collins, Tulane University School of Public Health and Tropical Medicine, New Orleans, LA 70112
5. Anthony M Costello, UCL Institute for Global Health, 30 Guilford Street, London WC1N 1EH, UK
6. John Kennedy Cruickshank, St Thomas' & Guy's Hospitals, King’s College/ King’s Health Partners, London, UK
7. Christopher P. Duggan, Departments of Nutrition and Global Health and Population, Harvard T.H. Chan School of Public Health, Boston, MA, USA; Division of Gastroenterology, Hepatology and Nutrition, Boston Children’s Hospital, Harvard Medical School, Boston, Massachusetts
8. Pratibha Dwarkanath, Division of Nutrition, St. John's Research Institute, Bangalore India
9. Guadalupe Estrada-Gutierrez, Research Direction, Instituto Nacional de Perinatologia, Mexico City, Mexico
10. Frankie J. Fair, College of Health, Wellbeing and Life Sciences, Sheffield Hallam University, UK
11. Dayana Rodrigues Farias, Federal University of Rio de Janeiro/ Josué de Castro Institute of Nutrition, Av. Carlos Chagas Filho, 373 - Bloco J2 - sala 29, Cidade Universitária - Rio de Janeiro/RJ - 21941-590
12. Henrik Friis, Department of Nutrition, Exercise and Sports, University of Copenhagen, Rolighedsvej 26,1958 Frederiksberg C, Denmark
13. Alison D. Gernand, The Pennsylvania State University, Department of Nutritional Sciences; 110 Chandlee Laboratory, University Park, PA 16802
14. Shibani Ghosh, Friedman School of Nutrition Science and Policy, Tufts University, Boston, MA, USA
15. Exnevia Gomo, Faculty of Medicine and Health Sciences, University of Zimbabwe, Box A178 Avondale, Harare
16. Austrida Gondwe, UNC Project, Tidziwe Centre, 100 Mzimba Road, Kamuzu Central Hospital, P/Bag A-104, Lilongwe, Malawi
17. Rebecca Grais, Epicentre, 14-34 Avenue Jean Jaurès 75019 Paris, France
18. Ousmane Guindo, Epicentre Niger, Niamey, Niger
19. Lotta Hallamaa, Center for Child, Adolescent and Maternal Health Research, Faculty of Medicine and Health Technology, Tampere University, Tampere, Finland
20. K. Michael Hambidge, University of Colorado School of Medicine; Aurora, Colorado
21. Ahmar H Hashmi, Shoklo Malaria Research Unit, Mahidol-Oxford Tropical Medicine Research Unit, Mahidol University, Mae Sot, Thailand; Faculty of Medicine, Chiang Mai University, Chiang Mai, Thailand
22. Lieven Huybregts, Department of Food Technology, Safety and Health, Ghent University, Coupure links 653, 9000 Gent, Belgium; Poverty, Health and Nutrition Division, International Food Policy Research Institute, Washington, DC, USA
23. Romaina Iqbal, Department of Community Health Sciences, Aga Khan University, Karachi, Pakistan
24. José Roberto da Silva Junior, Instituto de Medicina Integral Prof. Fernando Figueira (IMIP)
25. Sheila Isanaka, Epicentre, 14-34 Avenue Jean Jaurès 75019 Paris, France; Harvard T.H. Chan School of Public Health, Departments of Nutrition and Global Health and Population, Boston, MA
26. Joanne Katz, Johns Hopkins Bloomberg School of Public Health, Baltimore, MD, USA
27. Subarna K. Khatry, Nepal Nutrition Intervention Project Sarlahi Project, Kathmandu, Nepal
28. Patrick Kolsteren, Department of Food Technology, Safety and Health, Ghent University, Coupure links 653, 9000 Gent, Belgium
29. Nancy Krebs, University of Colorado School of Medicine; Aurora, Colorado
30. Teija Kulmala, Pihlajalinna Group, Kehräsaari B, FIN-33200 Tampere, Finland
31. Pratap Kumar, Department of Reproductive Medicine and Surgery, Kasturba Medical College, Manipal Academy of Higher Education, Manipal-576104, Karnataka, India
32. Anura V. Kurpad, Department of Physiology, St. John's Medical College, Bangalore, India
33. Alain Labrique, Center for Human Nutrition, Dept of International Health, Bloomberg School of Public Health, Johns Hopkins University, Baltimore, MD, USA
34. Carl Lachat, Department of Food Technology, Safety and Health, Ghent University, Coupure links 653, 9000 Gent, Belgium
35. Anna Lartey, Department of Nutrition and Food Science, University of Ghana, Legon, Ghana
36. Jacqueline M Lauer, Department of Health Sciences, College of Health & Rehabilitation Sciences: Sargent College, Boston University, Boston, MA
37. Qian Li, **Department of Nutrition and Food Hygiene, Hubei Key Laboratory of Food Nutrition and Safety, MOE Key Laboratory of Environment and Health, School of Public Health, Tongji Medical College, Huazhong University of Science & Technology, Wuhan, China**
38. See Ling Loy, Department of Reproductive Medicine, KK Women’s and Children’s Hospital, 100 Bukit Timah Road, Singapore 229899, Singapore; Duke-NUS Medical School, 8 College Road, Singapore 169857, Singapore
39. Nur Indrawaty Lipoeto, Department of Nutrition, Andalas University, Padang, Indonesia
40. Laura Beatriz López, University of Buenos Aires. Faculty of Medicine, Nutrition School, Marcelo T de Alvear 2202. 4to Piso. C1121ABG CABA Argentina
41. Abdullah Al Mahmud, Nutrition and Clinical Services Division; icddr, b; 68 Shaheed Tajuddin Ahmed Sarani, Mohakhali, Dhaka 1212, Bangladesh
42. G. Arun Maiya, Department of Physiotherapy, Manipal College of Health Professions, Manipal Academy of Higher Education, Manipal-576104, Karnataka, India
43. Kenneth Maleta, School of Public Health and Family Medicine, University of Malawi, College of Medicine, Blantyre, Malawi, 1 Mahatma Gandhi Road, Private Bag 360, Blantyre 3, Malawi
44. Maíra Barreto Malta, School of Public Health, University of Sao Paulo. Av. Dr. Arnaldo 715, 01246-904, Sao Paulo/SP, Brazil
45. Dharma S Manandhar, Mother and Infant Research Activities (MIRA), GPO Box 921, Kathmandu, Nepal
46. Charles Mangani, School of Public Health and Family Medicine, University of Malawi, College of Medicine, Blantyre, Malawi, 1 Mahatma Gandhi Road, Private Bag 360, Blantyre 3, Malawi
47. Hugo Martínez-Rojano, Escuela Superior de Medicina del Instituto Politécnico Nacional, Plan de San Luis y Díaz Mirón s/n, Casco de Santo Tomas, Mexico City C. P. 11340, Mexico
48. Yves Martin-Prevel, MoISA, University of Montpellier, IRD, CIRAD, CIHEAM-IAMM, INRAE, Institut Agro, Montpellier, France
49. Reynaldo Martorell, Hubert Department of Global Health, Rollins School of Public Health, Emory University, Atlanta, GA
50. Susana L Matias, Department of Nutritional Sciences and Toxicology, University of California, Berkeley, CA
51. Elizabeth M. McClure, RTI International, Durham, NC, USA
52. Rose McGready, Shoklo Malaria Research Unit, Mahidol-Oxford Tropical Medicine Research Unit, Mahidol University, Mae Sot, Thailand; Centre for Tropical Medicine and Global Health, University of Oxford, Oxford, UK
53. Joshua D. Miller, Department of Nutrition, University of North Carolina at Chapel Hill, Chapel Hill, NC
54. Hamid Jan Jan Mohamed, Nutrition and Dietetics Programme, School of Health Sciences, Universiti Sains Malaysia, 16150 Kubang Kerian, Kelantan, Malaysia
55. Marhazlina Mohamad, School of Nutrition and Dietetics, Faculty of Health Sciences, Universiti Sultan Zainal Abidin (UniSZA), Gong Badak Campus, 21030 Kuala Nerus, Terengganu, Malaysia
56. Sophie Moore, Department of Women and Children’s Health, King’s College London, St Thomas’ Hospital, Westminster Bridge Road, London, SE1 7EH; MRC Unit The Gambia at the London School of Hygiene and Tropical Medicine, Fajara, The Gambia.
57. Paola Soledad Mosquera, School of Public Health, University of Sao Paulo. Av. Dr. Arnaldo 715, 01246-904, Sao Paulo/SP, Brazil
58. Malay Kanti Mridha, Center for Non-communicable Diseases and Nutrition, BRAC James P Grant School of Public Health, BRAC University, Dhaka, Bangladesh
59. Ferdinand M. Mugusi, Department of Internal Medicine, Muhimbili University of Health and Allied Sciences, Dar es Salaam, Tanzania
60. Cinthya Muñoz-Manrique, Nutrition and Bioprogramming Coordination, Instituto Nacional de Perinatología, Montes Urales 800, Lomas de Virreyes cp11000, Mexico City, Mexico
61. Salifu Nanga, Department of Basic Sciences, School of Basic and Biomedical Sciences, University of Health and Allied Sciences, PMB 31, Ho Ghana
62. Barnabas K. Natamba (deceased), Department of Research and Development, Ministry of Science Technology and Innovation, Kampala, Republic of Uganda
63. Maria Ome-Kaius, Papua New Guinea Institute of Medical Research, PO Box 60, Goroka, EHP, Papua New Guinea
64. David Osrin, Professor of Global Health, UCL Institute for Global Health, 30 Guilford Street, London WC1N 1EH, UK
65. Andrea B. Pembe, Department of Obstetrics and Gynaecology, Muhimbili University of Health and Allied Sciences, Dar es Salaam, Tanzania
66. Otilia Perichart-Perera, Nutrition and Bioprogramming Coordination, Instituto Nacional de Perinatología, Montes Urales 800, Lomas de Virreyes cp11000, Mexico City, Mexico
67. Zul Premji, Department of Parasitology/Medical Entomology, School of Public Health and Social Sciences, Muhimbili University of Health and Allied Sciences, Dar es Salaam, Tanzania
68. Andrew M. Prentice, MRC Unit The Gambia at London School of Hygiene & Tropical Medicine, Atlantic Boulevard, Fajara, PO Box 273, Banjul, The Gambia
69. Preetha Ramachandra, Department of Physiotherapy, Manipal College of Health Professions, Manipal Academy of Higher Education, Manipal-576104 Karnataka, India
70. Usha Ramakrishnan, Hubert Department of Global Health, Rollins School of Public Health, Emory University, Atlanta, GA
71. Juan Rivera, Director General, National Institute of Public Health, Cuernavaca, Morelos, Mexico
72. Dominique Roberfroid, Faculty of Medicine, University of Namur, Belgium, and Belgian Health Care Knowledge Centre (KCE), Brussels, Belgium
73. Ameyalli Rodríguez-Cano, Nutrition and Bioprogramming Coordination, Instituto Nacional de Perinatología, Montes Urales 800, Lomas de Virreyes cp11000, Mexico City, Mexico
74. Stephen J Rogerson, Department of Infectious Diseases, Doherty Institute, The University of Melbourne, Melbourne Australia
75. Patricia H C Rondó, University of Sao Paulo, School of Public Health, Nutrition Department. Avenida Dr Arnaldo 715, Sao Paulo, CEP-05409-010, Brazil
76. Daniel E. Roth, Department of Pediatrics, The Hospital for Sick Children and University of Toronto, 686 Bay Street, Toronto, ON M5G 0A4
77. Reyna Sámano, Nutrition and Bioprogramming Coordination, Instituto Nacional de Perinatología, Montes Urales 800, Lomas de Virreyes cp11000, Mexico City, Mexico.
78. Naomi M. Saville, University College London Institute for Global Health, 30 Guilford Street, London, WC1N 1EH
79. Bhim P. Shrestha, Health Research & Develop Forum (HRDF), Kathmandu, Nepal
80. Robin Shrestha, Friedman School of Nutrition Science and Policy, Tufts University, Boston, MA, USA
81. Hora Soltani, College of Health, Wellbeing and Life Sciences, Sheffield Hallam University, UK
82. Sajid Soofi, Centre of Excellence in Women and Child Health, the Aga Khan University, Karachi, Pakistan
83. Fahimeh Ramezani Tehrani, Reproductive Endocrinology Research Center, Research Institute for Endocrine Sciences, Shahid Beheshti University of Medical Sciences, Tehran, Iran
84. James Tielsch, George Washington Milken Institute School of Public Health
85. Holger W Unger, Menzies School of Health Research, Charles Darwin University, PO Box 41096, Casuarina NT 0811, Australia
86. Tinku Thomas, Department of Biostatistics, St. John's Medical College, Bangalore, India
87. Willy Urassa, Department of Microbiology and Immunology, Muhimbili University of Health and Allied Sciences, Dar es Salaam, Tanzania
88. Keith P. West, Jr., Center for Human Nutrition, Dept of Int’l Health, Bloomberg School of Public Health, Johns Hopkins University, Baltimore, MD, USA
89. Lee Wu, Center for Human Nutrition, Department of International Health, Bloomberg School of Public Health, Johns Hopkins University, Baltimore, MD, USA
90. Nianhong Yang, **Department of Nutrition and Food Hygiene, Hubei Key Laboratory of Food Nutrition and Safety, MOE Key Laboratory of Environment and Health, School of Public Health, Tongji Medical College, Huazhong University of Science & Technology, Wuhan, China**
91. Sera L. Young, Department of Anthropology, Institute for Policy Research, Northwestern University, Evanston, IL 60208
92. Emmanuel Yovo, Institut de Recherche Clinique du Bénin (IRCB), Abomey-Calavi, Benin
93. Lingxia Zeng, Department of Epidemiology and Biostatistics, School of Public Health, Xi’an Jiaotong University Health Science Center, Xi’an, Shaanxi 710061, P.R. China
94. Chunrong Zhong, Department of Nutrition and Food Hygiene, Hubei Key Laboratory of Food Nutrition and Safety, School of Public Health, Tongji Medical College, Huazhong University of Science and Technology, Wuhan, 430030, Hubei, China
95. Zhonghai Zhu, Department of Epidemiology and Biostatistics, School of Public Health, Xi’an Jiaotong University Health Science Center, Xi’an, Shaanxi 710061, P.R. China
