# Supplementary material for: Risk factors for inadequate and excessive gestational weight gain in 25 low- and middle-income countries: An individual-level participant meta-analysis
Source: PLoS Med. 2023 Jul 24;20(7):e1004236. doi: 10.1371/journal.pmed.1004236 (PMC10406332; doi:10.1371/journal.pmed.1004236)
Supplement: S1 Appendix — Table A. Systematic search strategy. Table B. Interventions received in trials included in pooled analyses. Table C. Overall participant characteristics. Figure A1. Unadjusted RRs and 95% CIs for the associations between demographic, anthropometric, substance use, and clinical risk factors and severely inadequate GWG (1-stage model, n = 79,948). Circles represent RRs and bars represent 95% CIs. BMI, body mass index; CI, confidence interval; cm, centimeter; GWG, gestational weight gain; HIV, human immunodeficiency virus; MUAC, mid-upper arm circumference; RR, risk ratio. Figure A2. Adjusted RRs and 95% CIs for the associations between demographic, anthropometric, substance use, and clinical risk factors and severely inadequate GWG (1-stage model, n = 79,948). Circles represent RRs and bars represent 95% CIs. BMI, body mass index; CI, confidence interval; cm, centimeter; GWG, gestational weight gain; HIV, human immunodeficiency virus; MUAC, mid-upper arm circumference; RR, risk ratio. Figure B1. Unadjusted RRs and 95% CIs for the associations between demographic, anthropometric, substance use, and clinical risk factors and inadequate GWG (1-stage model, n = 79,948). Circles represent RRs and bars represent 95% CIs. BMI, body mass index; CI, confidence interval; cm, centimeter; GWG, gestational weight gain; HIV, human immunodeficiency virus; MUAC, mid-upper arm circumference; RR, risk ratio. Figure B2. Adjusted RRs and 95% CIs for the associations between demographic, anthropometric, substance use, and clinical risk factors and inadequate GWG (1-stage model, n = 79,948). Circles represent RRs and bars represent 95% CIs. BMI, body mass index; CI, confidence interval; cm, centimeter; GWG, gestational weight gain; HIV, human immunodeficiency virus; MUAC, mid-upper arm circumference; RR, risk ratio. Figure C1. Unadjusted RRs and 95% CIs for the associations between demographic, anthropometric, substance use, and clinical risk factors and excessive GWG (1-stage model, n = 7 [file pmed.1004236.s004.docx]

**Risk factors for inadequate and excessive gestational weight gain in 25 low- and middle-income countries: an individual-level participant meta-analysis**

**Supplementary Material**

| **Table A in S1 Appendix.** Systematic search strategy | |
| --- | --- |
| **Concept** | **Search Terms** |
| (1) Clinical trials | (clinical[tiab] AND trial[tiab]) OR "clinical trials as topic"[mesh] OR "clinical trial"[pt] OR random*[tiab] OR "random allocation"[mesh] OR "therapeutic use"[sh] OR “controlled trial”[tiab] OR “interventional study”[tiab] OR “single-arm trial”[tiab] OR “clinical study”[tiab] OR “Phase II trial”[tiab] OR “Phase II study”[tiab] OR “Phase I/II trial”[tiab] OR “Phase I/II study”[tiab] |
| (2) Cohort studies | “Cohort Studies”[mesh] OR cohort[tiab] or prospective[tiab] or longitudinal[tiab] |
| (3) Weight | "Body Weight"[Mesh] OR "Weight"[tiab] OR "Body Mass Index"[tiab] OR "Body Mass Index"[Mesh] OR obesity[Mesh] OR obesity[tiab] OR underweight[tiab] OR undernutrition[tiab] OR “weight gain”[tiab] OR “weight change”[tiab] OR “overweight”[tiab] OR “normal weight”[tiab] OR anthropometr*[tiab] |
| (4) Pregnancy | "Pregnancy"[Mesh] OR Pregnanc*[tiab] OR Pregnant[tiab] OR prenatal[tiab] or gestation*[tiab] or antenatal[tiab] |
| (5) LMICs | ("Developing Countries"[mesh] OR developing countr*[tiab] OR developing nation*[tiab] OR less developed countr*[tiab] OR less developed nation*[tiab] OR third world nation*[tiab] OR third world countr*[tiab] OR under developed nation*[tiab] OR underdeveloped nation*[tiab] OR under developed countr*[tiab] OR underdeveloped nation*[tiab] OR middle income countr*[tiab] OR middle income nation*[tiab] OR low income countr*[tiab] OR low income nation*[tiab] OR poor countr*[tiab] OR poor nation*[tiab] OR lmic[tiab] OR lmics[tiab] OR "Africa"[mesh] OR "Asia"[mesh] OR "South America"[mesh] OR "Latin America"[mesh] OR "Central America"[mesh] OR africa[tiab] OR asia[tiab] OR south america*[tiab] OR latin america*[tiab] OR central america*[tiab] OR Afghanistan*[tiab] OR Albania*[tiab] OR Algeria*[tiab] OR Samoa*[tiab] OR Angola*[tiab] OR Armenia*[tiab] OR Azerbaijan*[tiab] OR Bangladesh*[tiab] OR Bengali[tiab] OR Belarus*[tiab] OR Belize[tiab] OR Benin[tiab] OR Bhutan*[tiab] OR Bolivia*[tiab] OR Bosnia*[tiab] OR Herzegovina*[tiab] OR Botswana*[tiab] OR Brazil*[tiab] OR Bulgaria*[tiab] OR Burkina Faso[tiab] OR Burundi*[tiab] OR Cabo Verd*[tiab] OR Cape Verd*[tiab] OR Cambodia*[tiab] OR Cameroon*[tiab] OR Central African*[tiab] OR Chad*[tiab] OR China[tiab] OR Chinese[tiab] OR Colombia*[tiab] OR Comoros[tiab] OR Congo[tiab] OR Cook Islands[tiab] OR Costa Rica*[tiab] OR Cote d'Ivoire[tiab] OR Ivory Coast[tiab] OR Cuba[tiab] OR Cuban[tiab] OR Djibouti[tiab] OR Dominica* [tiab] OR Ecuador[tiab] OR Egypt[tiab] OR El Salvador*[tiab] OR Eritrea*[tiab] OR Ethiopia*[tiab] OR Falkland Islands[tiab] OR Fiji*[tiab] OR Gabon*[tiab] OR Gambia*[tiab] OR Georgia*[tiab] OR Ghana*[tiab] OR Grenada*[tiab] OR Guadeloupe[tiab] OR Guatemala*[tiab] OR Guian*[tiab] OR Guinea*[tiab] OR Guyan*[tiab] OR Haiti*[tiab] OR Hondura*[tiab] OR India[tiab] OR Indian*[tiab] OR Indonesia*[tiab] OR Iran*[tiab] OR Iraq*[tiab] OR Jamaica*[tiab] OR Jordan*[tiab] OR Kazakh*[tiab] OR Kenya*[tiab] OR Kiribati[tiab] OR People's Republic of Korea[tiab] OR North Korea[tiab] OR Kosovo[tiab] OR Kosovar*[tiab] OR Kyrgyz*[tiab] OR Lao[tiab] OR Laos[tiab] OR Laotian*[tiab] OR Lebanon[tiab] OR Lebanes*[tiab] OR Lesotho[tiab] OR Liberia*[tiab] OR Libya*[tiab] OR Macedonia*[tiab] OR Madagascar*[tiab] OR Malawi*[tiab] OR Malvinas[tiab] OR Malaysia*[tiab] OR Maldives[tiab] OR Mali[tiab] OR Marshall Island*[tiab] OR Mauritania*[tiab] OR Mauriti*[tiab] OR Mayotte[tiab] OR Mexico[Mesh] OR Mexican*[tw] OR Micronesia*[tiab] OR Moldova*[tiab] OR Mongolia*[tiab] OR Montenegr*[tiab] OR Montserrat[tiab] OR Morocc*[tiab] OR Mozambique[tiab] OR Myanmar[tiab] OR Burmese*[tiab] OR Burma[tiab] OR Namibia*[tiab] OR Nauru[tiab] OR Nepal*[tiab] OR Netherlands Antilles[tiab] OR Nicaragua*[tiab] OR Niger*[tiab] OR Niue[tiab] OR Pakistan*[tiab] OR Paraguay*[tiab] OR Peru*[tiab] OR Philippin*[tiab] OR Pitcairn[tiab] OR Romania*[tiab] OR Rwanda*[tiab] OR Samoa*[tiab] OR Sao Tome[tiab] OR Principe[tiab] OR Senegal*[tiab] OR Serbia*[tiab] OR Sierra Leone*[tiab] OR Solomon Island*[tiab] OR Somalia* [tiab] OR South Africa*[tiab] OR Sri Lanka[tiab] OR St Helena[tiab] OR St Lucia[tiab] OR Saint Lucia[tiab] OR St Vincent[tiab] OR Saint Vincent[tiab] OR Grenad*[tiab] OR Sudan*[tiab] OR Suriname*[tiab] OR Swaziland*[tiab] OR Syria*[tiab] OR Tajik*[tiab] OR Tanzania*[tiab] OR Thai*[tiab] OR Timor*[tiab] OR Togo*[tiab] OR Tokelau[tiab] OR Tonga*[tiab] OR Tunisia*[tiab] OR Turkey[tiab] OR Turkish[tiab] OR Turkmen*[tiab] OR Tuvalu*[tiab] OR Uganda*[tiab] OR Ukrain*[tiab] OR Uzbeki*[tiab] OR Vanuatu*[tiab] OR Venezuela*[tiab] OR Vietnam*[tiab] OR Viet nam*[tiab] OR West Bank[tiab] OR Gaza*[tiab] OR Palestin*[tiab] OR Wallis and Futuna Island OR Yemen*[tiab] OR Zambia*[tiab] OR Zimbabw*[tiab] OR Western Sahara[tiab]) |
| (6) Micronutrients | Vitamins[Mesh] OR Micronutrients[Mesh] OR Dietary Supplements[Mesh] OR calcium[tiab] OR magnesium[tiab] OR phosphorus[tiab] OR potassium[tiab] OR boron[tiab] OR cobalt[tiab] OR chromium[tiab] OR copper[tiab] OR iodine[tiab] OR iron[tiab] OR manganese[tiab] OR molybdenum[tiab] OR selenium[tiab] OR zinc[tiab] OR vitamin*[tiab] OR thiamin*[tiab] or riboflavin[tiab] or niacin[tiab] OR “pantothenic acid”[tiab] OR pyridox*[tiab] OR biotin[tiab] OR folate[tiab] OR “folic acid”[tiab] OR cobalamin*[tiab] OR retinol[tiab] OR “ascorbic acid”[tiab] OR ergocalciferol[tiab] OR cholecaliferol[tiab] OR tocopherol*[tiab] OR tocotrienol*[tiab] OR phylloquinone[tiab] OR menaquinone[tiab] OR carotenoid*[tiab] or carotene[tiab] OR antioxidant*[tiab] OR “Balanced protein energy supplements”[tiab] OR “Lipid-based nutrient supplements”[tiab] OR “LNS”[tiab] |
| (7) Only human studies | NOT (Animals[Mesh] NOT (Animals[Mesh] AND Humans[Mesh])) |

| **Table B in S1 Appendix. Interventions received in trials included in pooled analyses** | | | |
| --- | --- | --- | --- |
| **Study acronym** | **Author, publication year** | **Country** | **Intervention**  **type** |
| NNIPS-3 | Christian 2003[1] | Nepal | Multiple micronutrients vs. Vitamin A + iron/folic acid vs. Vitamin A + zinc + iron/folic acid vs. Vitamin A + folic acid vs. Vitamin A |
| EU-MMN | Ramakrishnan 2003[2] | Mexico | Multiple micronutrients vs. iron |
| UZ-MatNutri | Friis 2004[3] | Zimbabwe | Multiple micronutrients vs. iron/folic acid |
| Mira-Janakpur | Osrin 2005[4] | Nepal | Multiple micronutrients vs. iron/folic acid |
| PNS | Fawzi 2007[5] | Tanzania | Multiple micronutrients vs. iron/folic acid |
| ICDDR-MINIMat | Tofail 2008[6] | Bangladesh | Early vs. usual supplementation with food and multiple micronutrients, 30 mg Iron + 400 mcg folate, or 60 mg Fe + 400 microg folate. |
| MISAME-1 | Roberfroid 2008[7] | Burkina Faso | United Nations International MPreparation (UNIMMAP) + sulphadoxine pyrimethamine vs. UNIMMAP + chloroquine vs. iron/folic acid + sulphadoxine pyrimethamine vs. iron/folic acid + chloroquine |
| XJU-RuralChina | Zeng 2008[8] | China | Multiple micronutrients vs. iron/folic acid vs. folic acid |
| AKU-MMN | Bhutta 2009[9] | Pakistan | Multiple micronutrient vs. iron/folic acid |
| MISAME-2 | Huybregts 2009[10] | Burkina Faso | UNIMMAP + sulphadoxine pyrimethamine (2 doses) vs. UNIMMAP + sulphadoxine pyrimethamine (3 doses) vs. lipid-based nutrients + sulphadoxine pyrimethamine (2 doses) vs. lipid-based nutrients sulphadoxine pyrimethamine (3 doses) |
| MRCG@LSHTM-ENID | Moore 2012[11] | The Gambia | Multiple micronutrients vs. protein-energy vs. multiple micronutrients + protein energy vs. iron/folic acid |
| JiVitA3 | West 2014[12] | Bangladesh | Multiple micronutrients vs. iron/folic acid |
| ILINS-DYAD-G | Adu-Afarwuah 2015[13] | Ghana | Multiple micronutrients vs. lipid-based nutrients vs. iron/folic acid |
| ILINS-DYAD-M | Ashorn 2015[14] | Malawi | Multiple micronutrients vs. lipid-based nutrients vs. iron/folic acid |
| MAL1 | Etheredge 2015[15] | Tanzania | Iron vs. placebo |
| JHU-MothersGift | Tielsch 2015[16] | Nepal | Flu vaccine vs. placebo |
| SPAZ-IPTp | Unger 2015[17] | Papua New Guinea | Sulphadoxine-pyrimethamine + azithromycin vs. sulphadoxine-pyrimethamine + chloroquine |
| AKU-VITD | Khan 2016[18] | Pakistan | Vitamin D vs. placebo |
| UC-RDNS | Matias 2016[19] | Bangladesh | Lipid based nutrient supplements vs. iron/folic acid |
| MAL2 | Darling 2017[20] | Tanzania | Vitamin A vs. Zinc vs. Vitamin A + zinc vs. placebo |
| XJU-Tibet | Kang 2017[21] | China | Multiple micronutrients vs. iron/folic acid |
| MDIG | Roth 2018[22] | Bangladesh | Vitamin D (4200 IU/week, 16,800 IU/week, or 28,000 IU/week) vs. placebo |
| UCL-LBWSAT | Saville 2018[23] | Nepal | Participatory learning and action group (PLA) vs. PLA + food vs. PLA + cash transfer vs. control (existing government programs) |
| LAIS | Hallamaa 2019[24] | Malawi | Sulphadoxine-pyrimethamine twice vs. sulphadoxine-pyrimethamine monthly vs. sulphadoxine-pyrimethamine + azithromycin |
| WomenFirst | Hambidge 2019[25] | Guatemala, India, and Pakistan | Maternal nutrition supplementation initiated preconception vs. maternal nutrition supplementation initatied at ~11 weeks gestation vs. no supplement |
| ROSE | Isanaka 2019[26] | Niger | Lipid based nutritent supplements vs. multiple micronutrients vs. iron/folic acid |
| SBUMS-GDM | Tehrani 2019[27] | Iran | Gestational diabetes screening methods |
| IMIP-BRAMAG | de Araújo 2020[28] | Brazil | Magnesium vs. placebo |

**Table B References:**

1. Christian P, Khatry SK, Katz J, Pradhan EK, LeClerq SC, Shrestha SR et al. Effects of alternative maternal micronutrient supplements on low birth weight in rural Nepal: double blind randomised community trial. BMJ. 2003;326(7389):571.
2. Ramakrishnan U, González-Cossío T, Neufeld LM, Rivera J, Martorell R. Multiple micronutrient supplementation during pregnancy does not lead to greater infant birth size than does iron-only supplementation: a randomized controlled trial in a semirural community in Mexico. Am J Clin Nutr. 2003;77(3):720-725
3. Friis H, Gomo E, Nyazema N, Ndhlovu P, Krarup H, Kaestel P, et al. Effect of multimicronutrient supplementation on gestational length and birth size: a randomized, placebo-controlled, double-blind effectiveness trial in Zimbabwe. Am J Clin Nutr. 2004;80(1):178-184.
4. Osrin D, Vaidya A, Shrestha Y, Baniya RB, Manandhar DS, Adhikari RK, et al. Effects of antenatal multiple micronutrient supplementation on birthweight and gestational duration in Nepal: double-blind, randomised controlled trial. Lancet. 2005 Mar 12-18;365(9463):955-62.
5. Fawzi WW, Msamanga GI, Urassa W, Hertzmark E, Petraro P, Willett WC, et al. Vitamins and perinatal outcomes among HIV-negative women in Tanzania. N Engl J Med. 2007;356(14):1423-1431.
6. Tofail F, Persson LA, El Arifeen S, Hamadani JD, Mehrin F, Ridout D, et al. Effects of prenatal food and micronutrient supplementation on infant development: a randomized trial from the Maternal and Infant Nutrition Interventions, Matlab (MINIMat) study. Am J Clin Nutr. 2008;87(3):704-711.
7. Roberfroid D, Huybregts L, Lanou H, Henry MC, Meda N, Menten J, et al. Effects of maternal multiple micronutrient supplementation on fetal growth: a double-blind randomized controlled trial in rural Burkina Faso. Am J Clin Nutr. 2008;88(5):1330-1340.
8. Zeng L, Dibley MJ, Cheng Y, Dang S, Chang S, Kong L, et al. Impact of micronutrient supplementation during pregnancy on birth weight, duration of gestation, and perinatal mortality in rural western China: double blind cluster randomised controlled trial. BMJ. 2008;337:a2001.
9. Bhutta ZA, Rizvi A, Raza F, Hotwani S, Zaidi S, Moazzam Hossain S, et al. A comparative evaluation of multiple micronutrient and iron-folic acid supplementation during pregnancy in Pakistan: impact on pregnancy outcomes. Food Nutr Bull. 2009;30(4 Suppl):S196-505.
10. Huybregts L, Roberfroid D, Lanou H, Menten J, Meda N, Van Camp J, et al. Prenatal food supplementation fortified with multiple micronutrients increases birth length: a randomized controlled trial in rural Burkina Faso. Am J Clin Nutr. 2009;90(6):1593-1600.
11. Moore SE, Fulford AJ, Darboe MK, Jobarteh ML, Jarjou LM, Prentice AM. A randomized trial to investigate the effects of pre-natal and infant nutritional supplementation on infant immune development in rural Gambia: the ENID trial: Early Nutrition and Immune Development. BMC Pregnancy Childbirth. 2012;12:107.
12. West KP Jr, Shamim AA, Mehra S, Labrique AB, Ali H, Shaikh S, et al. Effect of maternal multiple micronutrient vs iron-folic acid supplementation on infant mortality and adverse birth outcomes in rural Bangladesh: the JiVitA-3 randomized trial. JAMA. 2014;312(24):2649-2658
13. Adu-Afarwuah S, Lartey A, Okronipa H, Ashorn P, Zeilani M, Peerson JM, et al. Lipid-based nutrient supplement increases the birth size of infants of primiparous women in Ghana. Am J Clin Nutr. 2015;101(4):835-846.
14. Ashorn P, Alho L, Ashorn U, Cheung YB, Dewey KG, Harjunmaa U, L et al. The impact of lipid-based nutrient supplement provision to pregnant women on newborn size in rural Malawi: a randomized controlled trial. Am J Clin Nutr. 2015;101(2):387-397.
15. Etheredge AJ, Premji Z, Gunaratna NS, Abioye AI, Aboud S, Duggan C, et al. Iron Supplementation in Iron-Replete and Nonanemic Pregnant Women in Tanzania: A Randomized Clinical Trial. JAMA Pediatr. 2015;169(10):947-955.
16. Tielsch JM, Steinhoff M, Katz J, Englund JA, Kuypers J, Khatry SK, et al. Designs of two randomized, community-based trials to assess the impact of influenza immunization during pregnancy on respiratory illness among pregnant women and their infants and reproductive outcomes in rural Nepal. BMC Pregnancy Childbirth. 2015;15:40.
17. Unger HW, Ome-Kaius M, Wangnapi RA, Umbers AJ, Hanieh S, Suen CS, et al. Sulphadoxine-pyrimethamine plus azithromycin for the prevention of low birthweight in Papua New Guinea: a randomised controlled trial. BMC Med. 2015;13:9.
18. Khan FR, Ahmad T, Hussain R, Bhutta ZA. A randomized controlled trial of oral vitamin D supplementation in pregnancy to improve maternal periodontal health and birth weight. Journal of International Oral Health. 2016;8(6):657.
19. Matias SL, Mridha MK, Paul RR, Hussain S, Vosti SA, Arnold CD. Prenatal Lipid-Based Nutrient Supplements Affect Maternal Anthropometric Indicators Only in Certain Subgroups of Rural Bangladeshi Women. J Nutr. 2016;146(9):1775-1782.
20. Darling AM, Mugusi FM, Etheredge AJ, Gunaratna NS, Abioye AI, Aboud S, et al. Vitamin A and Zinc Supplementation Among Pregnant Women to Prevent Placental Malaria: A Randomized, Double-Blind, Placebo-Controlled Trial in Tanzania. Am J Trop Med Hyg. 2017;96(4):826-834.
21. Kang Y, Dang S, Zeng L, Wang D, Li Q, Wang J et al. Multi-micronutrient supplementation during pregnancy for prevention of maternal anaemia and adverse birth outcomes in a high-altitude area: a prospective cohort study in rural Tibet of China. Br J Nutr. 2017;118(6):431-440.
22. Roth DE, Morris SK, Zlotkin S, Gernand AD, Ahmed T, Shanta SS, et al. Vitamin D Supplementation in Pregnancy and Lactation and Infant Growth. N Engl J Med. 2018;379(6):535-546.
23. Saville NM, Shrestha BP, Style S, Harris-Fry H, Beard BJ, Sen A, et al. Impact on birth weight and child growth of Participatory Learning and Action women's groups with and without transfers of food or cash during pregnancy: Findings of the low birth weight South Asia cluster-randomised controlled trial (LBWSAT) in Nepal. PLoS One. 2018;13(5):e0194064.
24. Hallamaa L, Cheung YB, Luntamo M, Ashorn U, Kulmala T, Mangani C, et al. The impact of maternal antenatal treatment with two doses of azithromycin and monthly sulphadoxine-pyrimethamine on child weight, mid-upper arm circumference and head circumference: A randomized controlled trial. PLoS One. 2019;14(5):e0216536.
25. Hambidge KM, Westcott JE, Garcés A, Figueroa L, Goudar SS, Dhaded SM, et al. A multicountry randomized controlled trial of comprehensive maternal nutrition supplementation initiated before conception: the Women First trial. Am J Clin Nutr. 2019;109(2):457-469.1.
26. Isanaka S, Kodish SR, Mamaty AA, Guindo O, Zeilani M, Grais RF. Acceptability and utilization of a lipid-based nutrient supplement formulated for pregnant women in rural Niger: a multi-methods study. BMC Nutr. 2019;5:34.
27. Tehrani FR. Gulf Study Cooperative Research Group. Cost effectiveness of different screening strategies for gestational diabetes mellitus screening: study protocol of a randomized community non-inferiority trial. Diabetol Metab Syndr. 2019;11:106
28. de Araújo CAL, Ray JG, Figueiroa JN, Alves JG. BRAzil magnesium (BRAMAG) trial: a double-masked randomized clinical trial of oral magnesium supplementation in pregnancy. BMC Pregnancy Childbirth. 2020;20(1):234.

| **Table C in S1 Appendix.** Overall Participant Characteristics (n=138,286) | |
| --- | --- |
| **Characteristic** | **N (%)^1^** |
| Woman’s age |  |
| < 20 years | 40,044 (29.6) |
| 20 – 24 years | 23,420 (17.3) |
| 24 – 29 years | 36,799 (27.2) |
| 30 – 34 years | 22,047 (16.3) |
| ≥ 35 years | 13,073 (9.7) |
|  |  |
|  |  |
| Women’s educational level |  |
| 0 – 7 years | 49,019 (57.4) |
| 8 – 11 years | 21,649 (25.3) |
| ≥ 12 years | 14,777 (17.3) |
|  |  |
| Partner’s educational level |  |
| 0 – 7 years | 12,119 (50.6) |
| 8 – 11 years | 5,682 (23.7) |
| ≥ 12 years | 6,138 (25.6) |
|  |  |
| Woman’s occupation |  |
| Does not work outside home | 19,148 (62.1) |
| Agriculture/informal sector | 6,421 (20.8) |
| Formal sector | 5,291 (17.2) |
|  |  |
| Partner’s occupation |  |
| Does not work outside home | 854 (4.2) |
| Agriculture/informal sector | 13,256 (65.0) |
| Formal sector | 6,277 (30.8) |
|  |  |
| Married/cohabitating |  |
| No | 7,321 (6.4) |
| Yes | 106,278 (93.7) |
| Parity |  |
| 0 previous births | 35,450 (40.6) |
| 1 previous birth | 23,556 (27.1) |
| 2 previous births | 13,146 (15.1) |
| 3 previous births | 86,758 (7.) |
| ≥ 4 previous births | 8,092 (9.3) |
|  |  |
| HIV positive |  |
| No | 49,935 (98.4) |
| Yes | 800 (1.6) |
| Chronic hypertension |  |
| No | 58,680 (98.0) |
| Yes | 1,112 (2.0) |
| Woman’s Body Mass Index |  |
| < 18. 5 kg/m^2^ | 27,235 (19.7) |
| 18.5 - < 25 kg/m^2^ | 85,193 (61.6) |
| ≥ 25 kg/m^2^ | 25,858 (18.7) |
| Woman’s Mid-upper arm circumference |  |
| Underweight | 11,739 (32.4) |
| Adequate | 18,658 (51.4) |
| Overweight/obese | 5,863 (16.2) |
|  |  |
| Woman’s height |  |
| < 145 cm | 10,485 (7.6) |
| 145 - < 150 cm | 23,985 (17.3) |
| 150 - < 155 cm | 35,769 (25.9) |
| ≥ 155 cm | 68,051 (49.2) |
|  |  |
| Pre-pregnancy smoking |  |
| No | 50,634 (99.3) |
| Yes | 344 (0.7) |
| First-trimester smoking |  |
| No | 63,162 (96.3) |
| Yes | 2,451 (3.7) |
| Second-trimester smoking |  |
| No | 51,500 (99.4) |
| Yes | 300 (0.6) |
| Third-trimester smoking |  |
| No | 38,327 (98.2) |
| Yes | 703 (1.8) |
| First-trimester alcohol consumption |  |
| No | 21,624 (92.1) |
| Yes | 1,857 (7.7) |
| Second-trimester alcohol consumption |  |
| No | 19,414 (91.5) |
| Yes | 1,796 (8.5) |
| Third-trimester alcohol consumption |  |
| No | 10,669 (96.3) |
| Yes | 795 (6.9) |
| Any anemia during pregnancy |  |
| No | 19,811 (47.9) |
| Yes | 21,546 (52.1) |
| Any diarrhea during pregnancy |  |
| No | 5,324 (93.4) |
| Yes | 377 (6.6) |
| Any nausea during pregnancy |  |
| No | 4,508 (63.1) |
| Yes | 2,636 (36.9) |
| Any malaria during pregnancy |  |
| No | 16,750 (79.3) |
| Yes | 4,385 (20.8) |

^cm=centimeter, m=meter, HIV=Human immunodeficiency virus^

^1^Frequencies and percentages apply only to studies in which a characteristic was measured.

**Figure A1 in S1 Appendix.** Unadjusted Risk ratios and 95% confidence intervals for the associations between demongraphic, anthropometric, substance use, and clinical risk factors and severely inadequate GWG (1-stage model, n=79,948). Circles represent risk ratios and bars represent 95% confidence intervals.


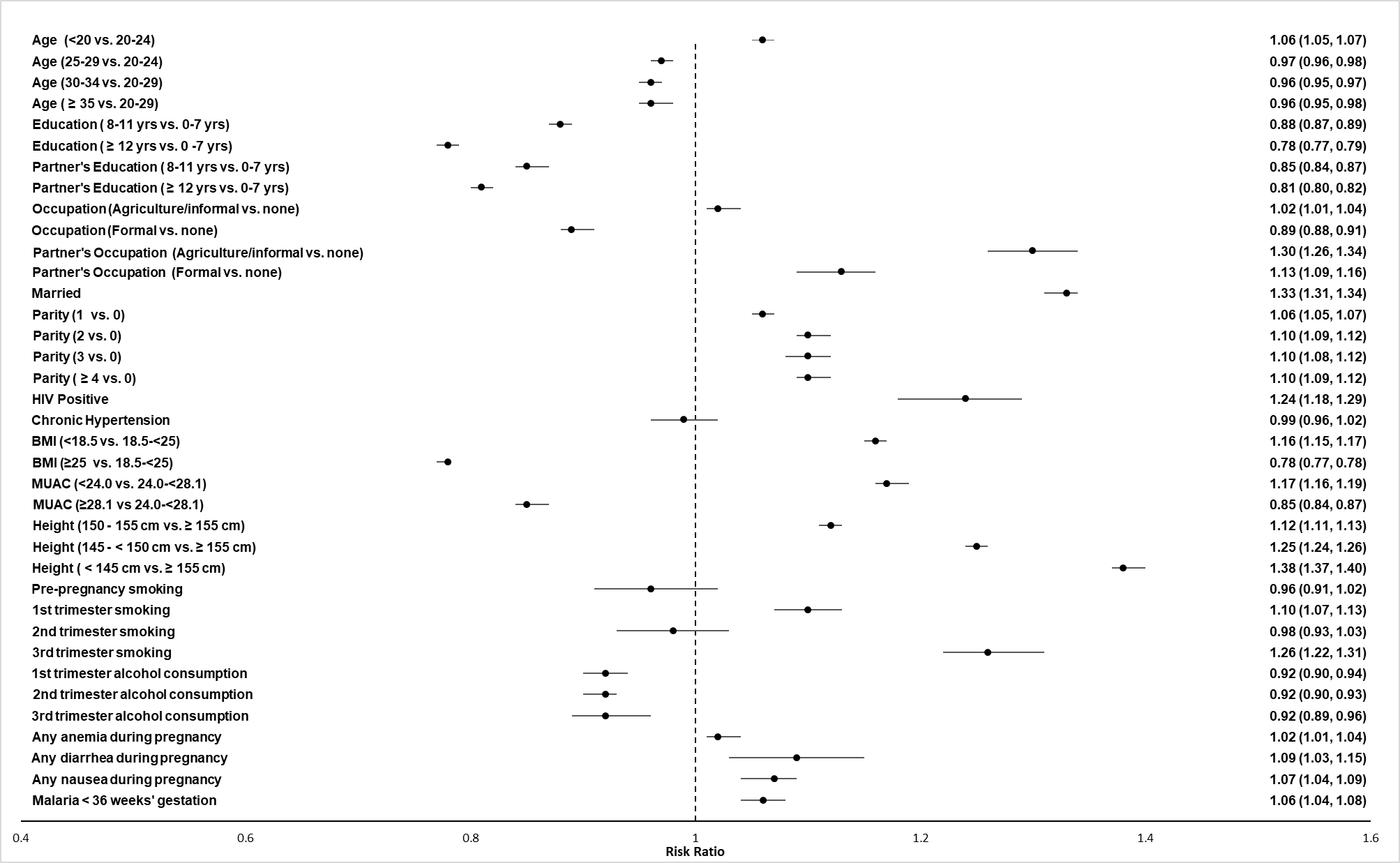


GWG=Gestational weight gain, BMI=body mass index, MUAC=mid-upper arm circumference, HIV=Human immunodeficiency virus, cm=centimeter

**Figure A2 in S1 Appendix.** Adjusted risk ratios and 95% confidence intervals for the associations between demongraphic, anthropometric, substance use, and clinical risk factors and severely inadequate GWG (1-stage model, n-79,948). Circles represent risk ratios and bars represent 95% confidence intervals.


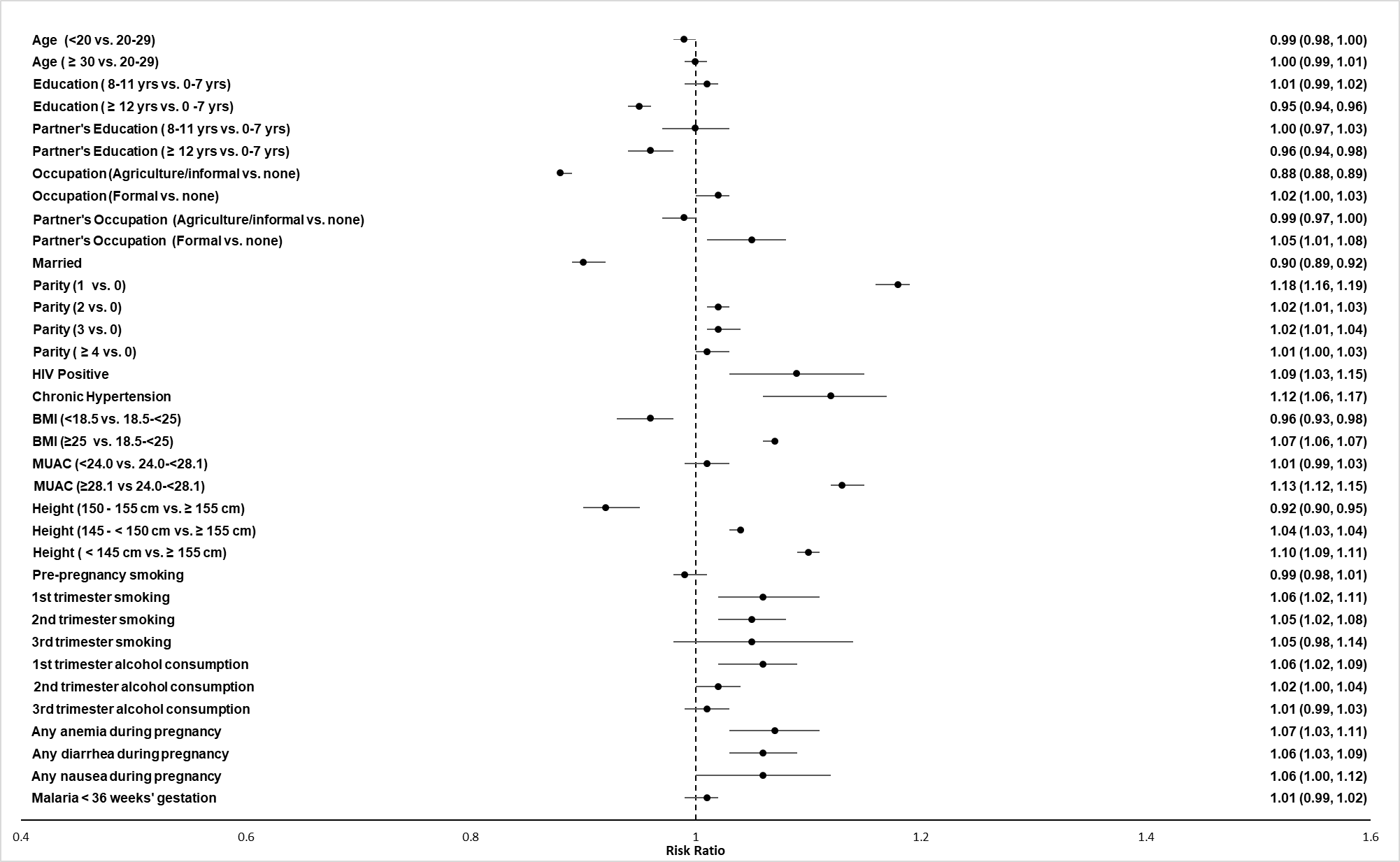


GWG=Gestational weight gain, BMI=body mass index, MUAC=mid-upper arm circumference, HIV=Human immunodeficiency virus, cm=centimeter

**Figure B1 in S1 Appendix.** Undjusted Risk ratios and 95% confidence intervals for the associations between demongraphic, anthropometric, substance use, and clinical risk factors and inadequate GWG (1-stage model, n=79,948). Circles represent risk ratios and bars represent 95% confidence intervals.


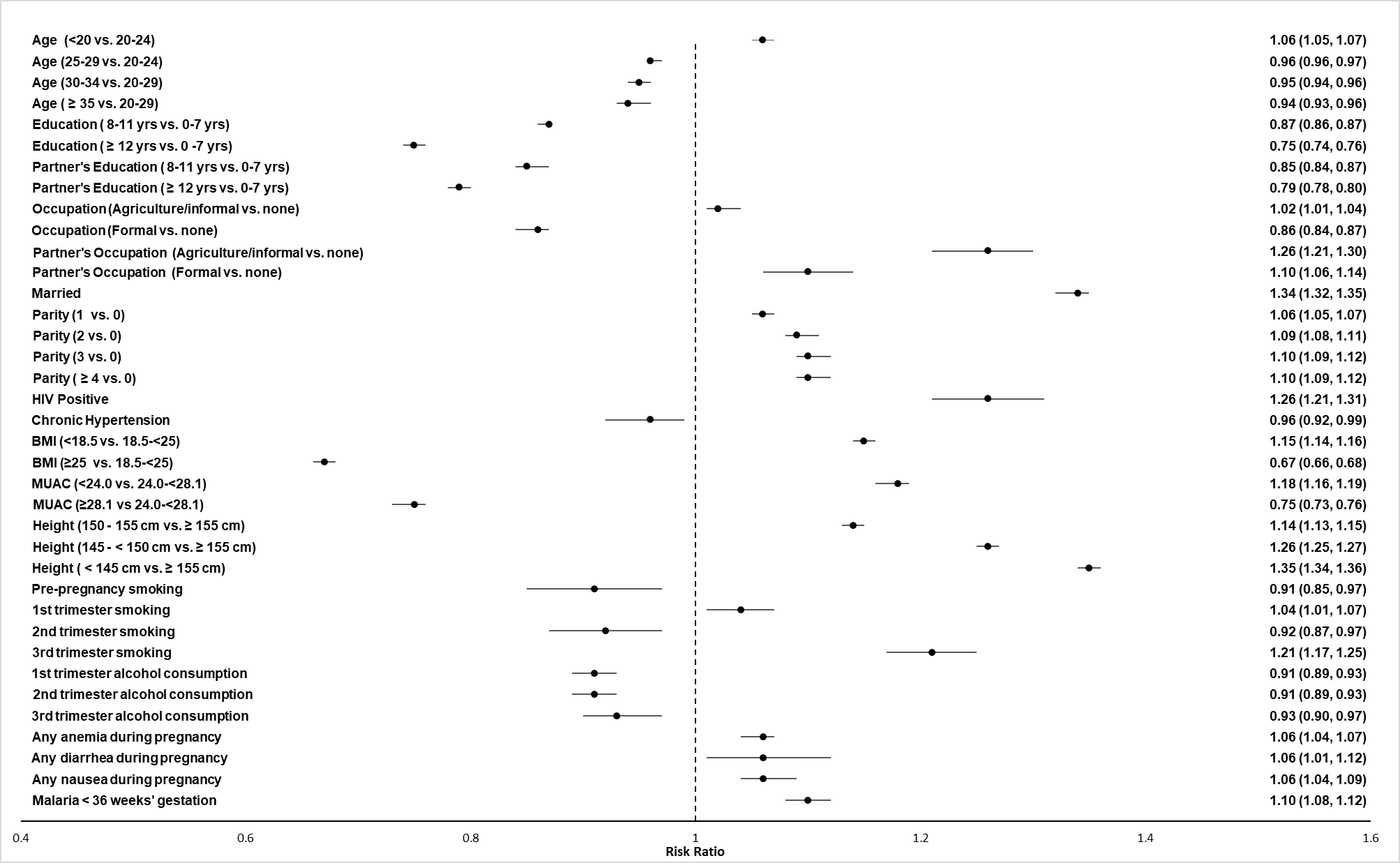


GWG=Gestational weight gain, BMI=body mass index, MUAC=mid-upper arm circumference, HIV=Human immunodeficiency virus, cm=centimeter

**Figure B2 in S1 Appendix.** Adjusted Risk ratios and 95% confidence intervals for the associations between demongraphic, anthropometric, substance use, and clinical risk factors and inadequate GWG (1-stage model, n=79,948). Circles represent risk ratios and bars represent 95% confidence intervals.


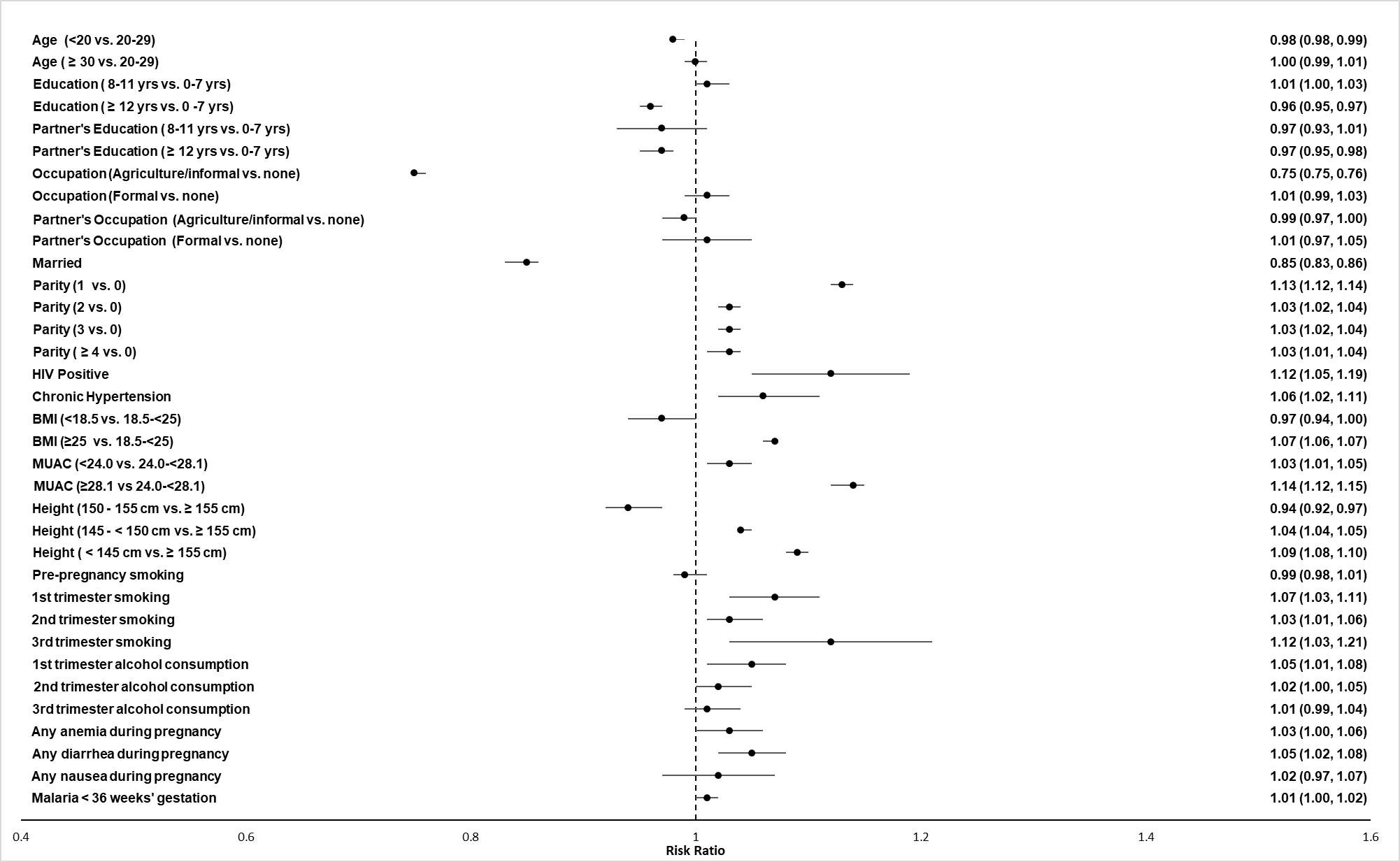


GWG=Gestational weight gain, BMI=body mass index, MUAC=mid-upper arm circumference, HIV=Human immunodeficiency virus, cm=centimeter

**Figure C1 in S1 Appendix.** Unadjusted risk ratios and 95% confidence intervals for the associations between demongraphic, anthropometric, substance use, and clinical risk factors and excessive GWG (1-stage model, n=79,948). Circles represent risk ratios and bars represent 95% confidence intervals


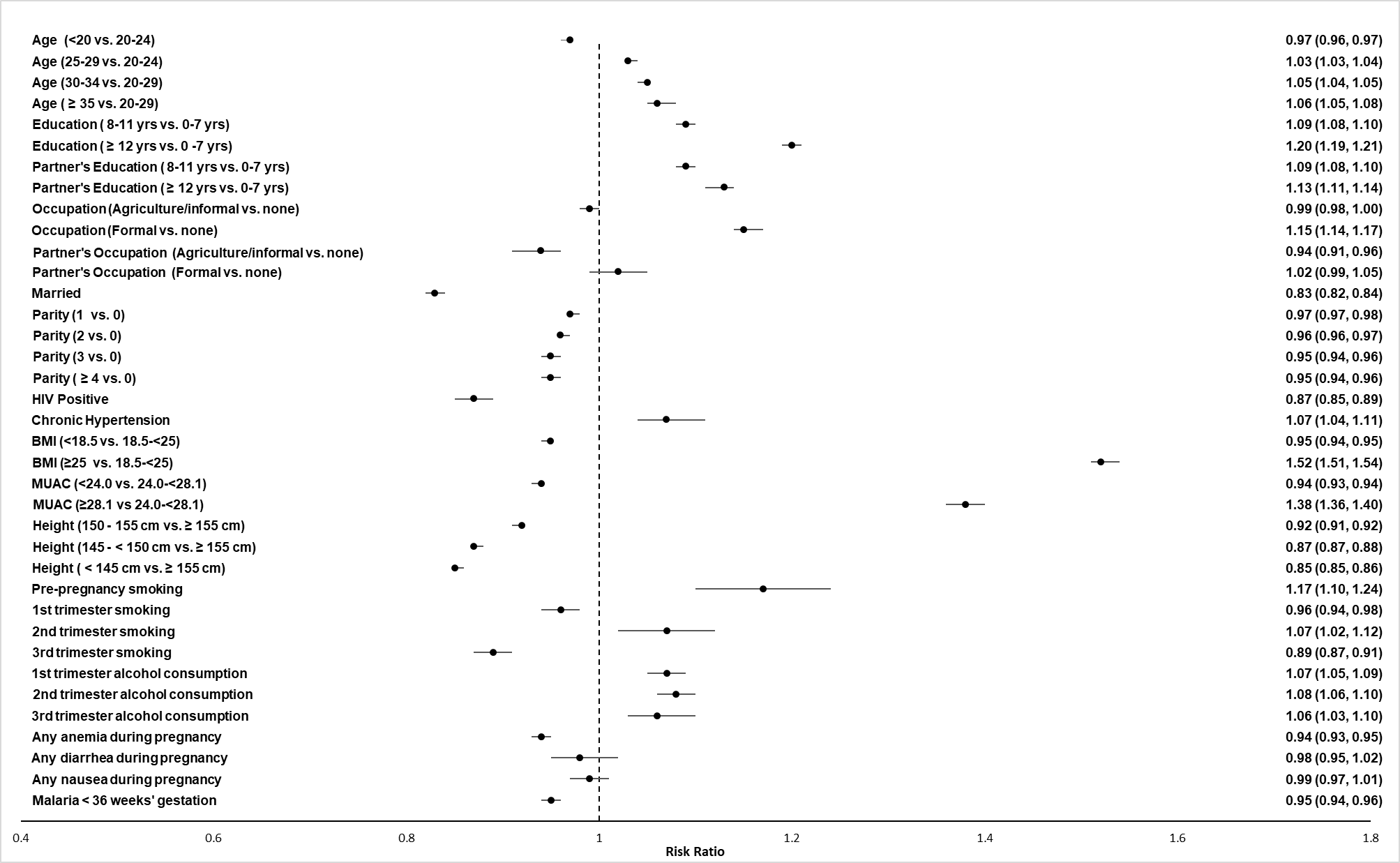


GWG=Gestational weight gain, BMI=body mass index, MUAC=mid-upper arm circumference, HIV=Human immunodeficiency virus, cm=centimeter

**Figure C2 in S1 Appendix.** Adjusted risk ratios and 95% confidence intervals for the associations between demongraphic, anthropometric, substance use, and clinical risk factors and excessive GWG (1-stage model, n=79,948). Circles represent risk ratios and bars represent 95% confidence intervals.


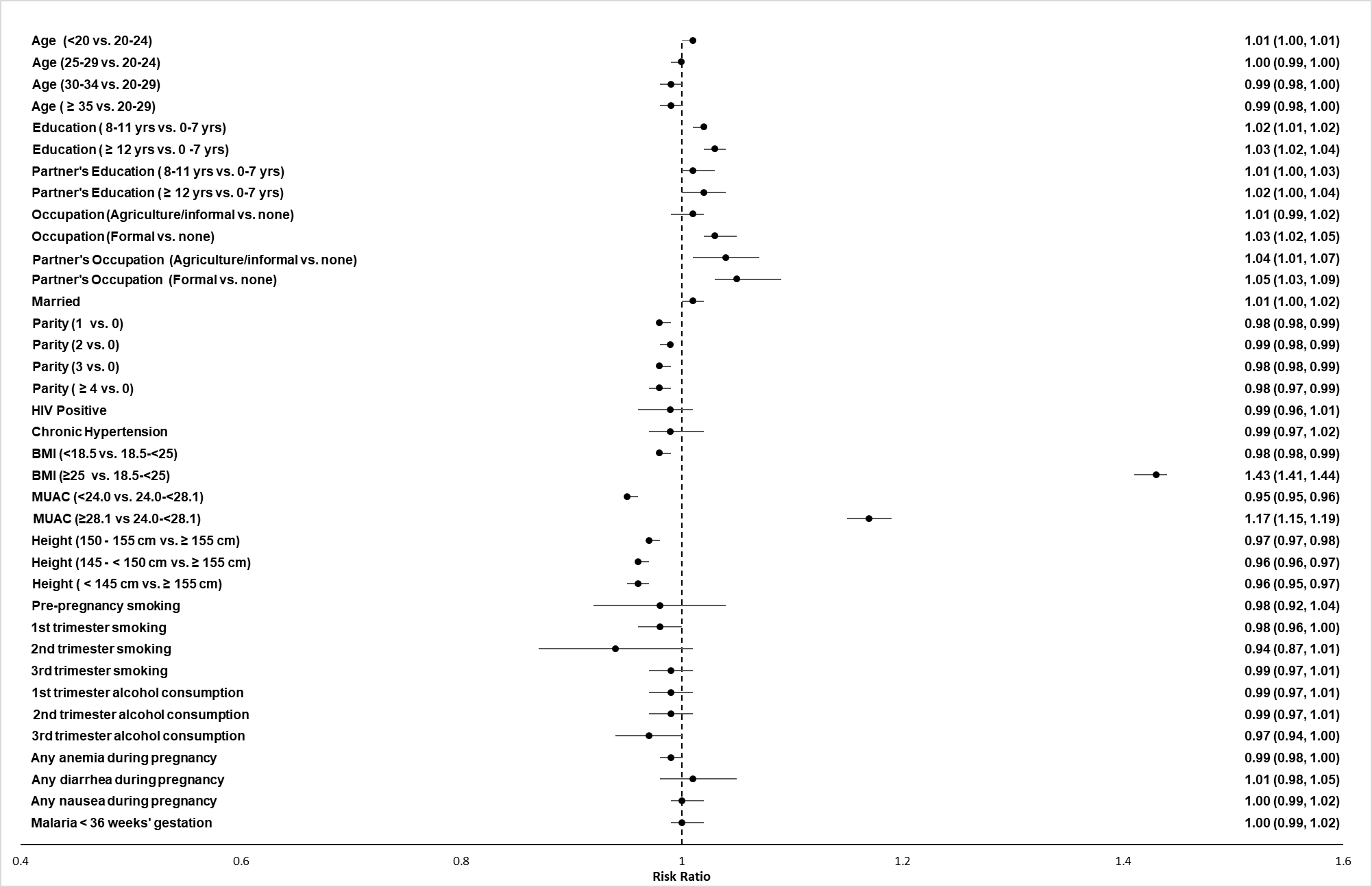
GWG=Gestational weight gain, BMI=body mass index, MUAC=mid-upper arm circumference, HIV=Human immunodeficiency virus, cm=centimeter

**Figure D1 in S1 Appendix.** Unadjusted risk ratios and 95% confidence intervals for the associations between demongraphic, anthropometric, and clinical risk factors and severely inadequate GWG (1-stage model) among participants with underweight (n=19,735). Circles represent risk ratios and bars represent 95% confidence intervals.


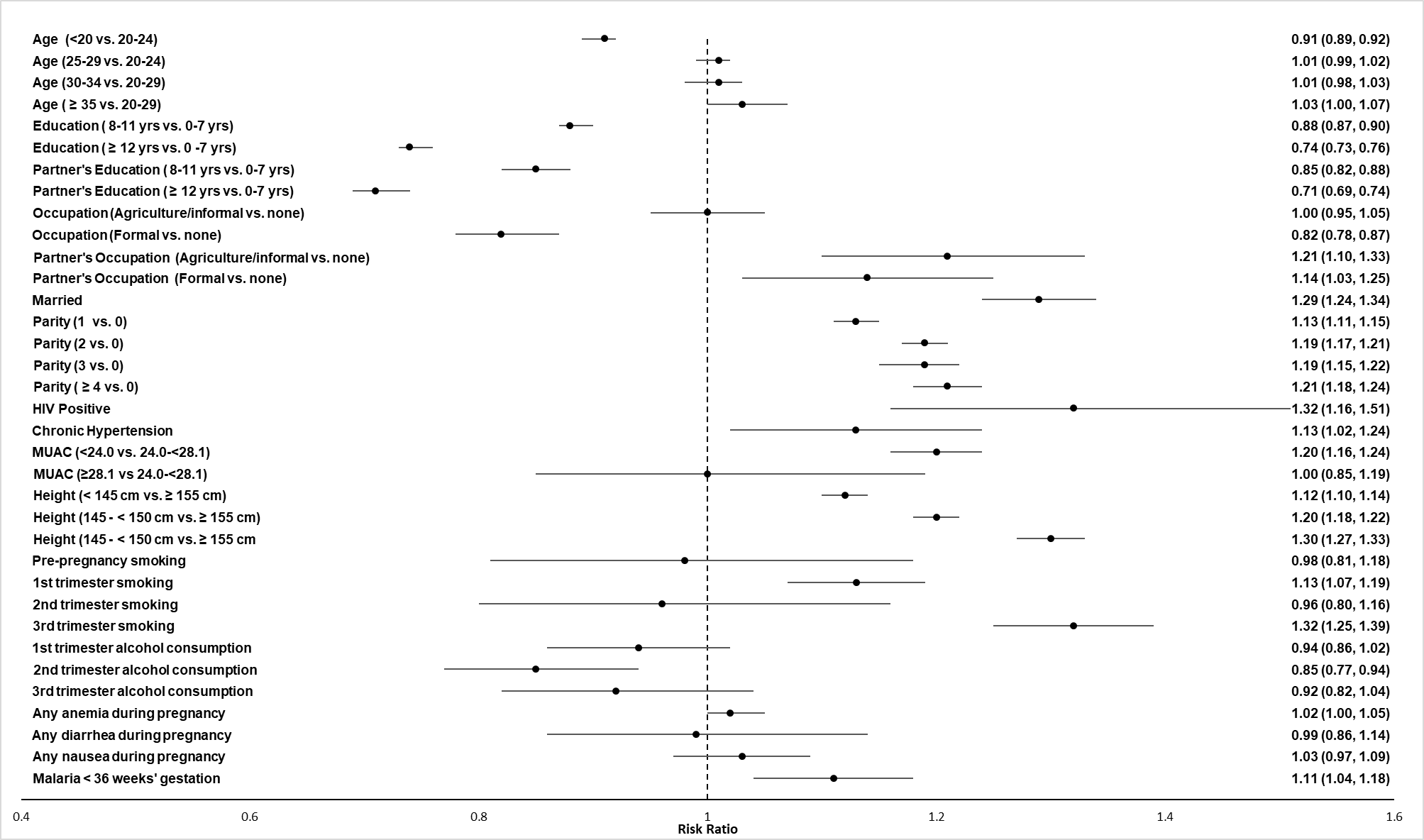


GWG=Gestational weight gain, BMI=body mass index, MUAC=mid-upper arm circumference, HIV=Human immunodeficiency virus, cm=centimeter

**Figure D2 in S1 Appendix.** Adjusted risk ratios and 95% confidence intervals for the associations between demongraphic, anthropometric, and clinical risk factors and severely inadequate GWG (1-stage model) among participants with underweight (n=19,735). Circles represent risk ratios and bars represent 95% confidence intervals


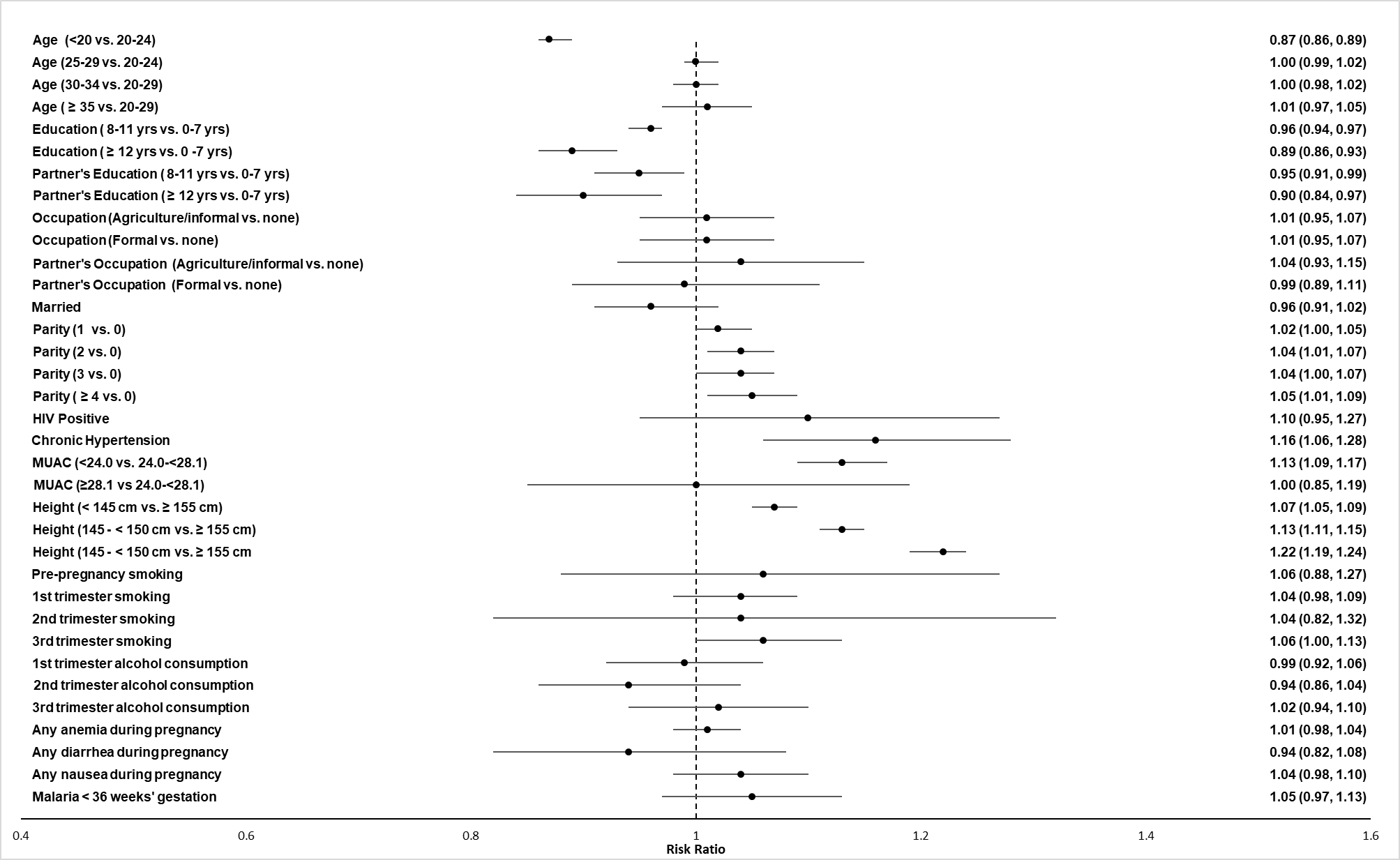


GWG=Gestational weight gain, BMI=body mass index, MUAC=mid-upper arm circumference, HIV=Human immunodeficiency virus, cm=centimeter

**Figure E1 in S1 Appendix.** Unadjusted risk ratios and 95% confidence intervals for the associations between demongraphic, anthropometric, and clinical risk factors and inadequate GWG (1-stage model) among participants with underweight (n=19,735). Circles represent risk ratios and bars represent 95% confidence intervals.


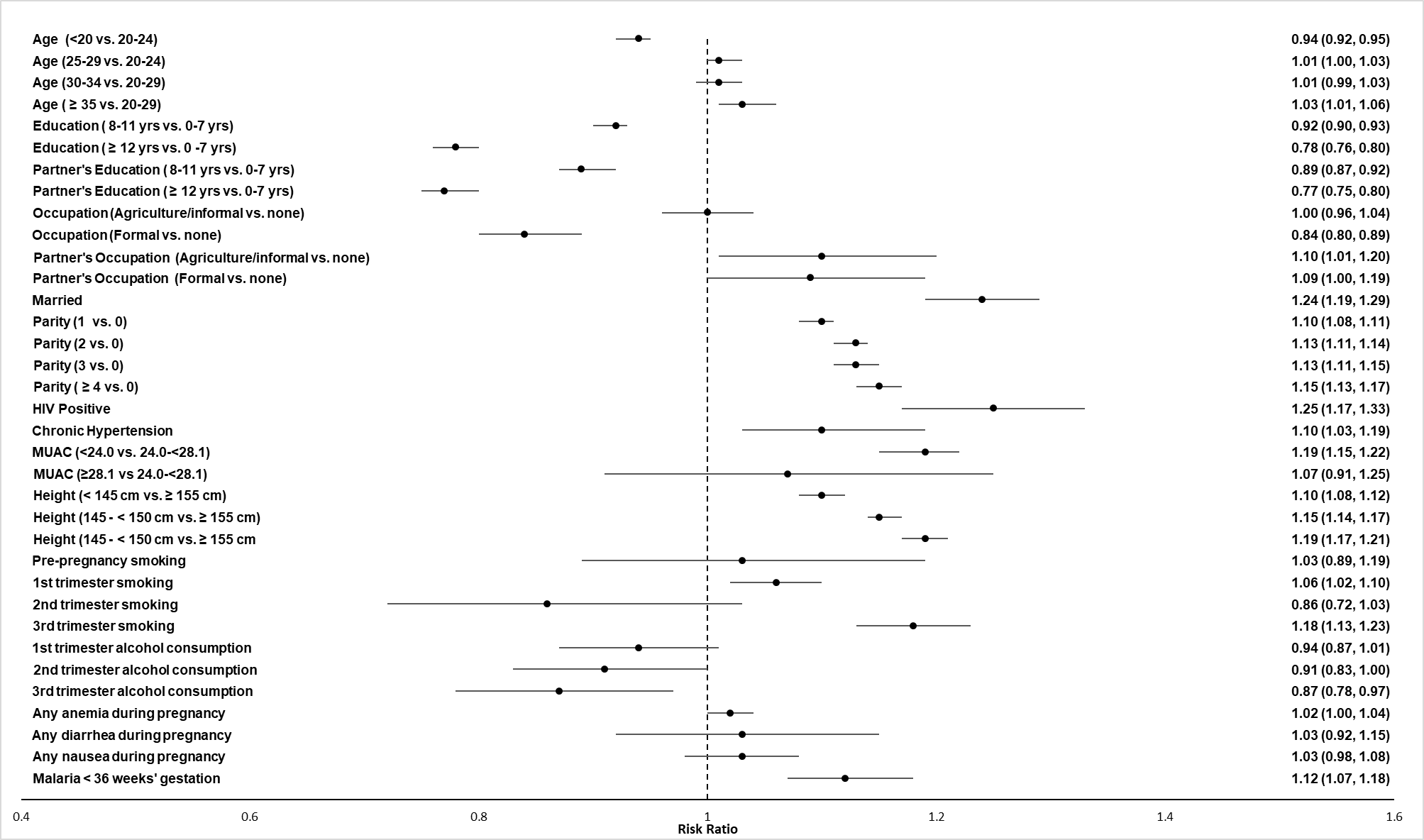


GWG=Gestational weight gain, BMI=body mass index, MUAC=mid-upper arm circumference, HIV=Human immunodeficiency virus, cm=centimeter

**Figure E2 in S1 Appendix.** Adjusted risk ratios and 95% confidence intervals for the associations between demongraphic, anthropometric, and clinical risk factors and inadequate GWG (1-stage model) among participants with underweight (n=19,735). Circles represent risk ratios and bars represent 95% confidence intervals.


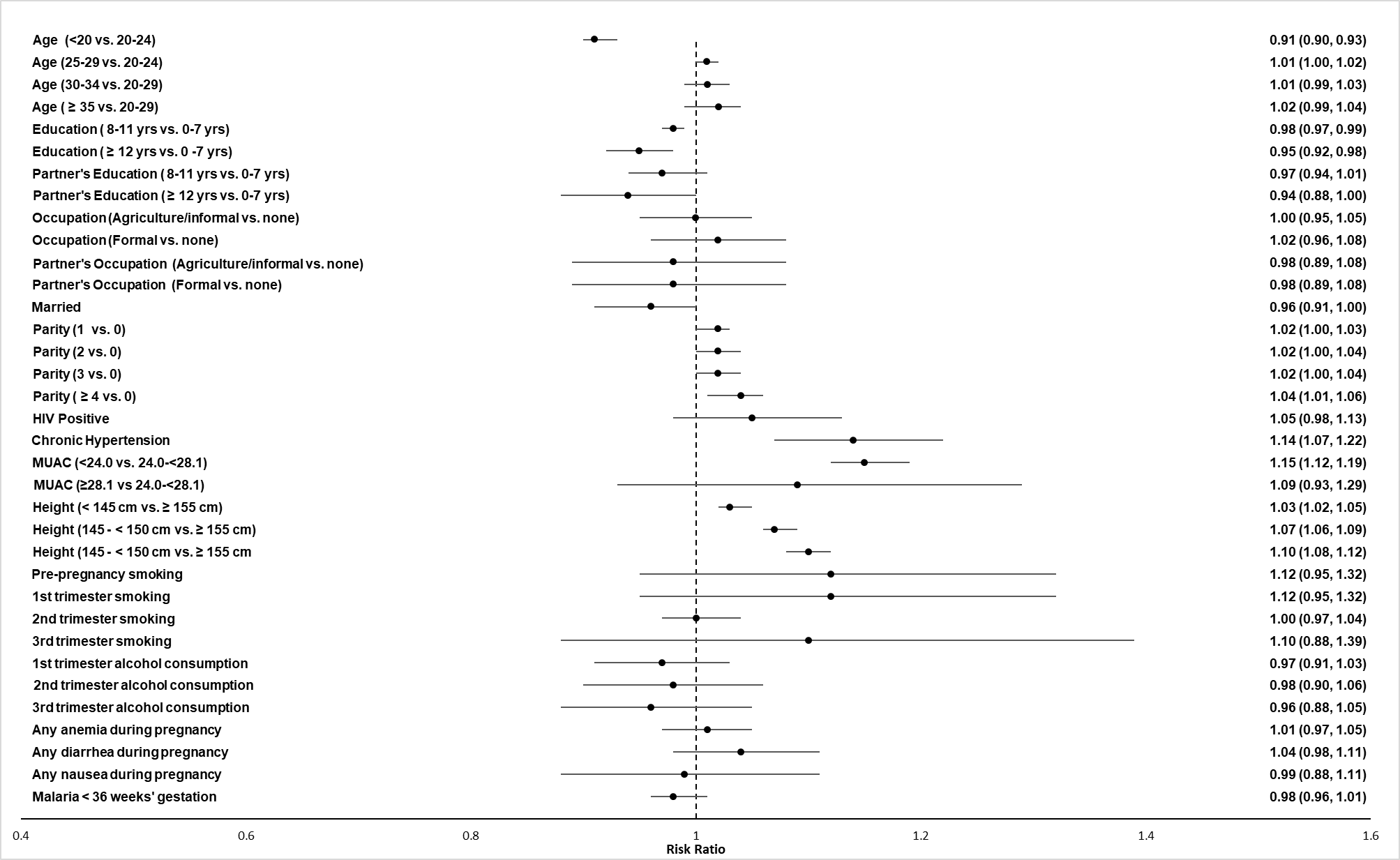


GWG=Gestational weight gain, BMI=body mass index, MUAC=mid-upper arm circumference, HIV=Human immunodeficiency virus, cm=centimeter

**Figure F1 in S1 Appendix.** Unadjusted risk ratios and 95% confidence intervals for the associations between demongraphic, anthropometric, and clinical risk factors and excessive GWG (1-stage model) among participants with underweight (n=19,735). Circles represent risk ratios and bars represent 95% confidence intervals.


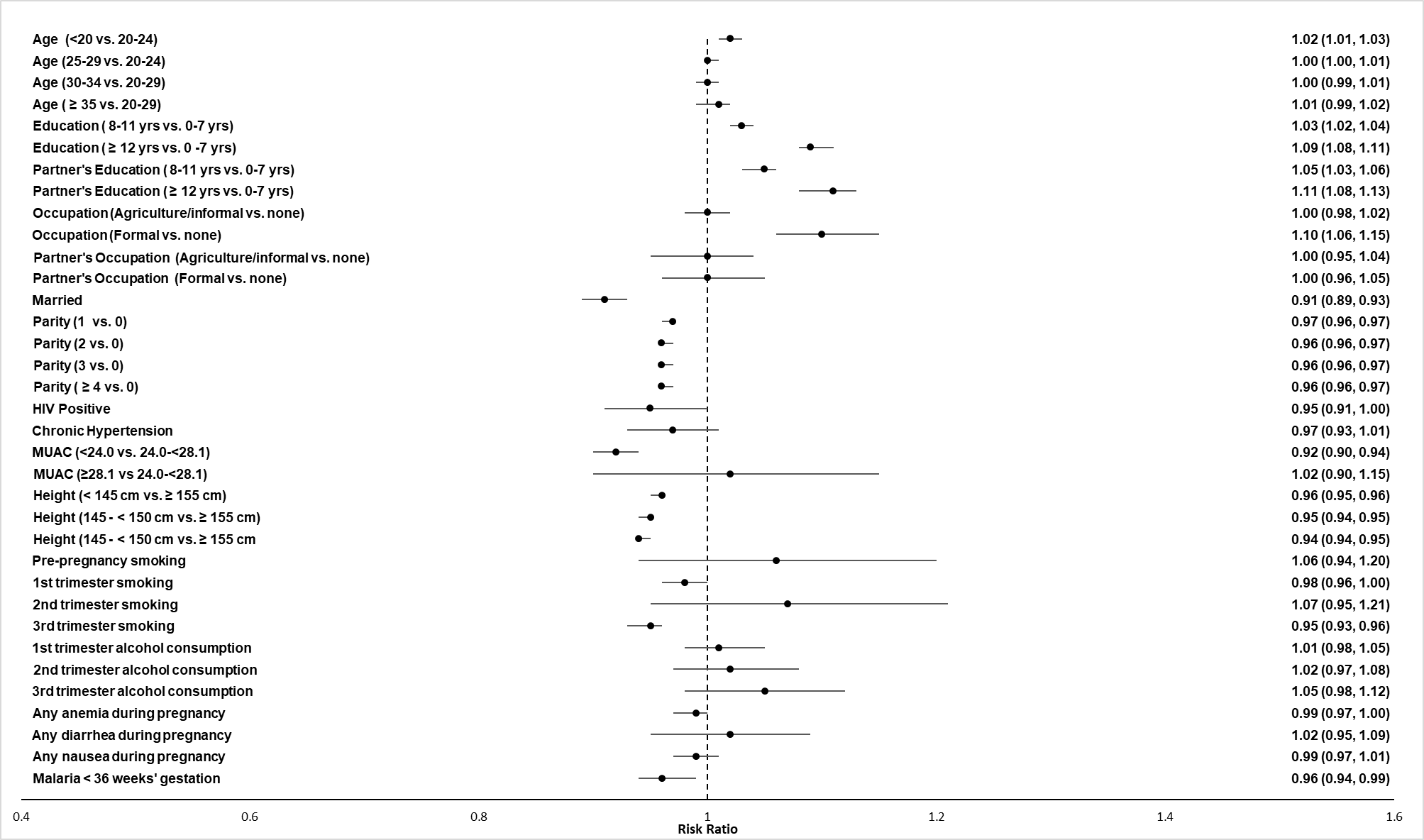


GWG=Gestational weight gain, BMI=body mass index, MUAC=mid-upper arm circumference, HIV=Human immunodeficiency virus, cm=centimeter

**Figure F2 in S1 Appendix.** Adjusted risk ratios and 95% confidence intervals for the associations between demongraphic, anthropometric, and clinical risk factors and excessive GWG (1-stage model) among participants with underweight (n=19,735). Circles represent risk ratios and bars represent 95% confidence intervals.


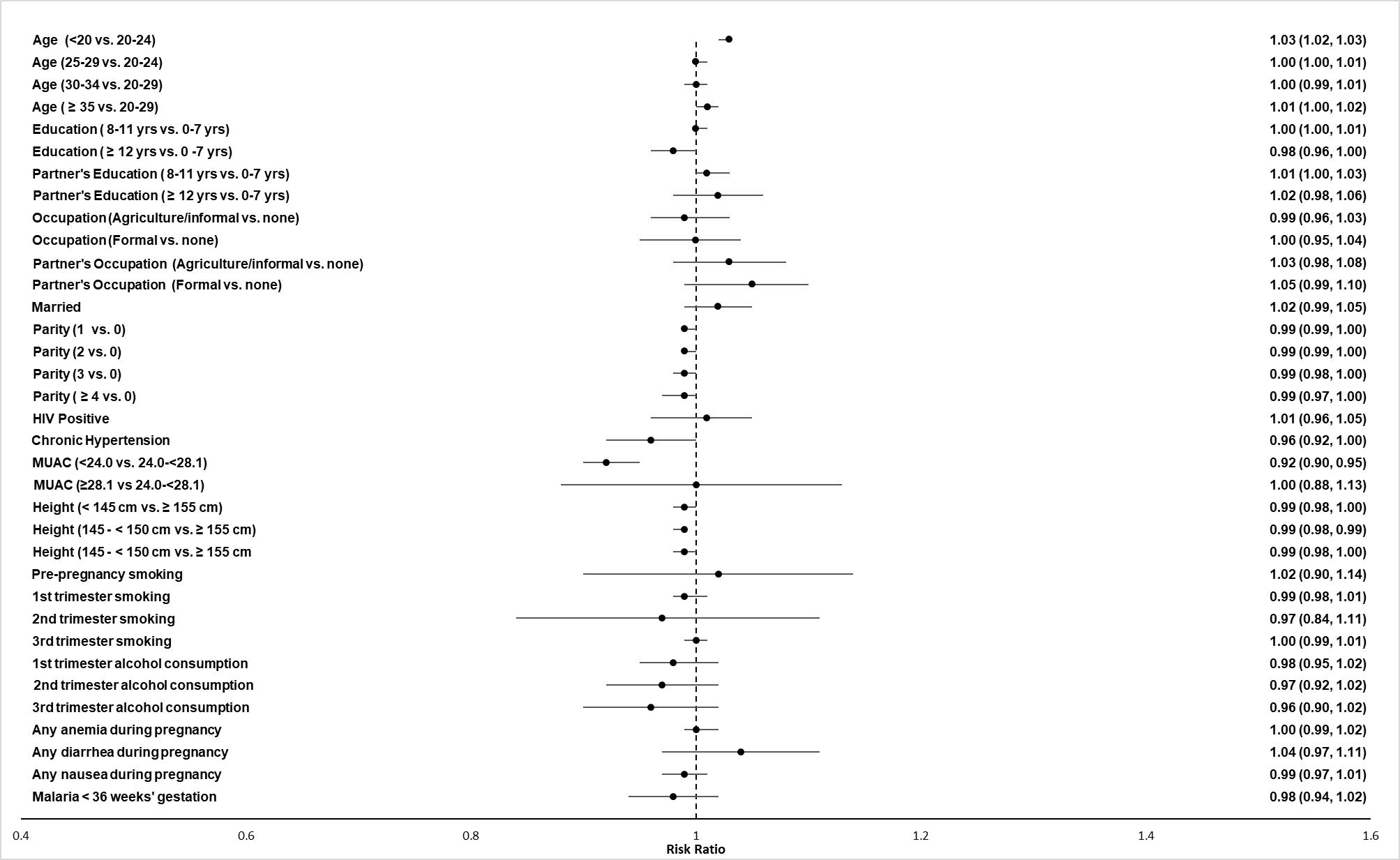


GWG=Gestational weight gain, BMI=body mass index, MUAC=mid-upper arm circumference, HIV=Human immunodeficiency virus, cm=centimeter

**Figure G1 in S1 Appendix.** Unadjusted risk ratios and 95% confidence intervals for the associations between demongraphic, anthropometric, and clinical risk factors and severely inadequate GWG (1-stage model) among participants with normal weight (n=51,047). Circles represent risk ratios and bars represent 95% confidence intervals.


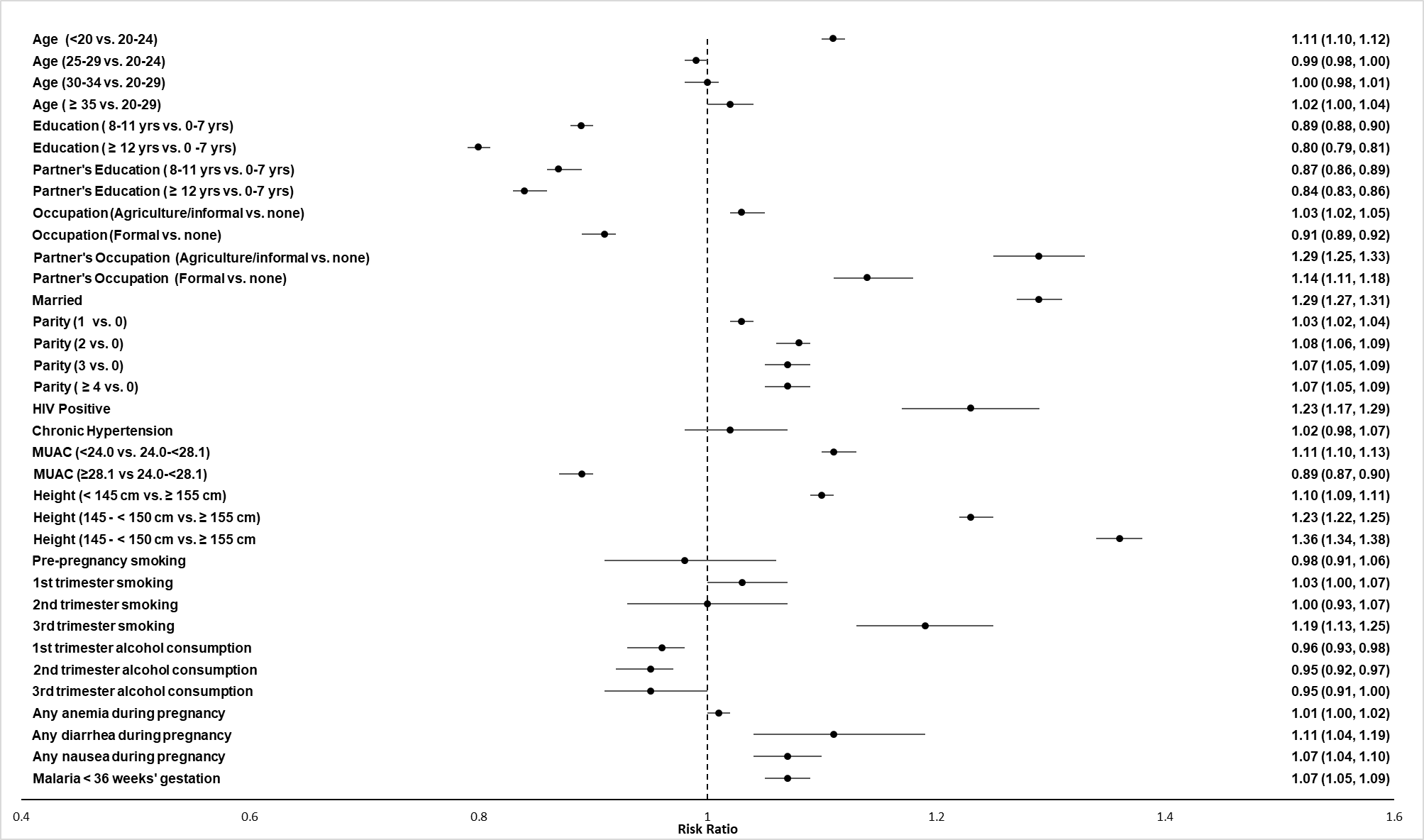


GWG=Gestational weight gain, BMI=body mass index, MUAC=mid-upper arm circumference, HIV=Human immunodeficiency virus, cm=centimeter

**Figure G2 in S1 Appendix.** Adjusted risk ratios and 95% confidence intervals for the associations between demongraphic, anthropometric, and clinical risk factors and severely inadequate GWG (1-stage model) among participants with normal weight (n=51,047). Circles represent risk ratios and bars represent 95% confidence intervals.


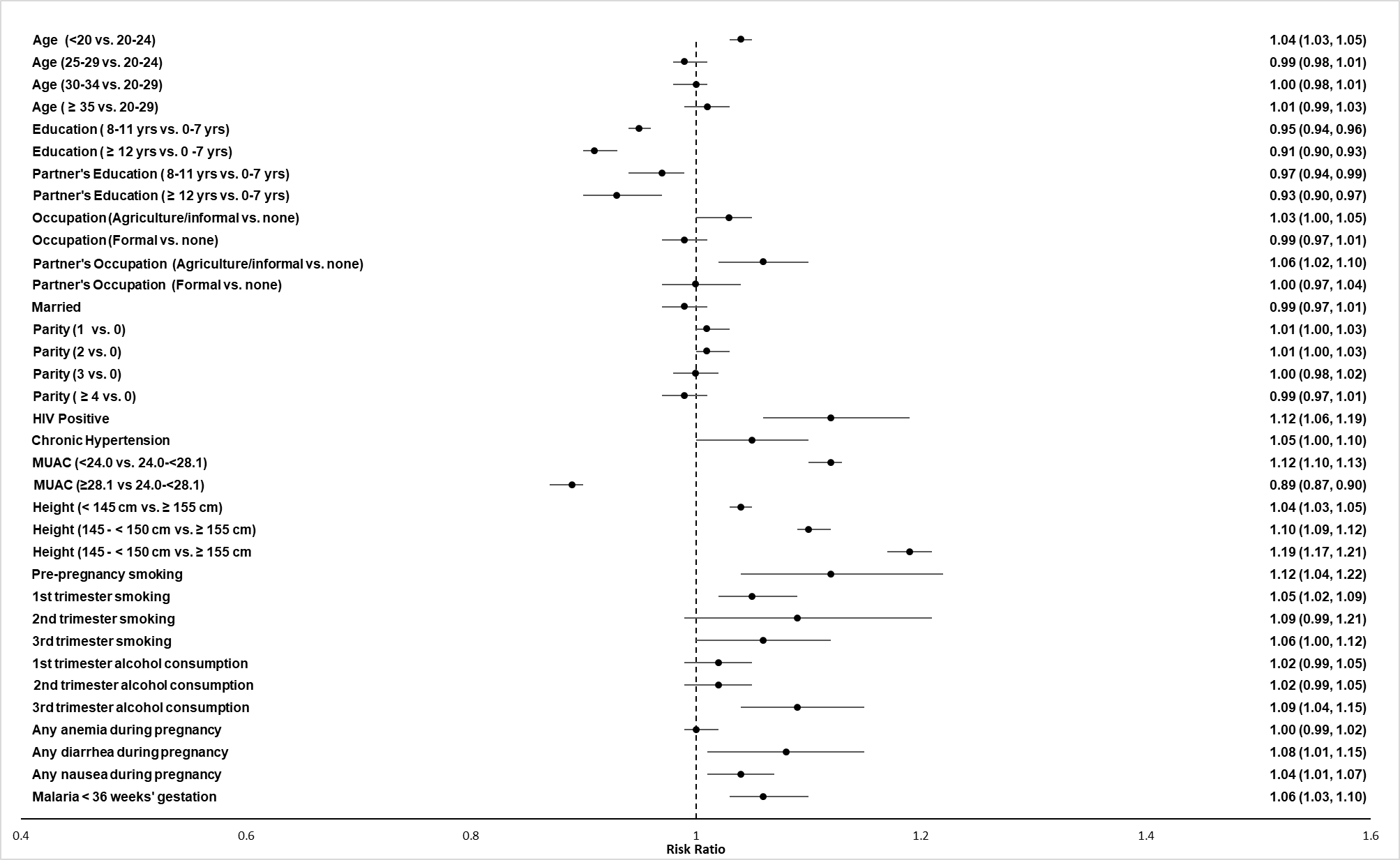


GWG=Gestational weight gain, BMI=body mass index, MUAC=mid-upper arm circumference, HIV=Human immunodeficiency virus, cm=centimeter

**Figure H1 in S1 Appendix.** Unadjusted risk ratios and 95% confidence intervals for the associations between demongraphic, anthropometric, and clinical risk factors and inadequate GWG (1-stage model) among participants with normal weight (n=51,047). Circles represent risk ratios and bars represent 95% confidence intervals.


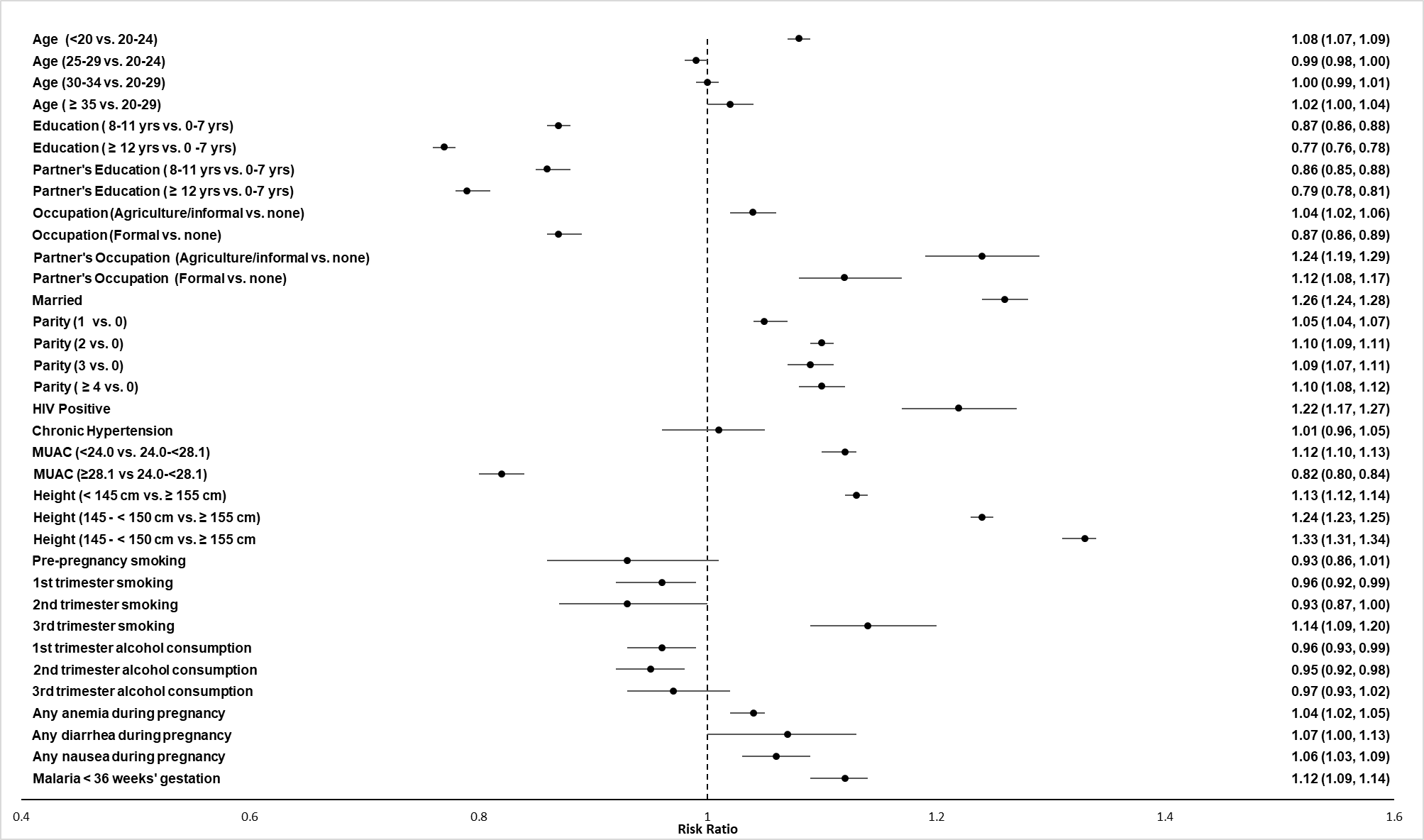


GWG=Gestational weight gain, BMI=body mass index, MUAC=mid-upper arm circumference, HIV=Human immunodeficiency virus, cm=centimeter

**Figure H2 in S1 Appendix.** Adjusted risk ratios and 95% confidence intervals for the associations between demongraphic, anthropometric, and clinical risk factors and inadequate GWG (1-stage model) among participants with normal weight (n=51,047). Circles represent risk ratios and bars represent 95% confidence intervals.


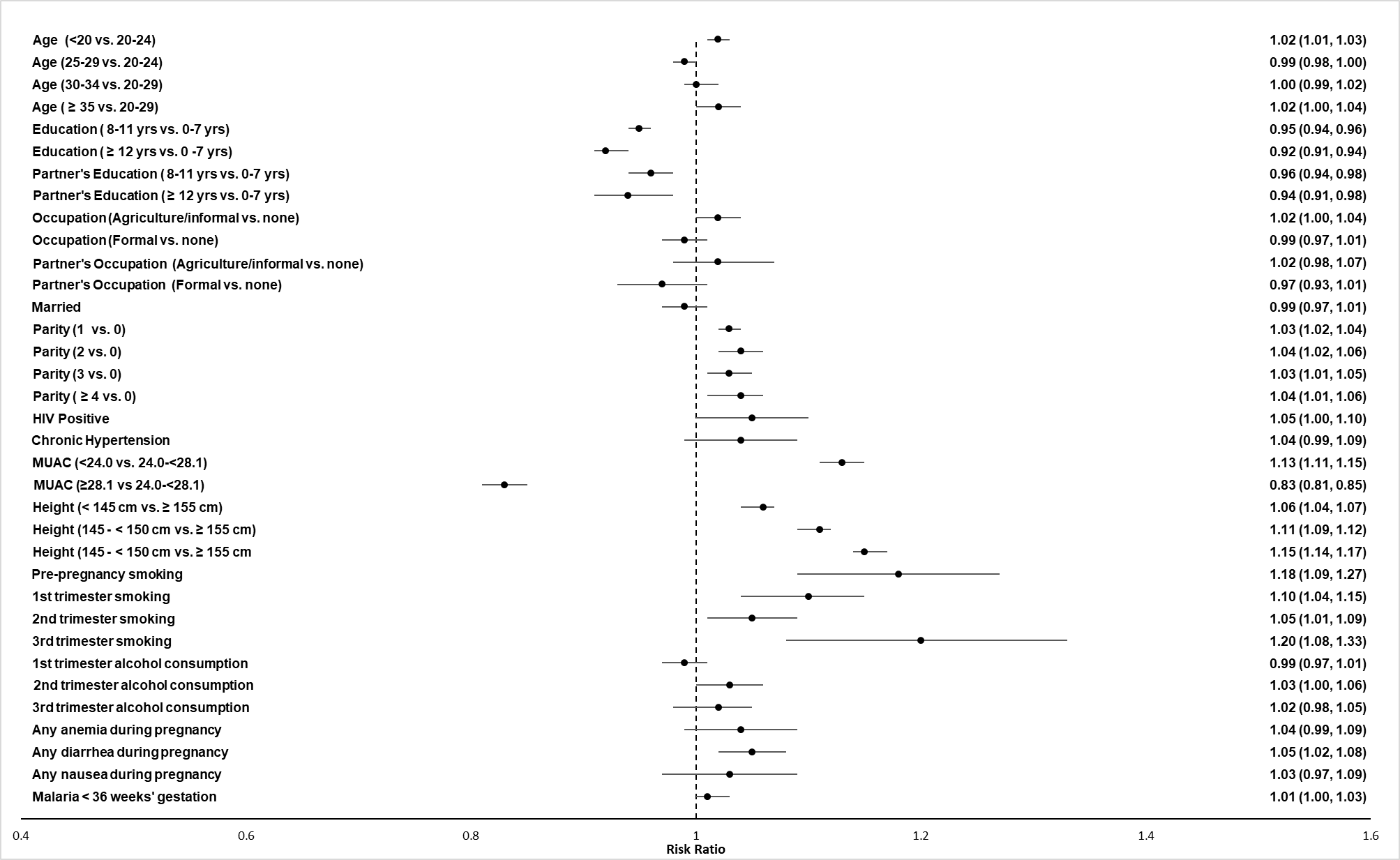


GWG=Gestational weight gain, BMI=body mass index, MUAC=mid-upper arm circumference, HIV=Human immunodeficiency virus, cm=centimeter

**Figure I1 in S1 Appendix.** Unadjusted risk ratios and 95% confidence intervals for the associations between demongraphic, anthropometric, and clinical risk factors and excessive GWG (1-stage model) among participants with normal weight (n=51,047). Circles represent risk ratios and bars represent 95% confidence intervals.


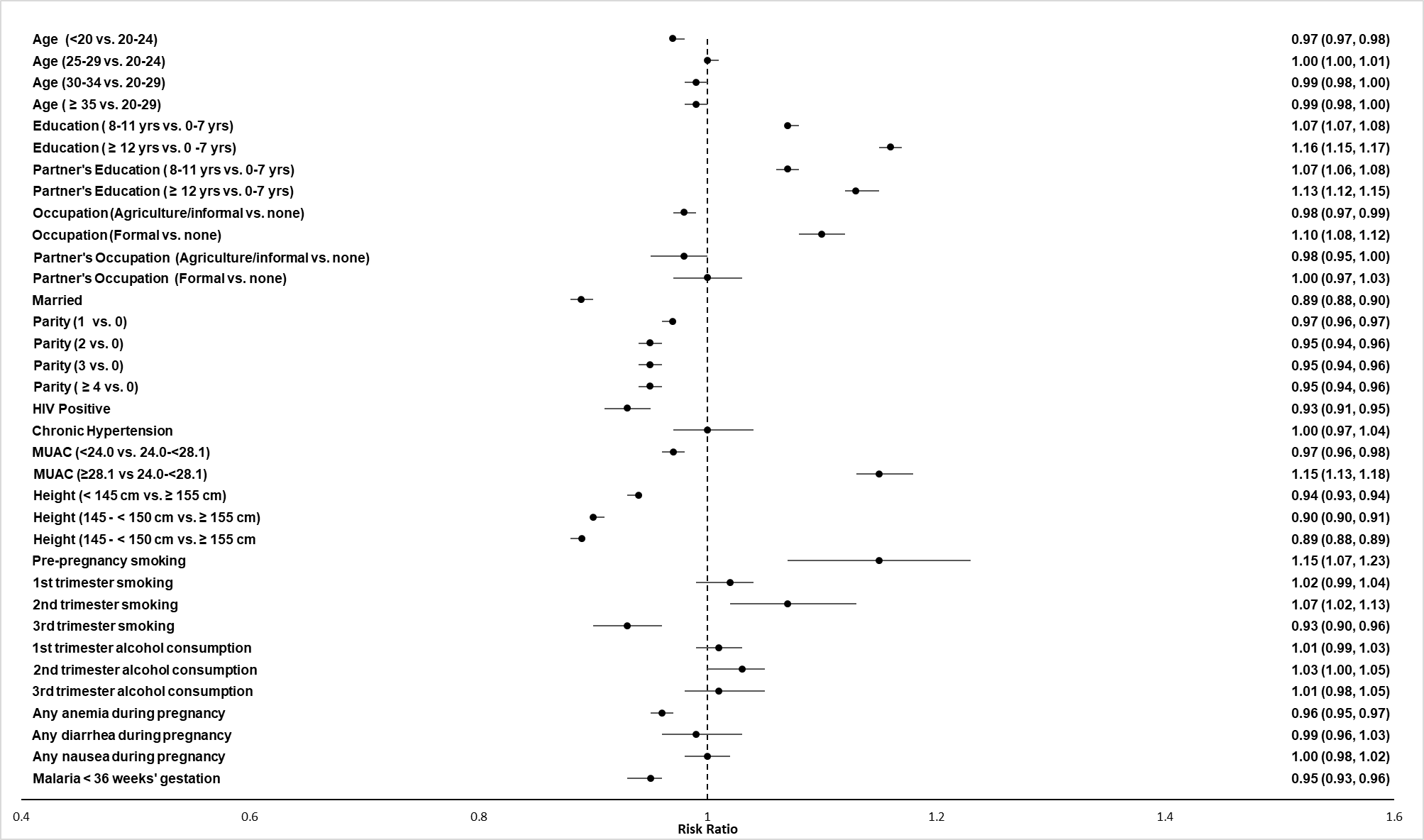


GWG=Gestational weight gain, BMI=body mass index, MUAC=mid-upper arm circumference, HIV=Human immunodeficiency virus, cm=centimeter

**Figure I2 in S1 Appendix.** Adjusted risk ratios and 95% confidence intervals for the associations between demongraphic, anthropometric, and clinical risk factors and excessive GWG (1-stage model) among participants with normal weight (n=51,047). Circles represent risk ratios and bars represent 95% confidence intervals.


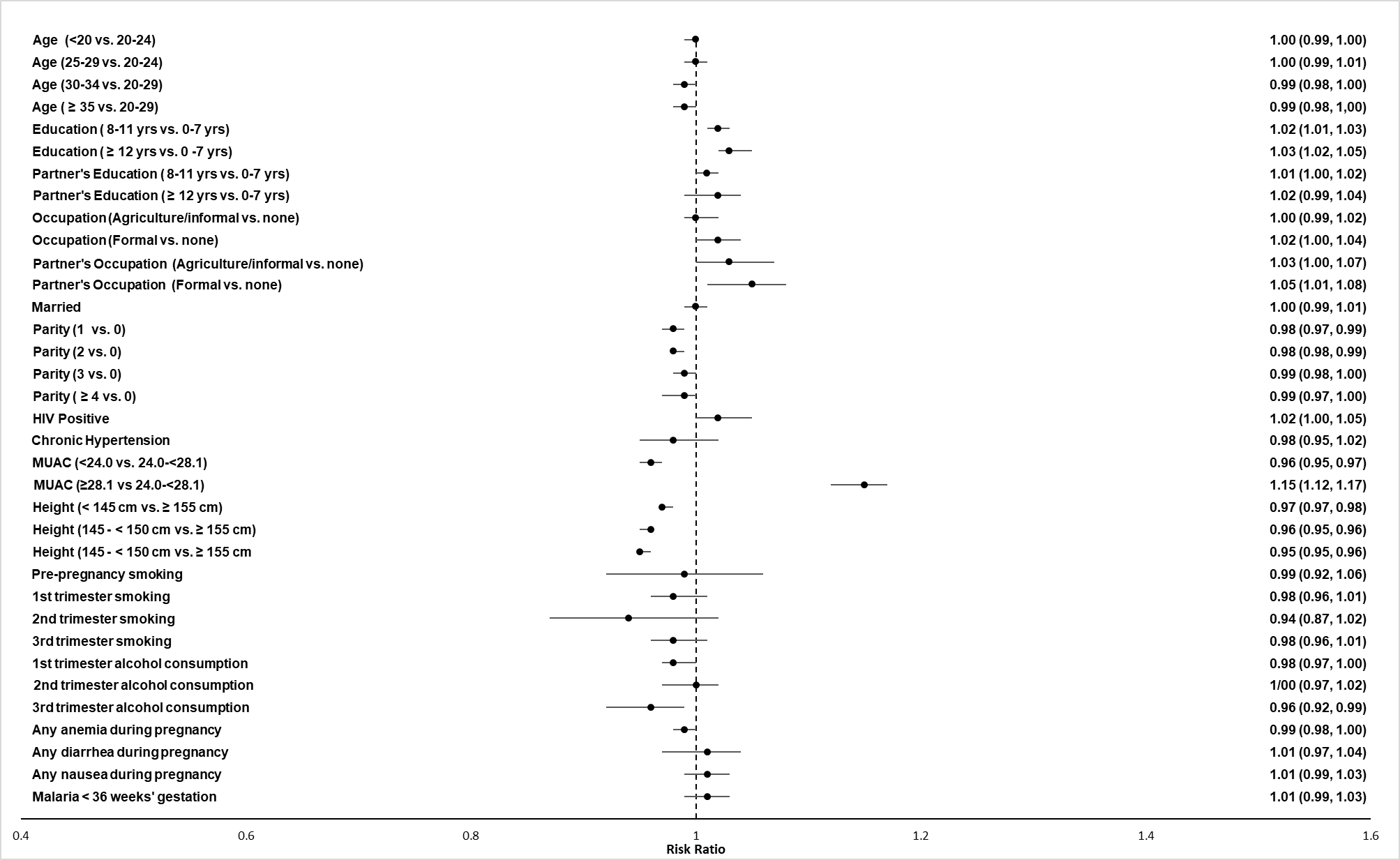


GWG=Gestational weight gain, BMI=body mass index, MUAC=mid-upper arm circumference, HIV=Human immunodeficiency virus, cm=centimeter

**Figure J1 in S1 Appendix.** Unadjusted risk ratios and 95% confidence intervals for the associations between demongraphic, anthropometric, and clinical risk factors and severely inadequate GWG (1-stage model) among participants with overweight and obesity (n=9,166). Circles represent risk ratios and bars represent 95% confidence intervals.


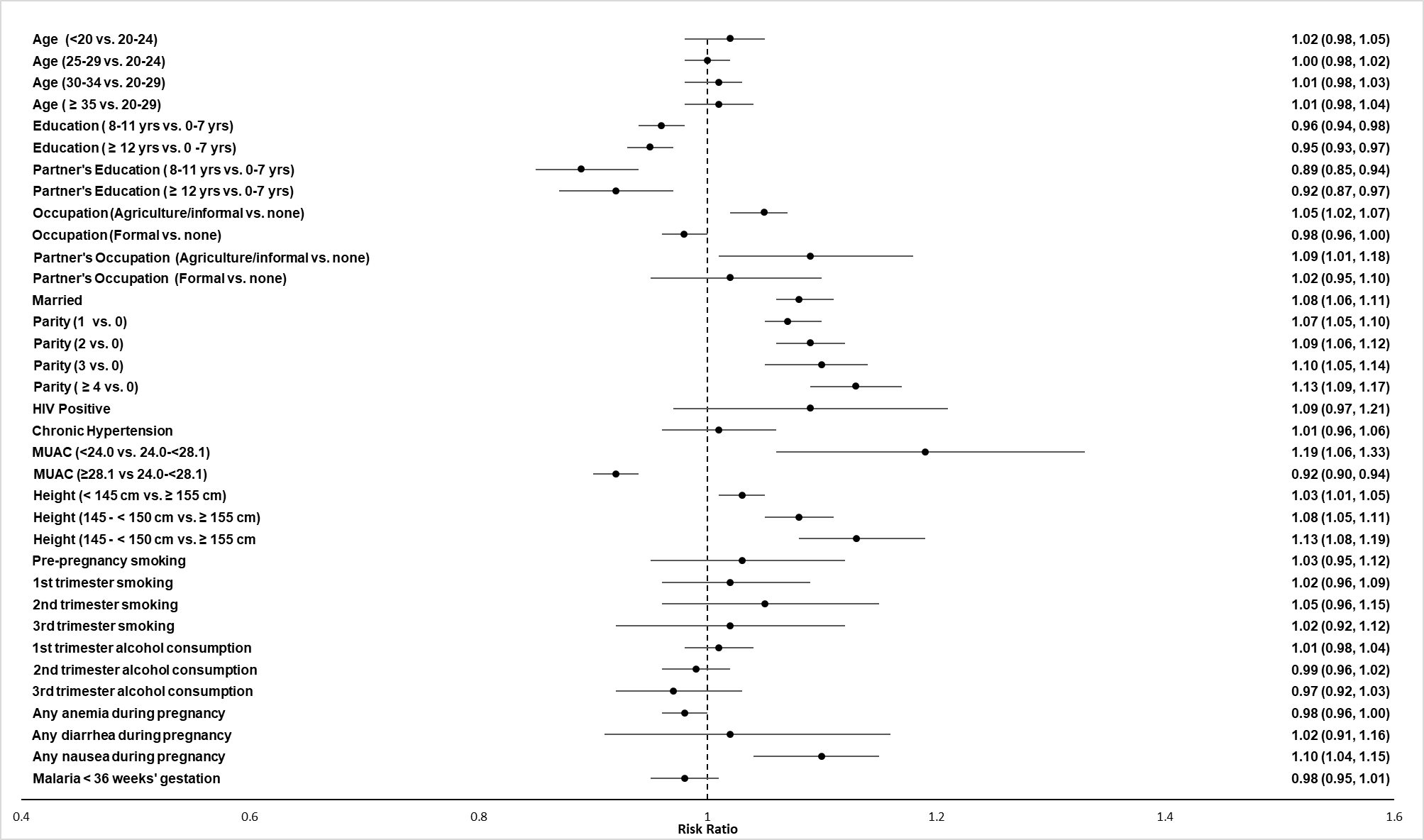


GWG=Gestational weight gain, BMI=body mass index, MUAC=mid-upper arm circumference, HIV=Human immunodeficiency virus, cm=centimeter

**Figure J2 in S1 Appendix.** Adjusted risk ratios and 95% confidence intervals for the associations between demongraphic, anthropometric, and clinical risk factors and severely inadequate GWG (1-stage model) among participants with overweight and obesity (n=9,166). Circles represent risk ratios and bars represent 95% confidence intervals.


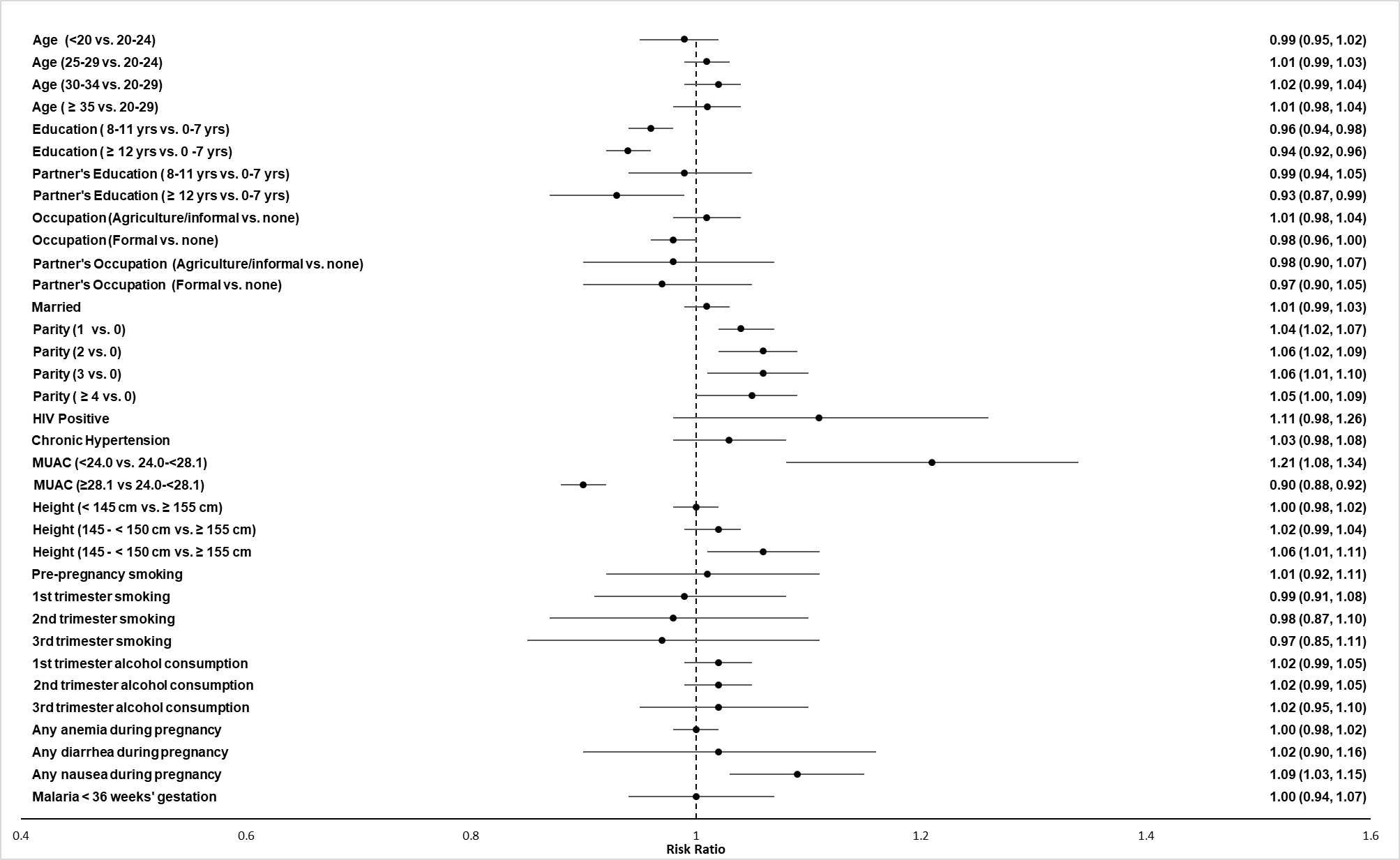


GWG=Gestational weight gain, BMI=body mass index, MUAC=mid-upper arm circumference, HIV=Human immunodeficiency virus, cm=centimeter

**Figure K1 in S1 Appendix.** Unadjusted risk ratios and 95% confidence intervals for the associations between demongraphic, anthropometric, and clinical risk factors and inadequate GWG (1-stage model) among participants with overweight and obesity (n=9,166). Circles represent risk ratios and bars represent 95% confidence intervals.


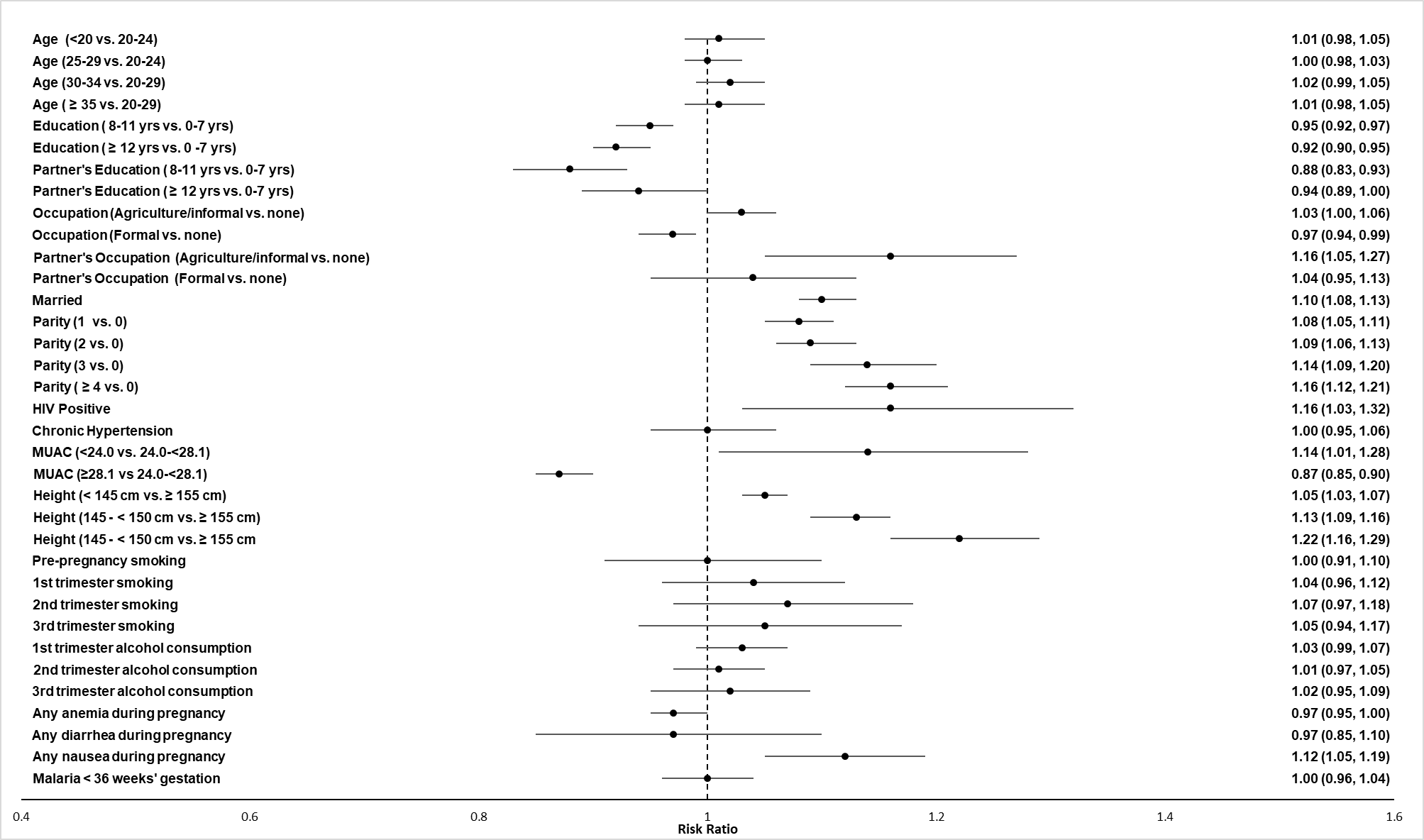


GWG=Gestational weight gain, BMI=body mass index, MUAC=mid-upper arm circumference, HIV=Human immunodeficiency virus, cm=centimeter

**Figure K2 in S1 Appendix.** Adjusted risk ratios and 95% confidence intervals for the associations between demongraphic, anthropometric, and clinical risk factors and inadequate GWG (1-stage model) among participants with overweight and obesity (n=9,166). Circles represent risk ratios and bars represent 95% confidence intervals.


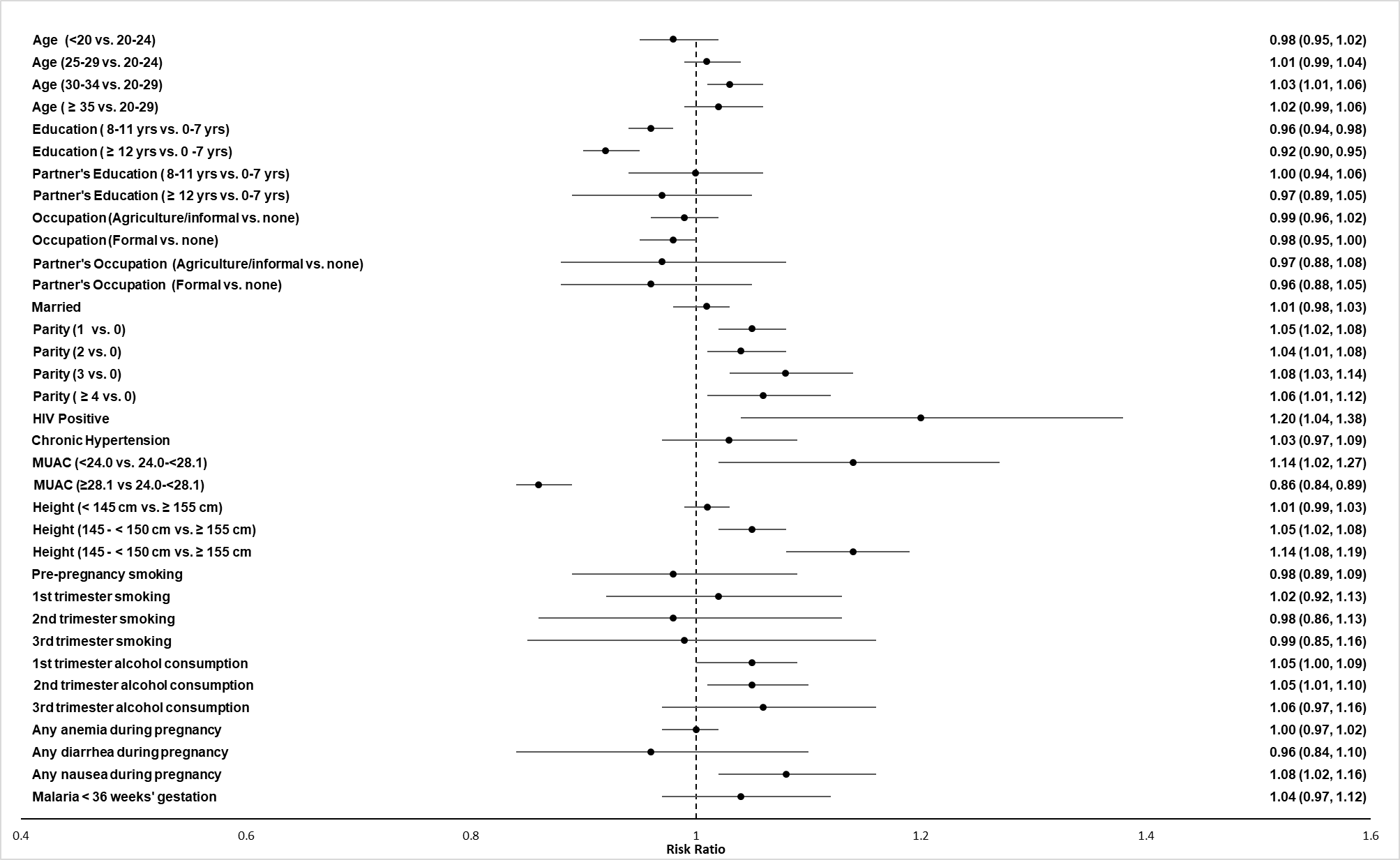


GWG=Gestational weight gain, BMI=body mass index, MUAC=mid-upper arm circumference, HIV=Human immunodeficiency virus, cm=centimeter

**Figure L1 in S1 Appendix.** Unadjusted risk ratios and 95% confidence intervals for the associations between demongraphic, anthropometric, and clinical risk factors and excessive GWG (1-stage model) among participants with overweight and obesity (n=9,166). Circles represent risk ratios and bars represent 95% confidence intervals.


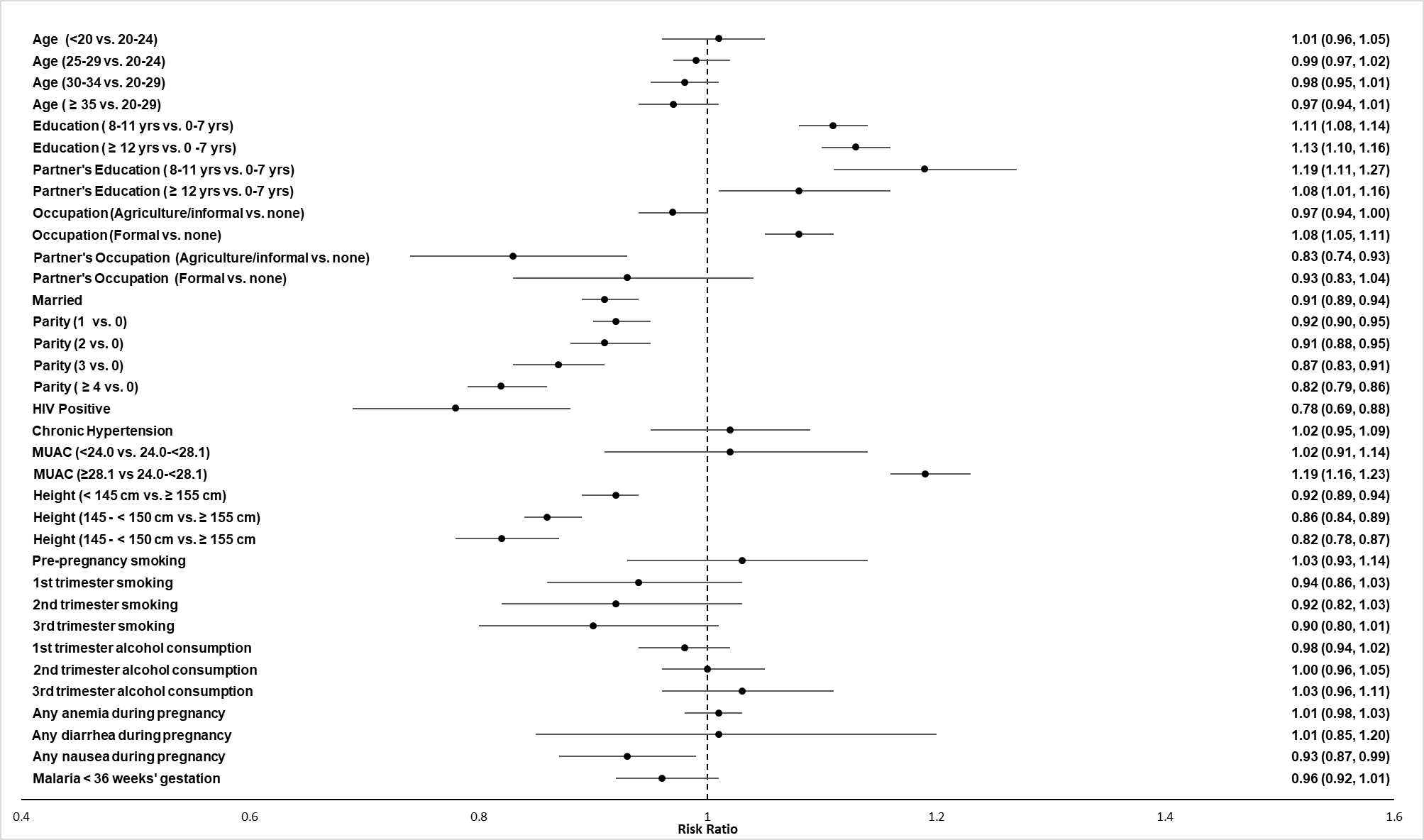


GWG=Gestational weight gain, BMI=body mass index, MUAC=mid-upper arm circumference, HIV=Human immunodeficiency virus, cm=centimeter

**Figure L2 in S1 Appendix.** Adjusted risk ratios and 95% confidence intervals for the associations between demongraphic, anthropometric, and clinical risk factors and excessive GWG (1-stage model) among participants with overweight and obesity (n=9,166). Circles represent risk ratios and bars represent 95% confidence intervals.


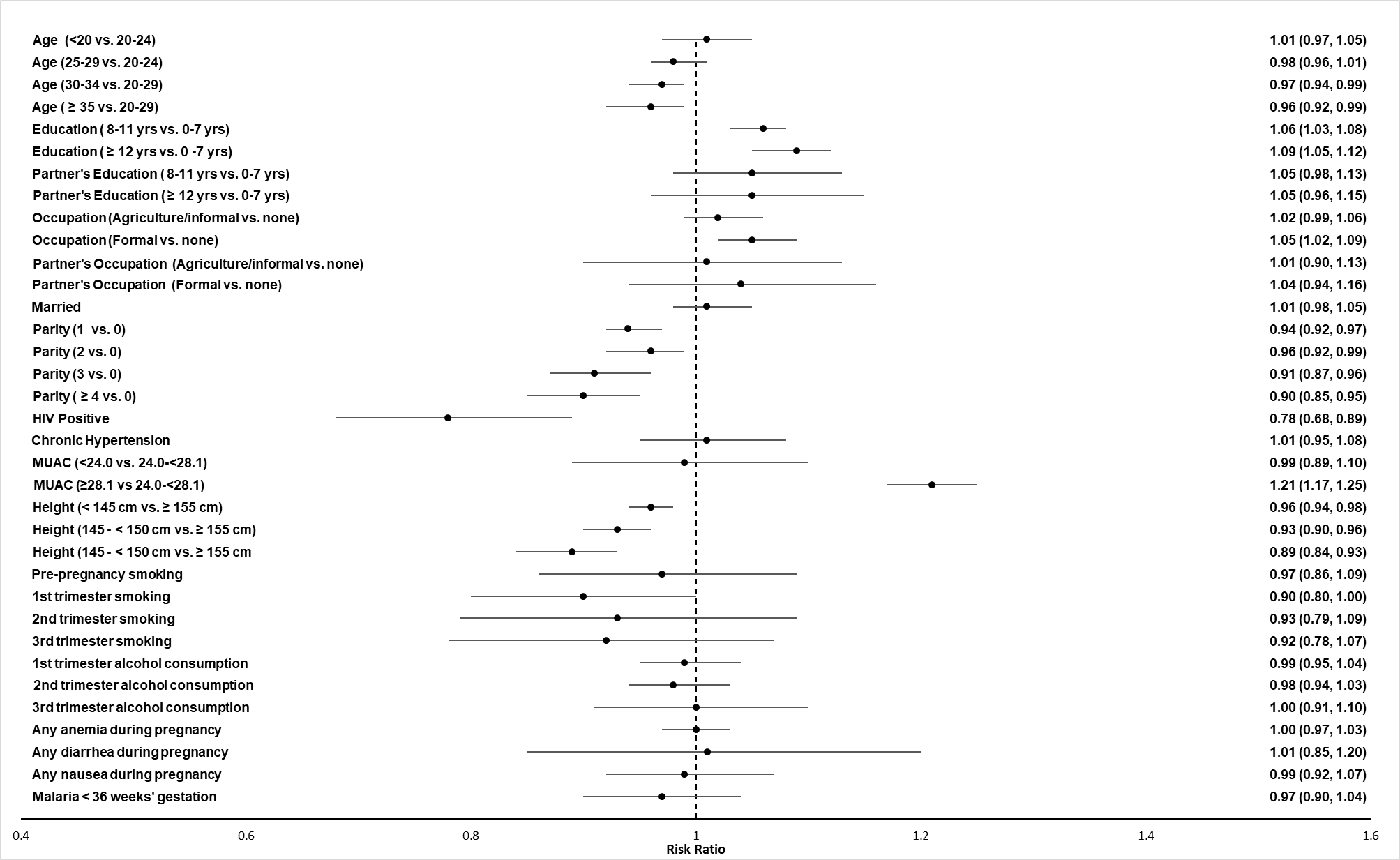


GWG=Gestational weight gain, BMI=body mass index, MUAC=mid-upper arm circumference, HIV=Human immunodeficiency virus, cm=centimeter

**Figure M1 in S1 Appendix.** Unadjusted risk ratios and 95% confidence intervals for the associations between demongraphic, anthropometric, and clinical risk factors and weight gain z-score < -2 based on the INTERGROWTH-21^st^ standard (1-stage model) among women with normal weight (n=51,047). Circles represent risk ratios and bars represent 95% confidence intervals.


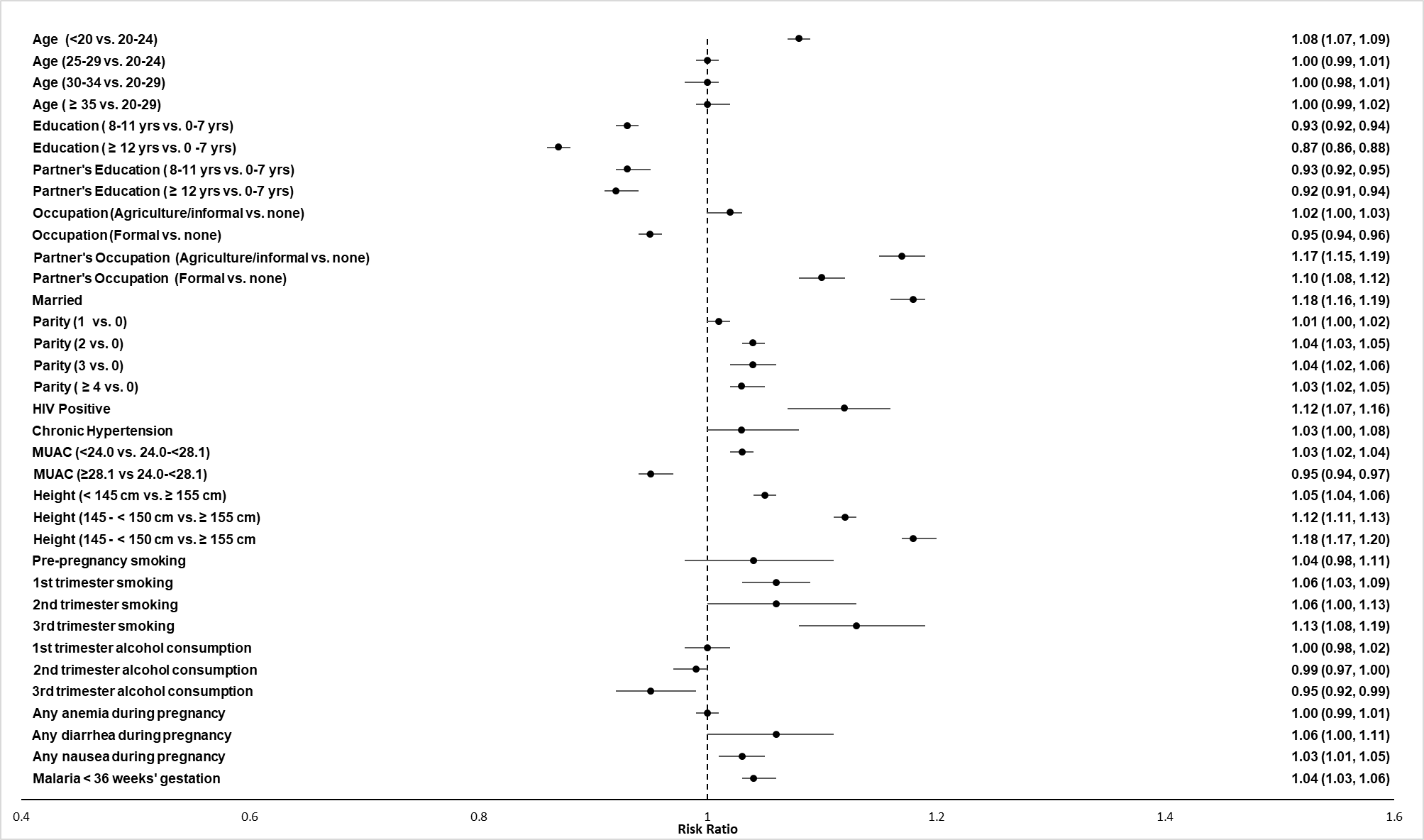


GWG=Gestational weight gain, BMI=body mass index, MUAC=mid-upper arm circumference, HIV=Human immunodeficiency virus, cm=centimeter

**Figure M2 in S1 Appendix.** Adjusted risk ratios and 95% confidence intervals for the associations between demongraphic, anthropometric, and clinical risk factors and weight gain z-score < -2 based on the INTERGROWTH-21^st^ standard (1-stage model) among women with normal weight (n=51,047). Circles represent risk ratios and bars represent 95% confidence intervals.


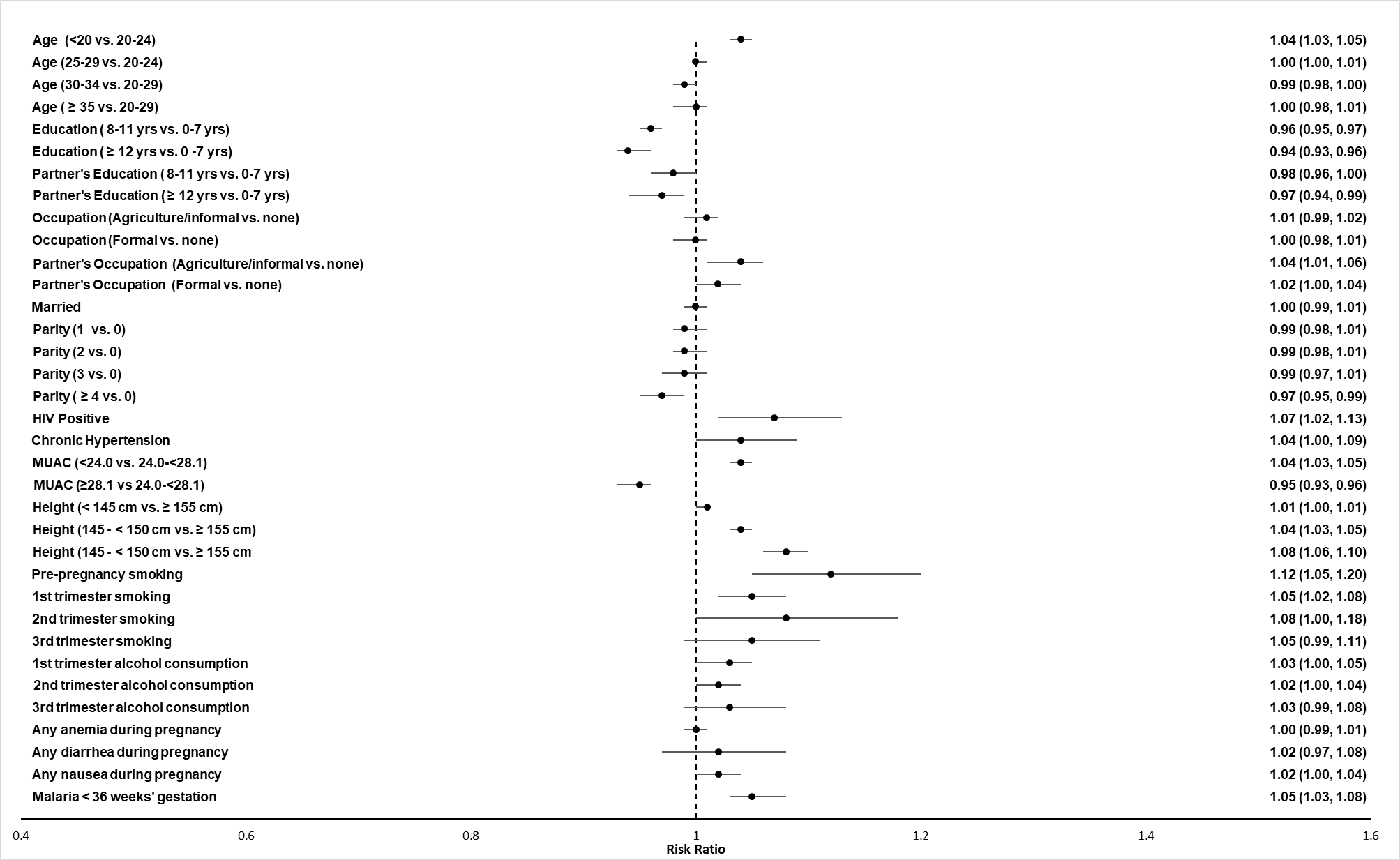


GWG=Gestational weight gain, BMI=body mass index, MUAC=mid-upper arm circumference, HIV=Human immunodeficiency virus, cm=centimeter

**Figure N1 in S1 Appendix.** Unadjusted risk ratios and 95% confidence intervals for the associations between demongraphic, anthropometric, and clinical risk factors and weight gain z-score < -1 based on the INTERGROWTH-21^st^ standard (1-stage model) among women with normal weight (n=51,047). Circles represent risk ratios and bars represent 95% confidence intervals.


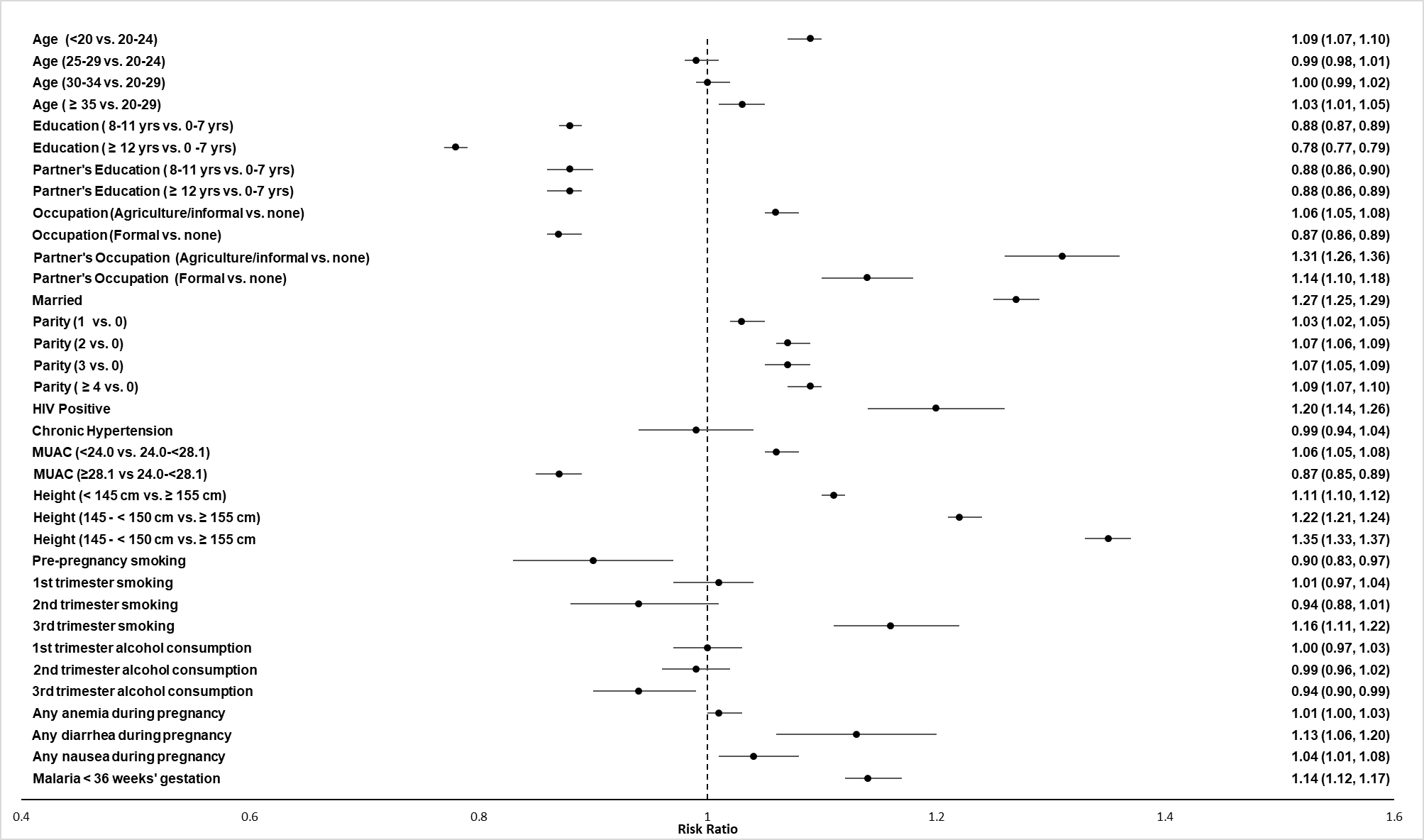


GWG=Gestational weight gain, BMI=body mass index, MUAC=mid-upper arm circumference, HIV=Human immunodeficiency virus, cm=centimeter

**Figure N2 in S1 Appendix.** Adjusted risk ratios and 95% confidence intervals for the associations between demongraphic, anthropometric, and clinical risk factors and weight gain z-score < -1 based on the INTERGROWTH-21^st^ standard (1-stage model) among women with normal weight (n=51,047). Circles represent risk ratios and bars represent 95% confidence intervals.


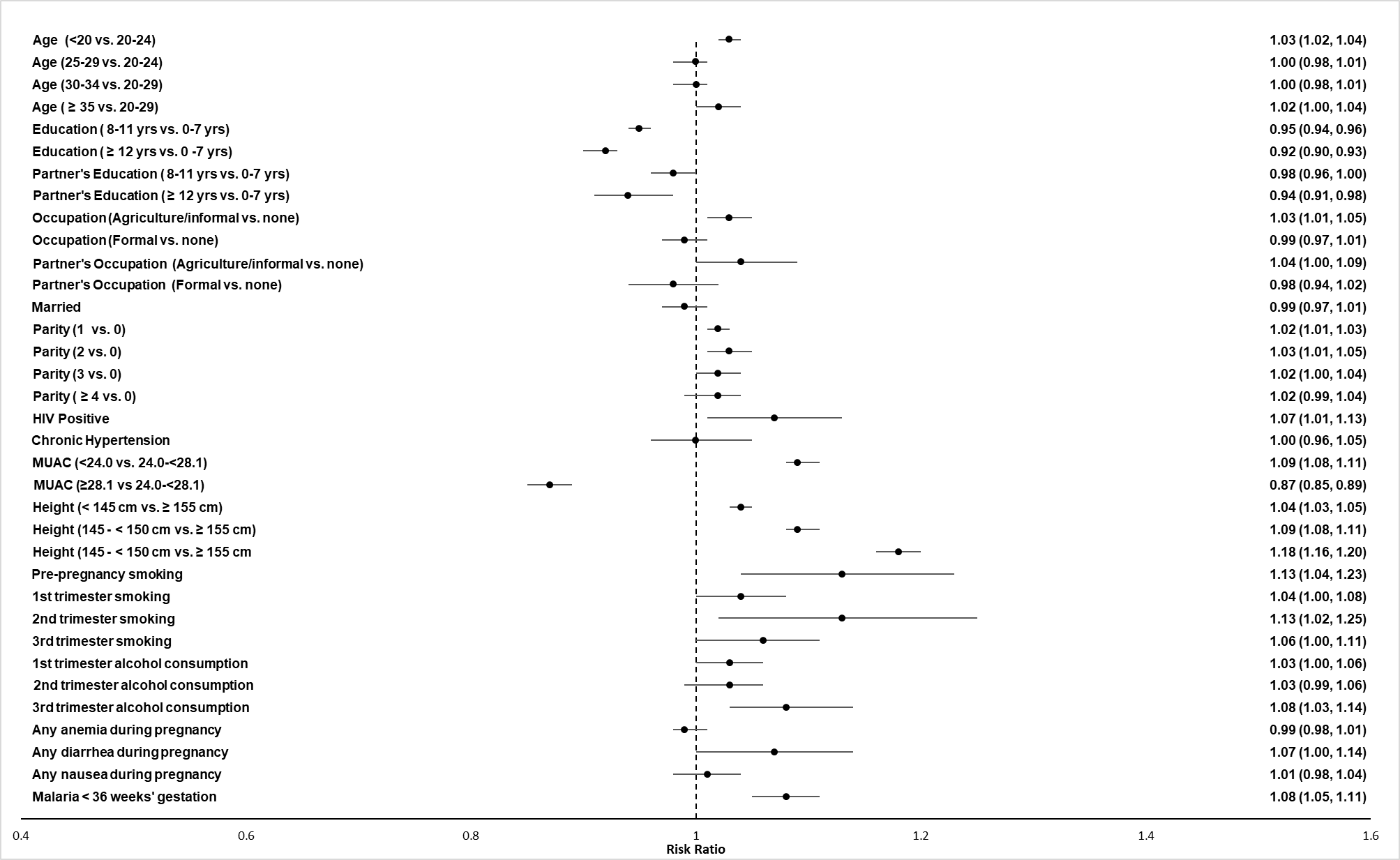


GWG=Gestational weight gain, BMI=body mass index, MUAC=mid-upper arm circumference, HIV=Human immunodeficiency virus, cm=centimeter

**Figure O1 in S1 Appendix.** Unadjusted risk ratios and 95% confidence intervals for the associations between demongraphic, anthropometric, and clinical risk factors and weight gain z-score > 1 based on the INTERGROWTH-21^st^ standard (1-stage model) among women with normal weight. Circles represent risk ratios and bars represent 95% confidence intervals.


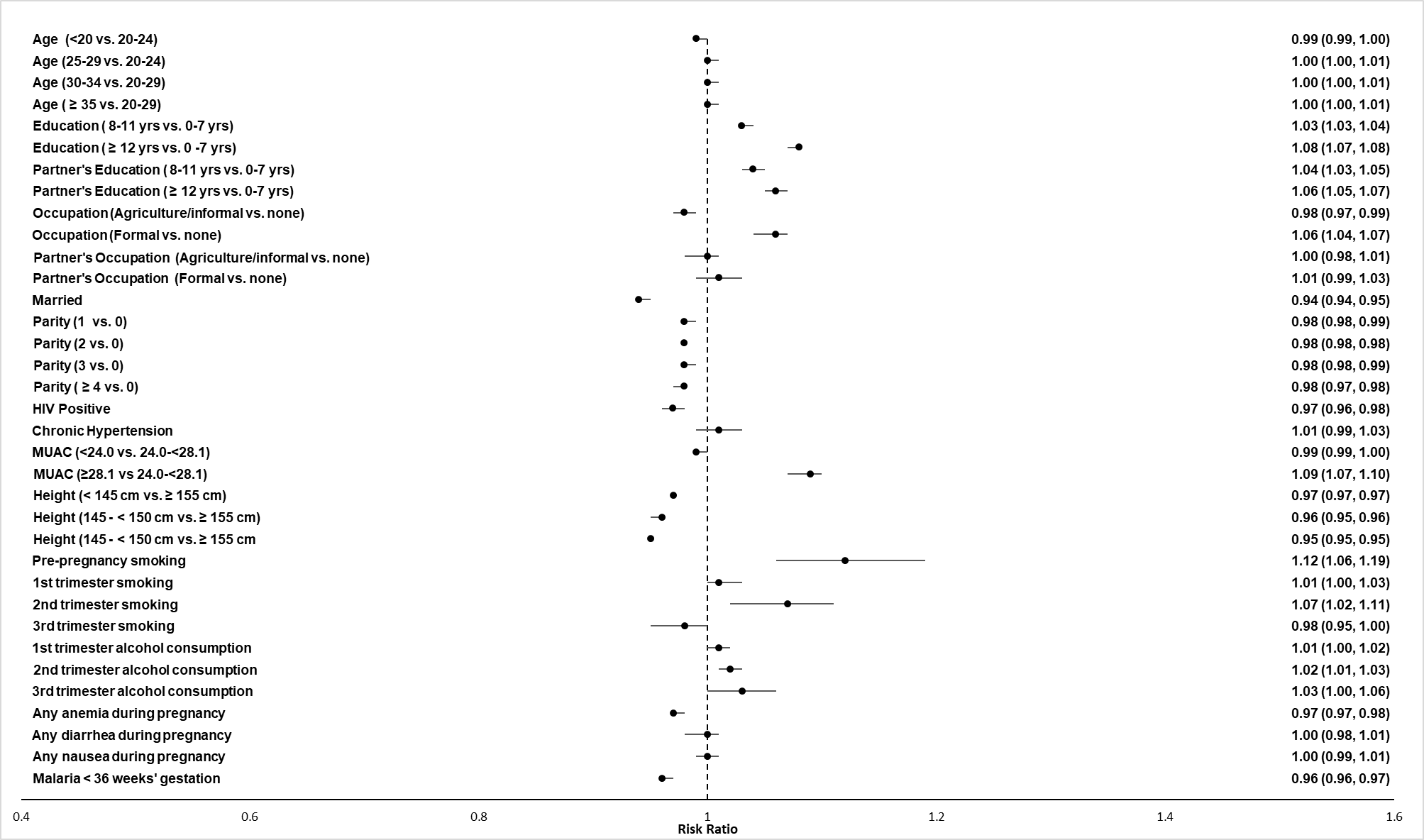


GWG=Gestational weight gain, BMI=body mass index, MUAC=mid-upper arm circumference, HIV=Human immunodeficiency virus, cm=centimeter

**Figure O2 in S1 Appendix.** Adjusted eisk ratios and 95% confidence intervals for the associations between demongraphic, anthropometric, and clinical risk factors and weight gain z-score > 1 based on the INTERGROWTH-21^st^ standard (1-stage model) among women with normal weight (n=51,047). Circles represent risk ratios and bars represent 95% confidence intervals.


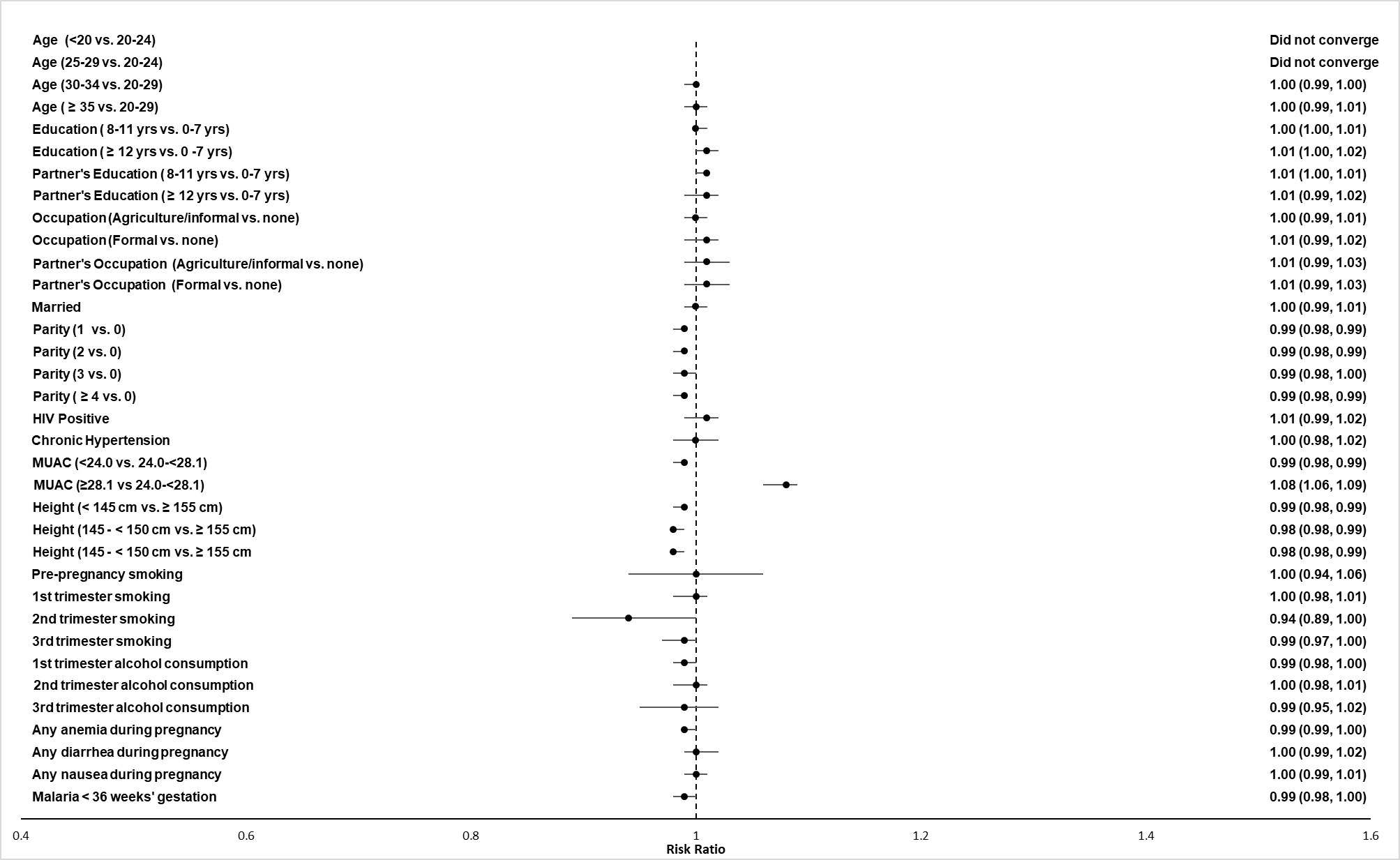


GWG=Gestational weight gain, BMI=body mass index, MUAC=mid-upper arm circumference, HIV=Human immunodeficiency virus, cm=centimeter

**Figure P1 in S1 Appendix.** Unadjusted risk ratios and 95% confidence intervals for the associations between demongraphic, anthropometric, substance use, and clinical risk factors and severely inadequate GWG (1-stage model) among women with a third trimester weight measurement (n=69,659). Circles represent risk ratios and bars represent 95% confidence intervals.


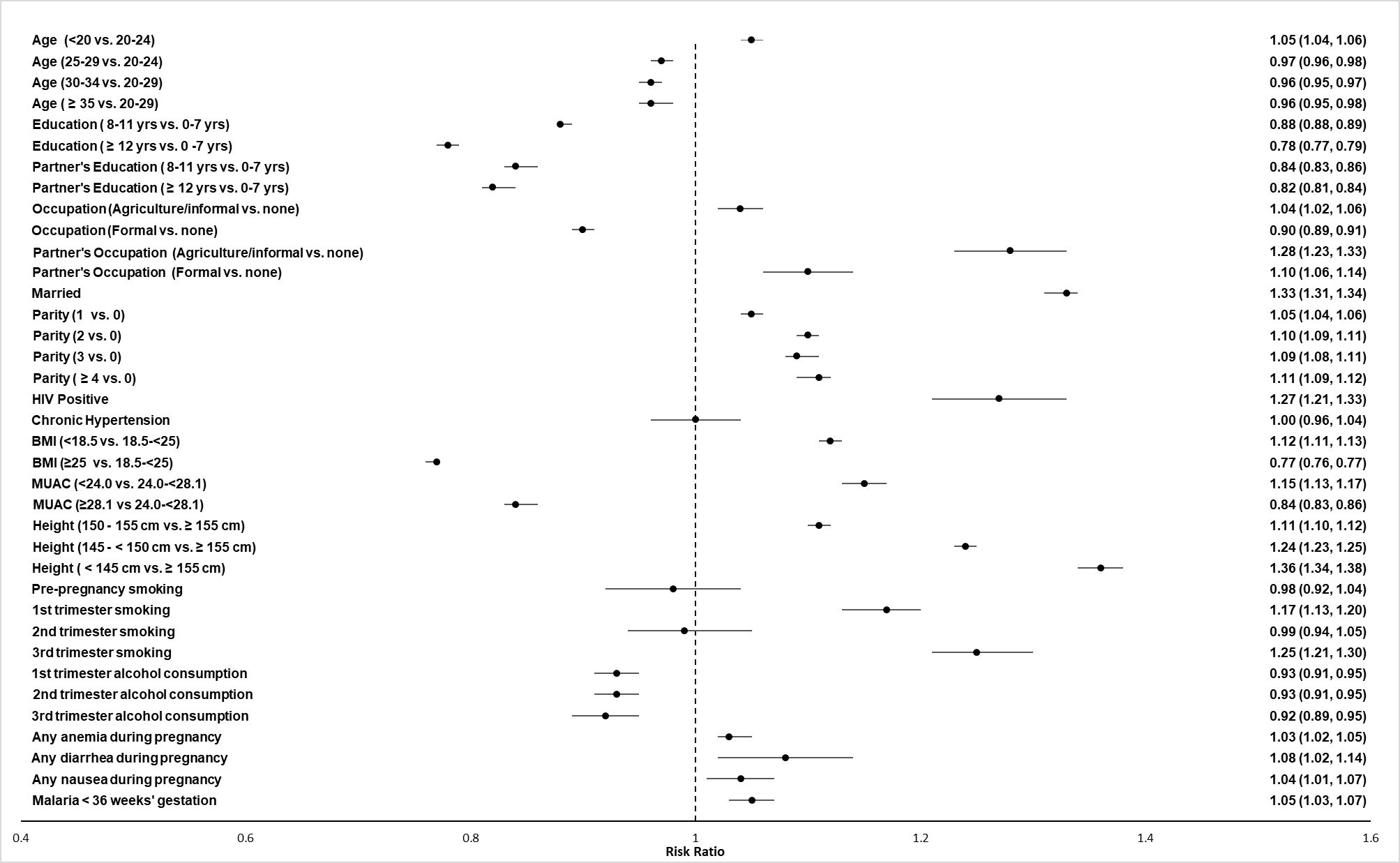


GWG=Gestational weight gain, BMI=body mass index, MUAC=mid-upper arm circumference, HIV=Human immunodeficiency virus, cm=centimeter

**Figure P2 in S1 Appendix.** Adjusted risk ratios and 95% confidence intervals for the associations between demongraphic, anthropometric, substance use, and clinical risk factors and severely inadequate GWG (1-stage model) among women with a third trimester weight measurement (n=69,659). Circles represent risk ratios and bars represent 95% confidence intervals.


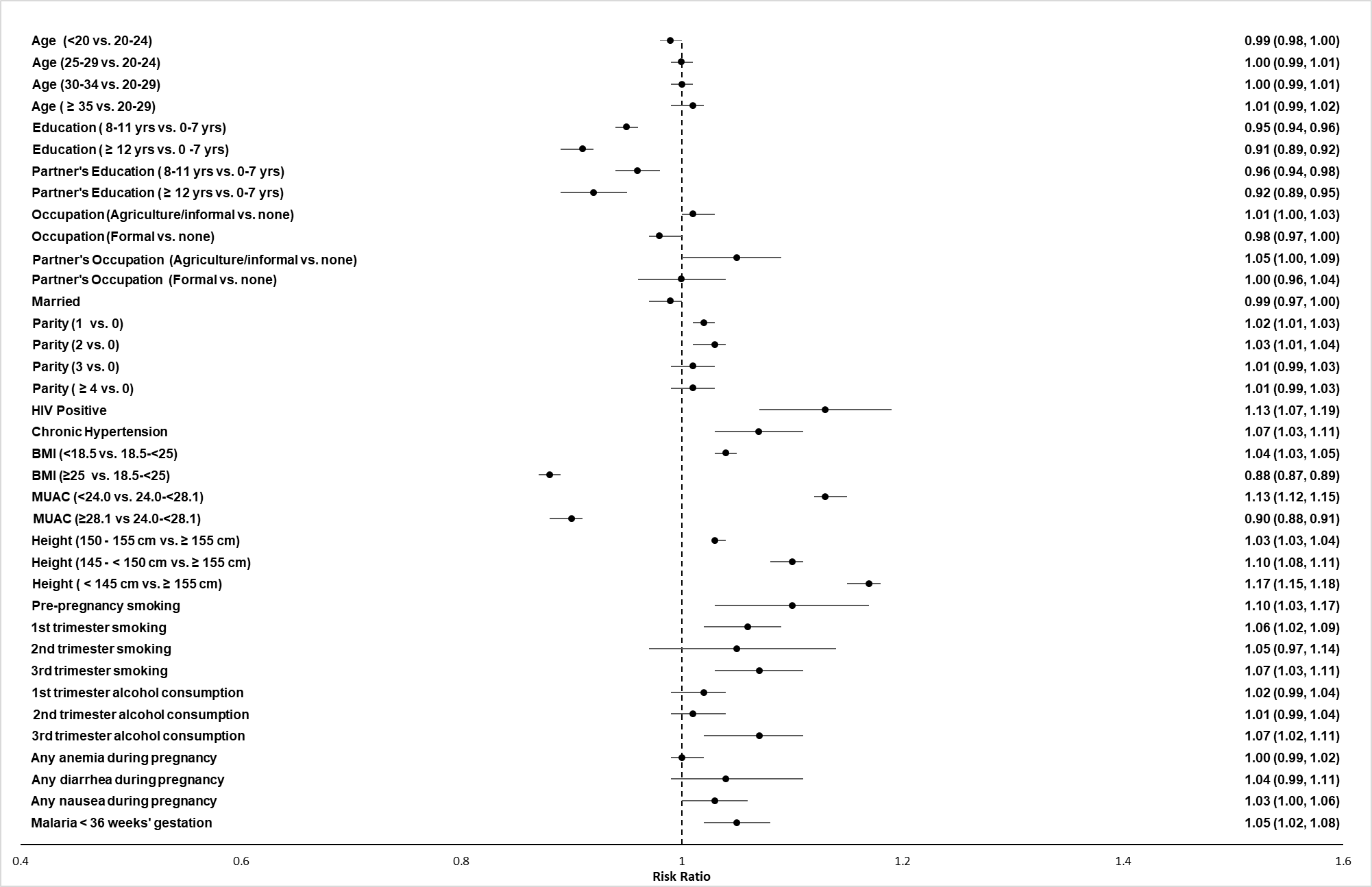


GWG=Gestational weight gain, BMI=body mass index, MUAC=mid-upper arm circumference, HIV=Human immunodeficiency virus, cm=centimeter

**Figure Q1 in S1 Appendix.** Unadjusted risk ratios and 95% confidence intervals for the associations between demongraphic, anthropometric, substance use, and clinical risk factors and inadequate GWG (1-stage model) among women with a third trimester weight measurement (n=69,659). Circles represent risk ratios and bars represent 95% confidence intervals.


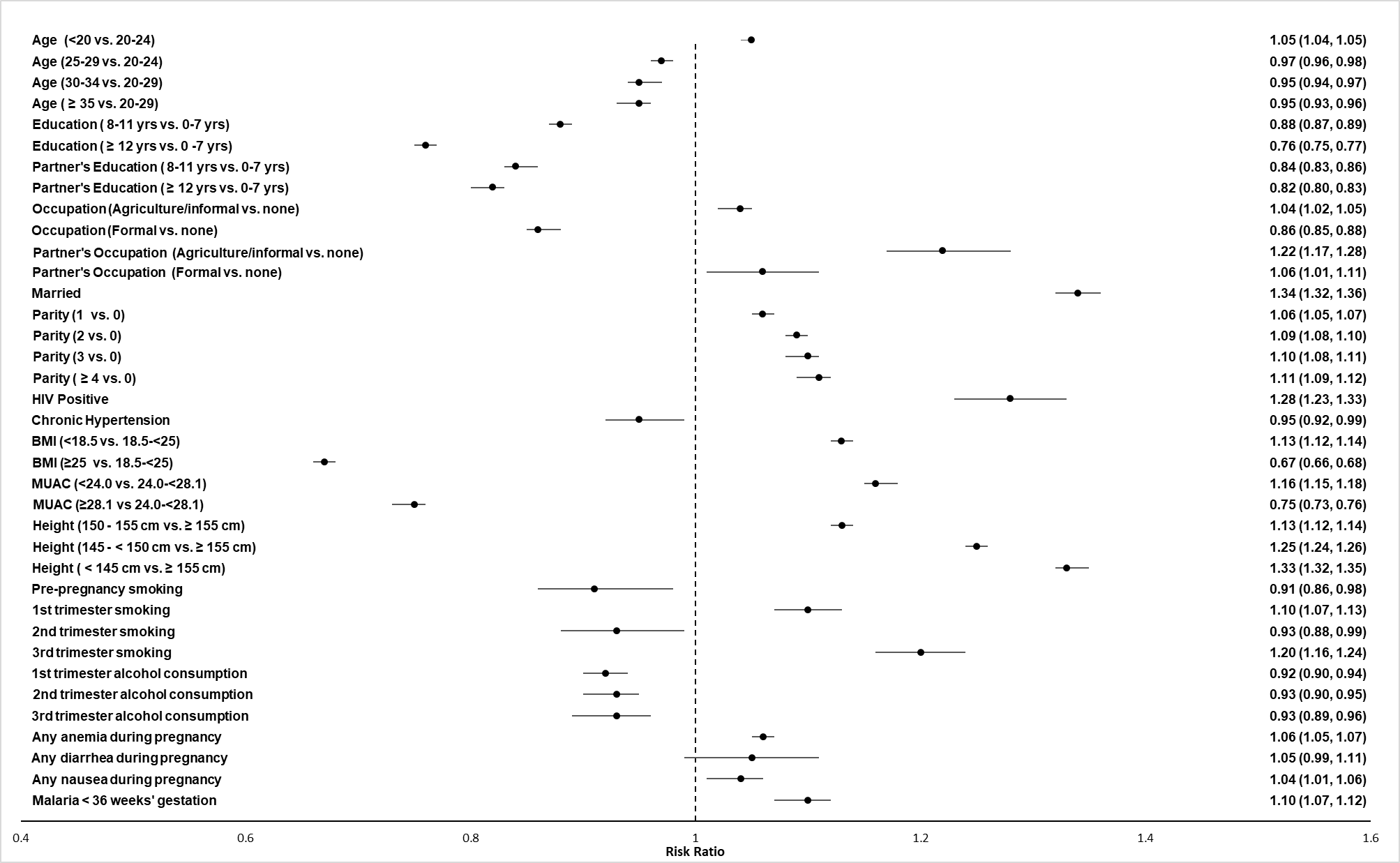


GWG=Gestational weight gain, BMI=body mass index, MUAC=mid-upper arm circumference, HIV=Human immunodeficiency virus, cm=centimeter

**Figure Q2 in S1 Appendix.** Adjusted risk ratios and 95% confidence intervals for the associations between demongraphic, anthropometric, substance use, and clinical risk factors and inadequate GWG (1-stage model) among women with a third trimester weight measurement (n=69,659). Circles represent risk ratios and bars represent 95% confidence intervals.


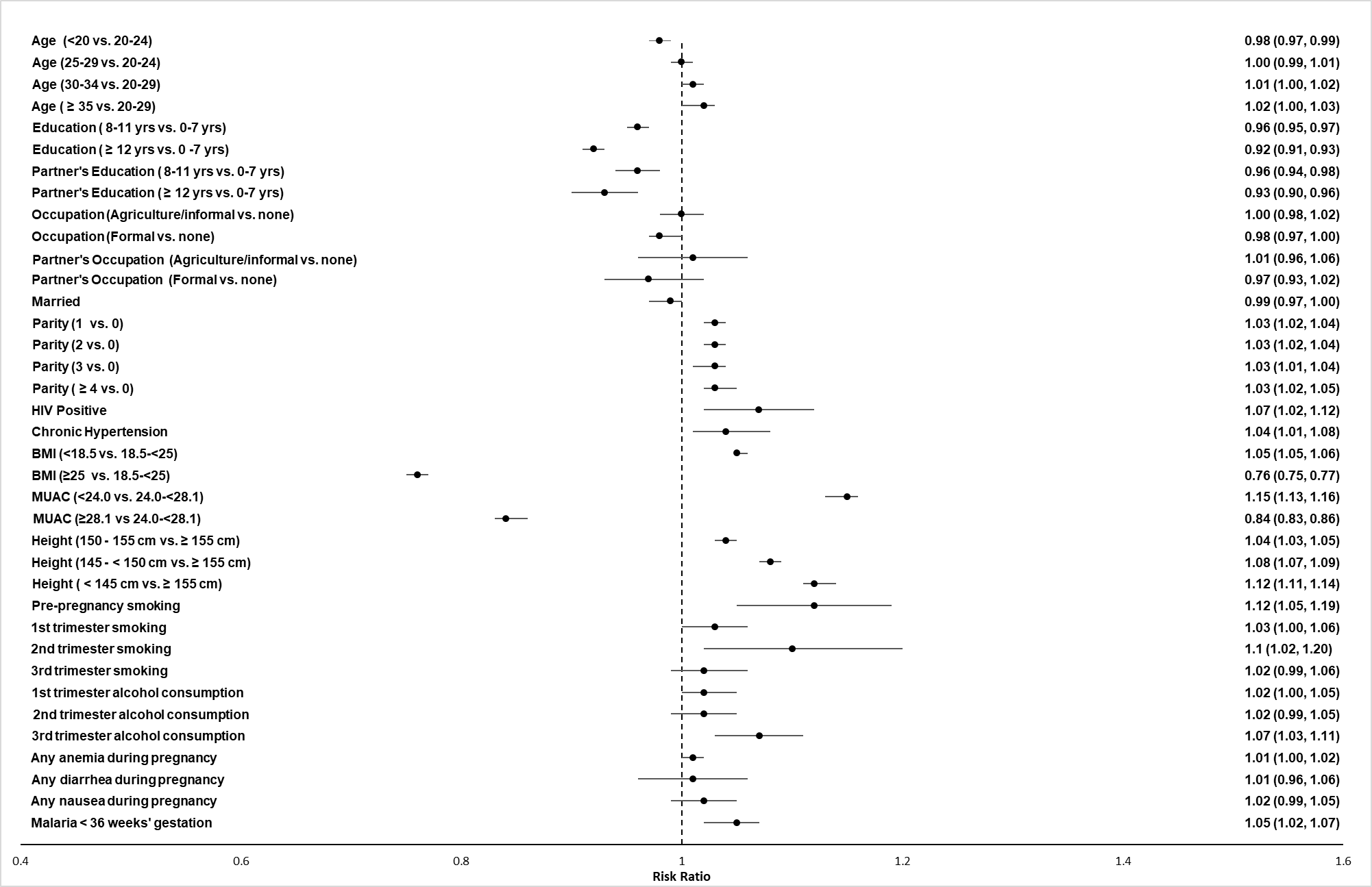


GWG=Gestational weight gain, BMI=body mass index, MUAC=mid-upper arm circumference, HIV=Human immunodeficiency virus, cm=centimeter

**Figure R1 in S1 Appendix.** Unadjusted risk ratios and 95% confidence intervals for the associations between demongraphic, anthropometric, substance use, and clinical risk factors and excessive GWG (1-stage model) among women with a third trimester weight measurement (n=69,659). Circles represent risk ratios and bars represent 95% confidence intervals.


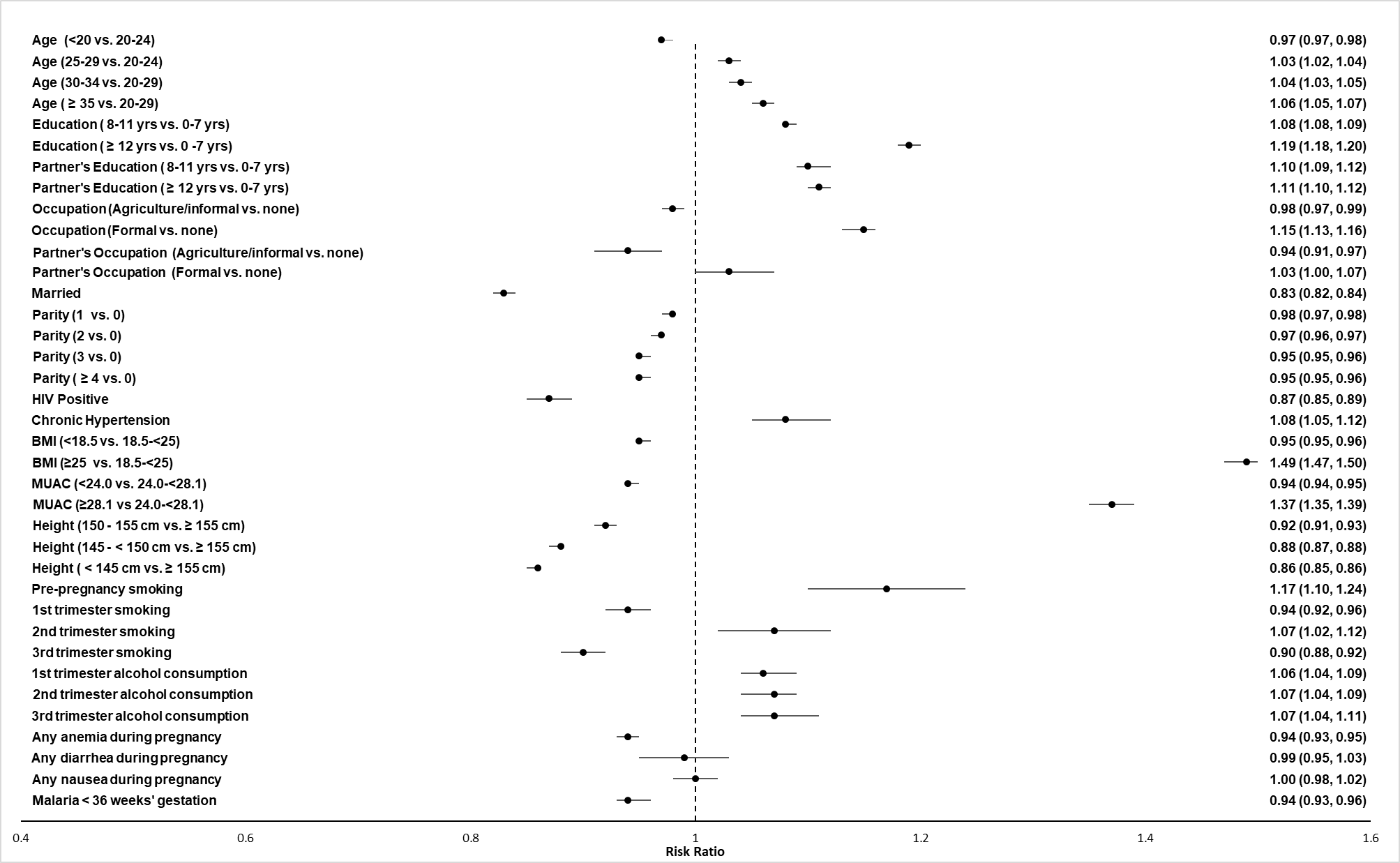


GWG=Gestational weight gain, BMI=body mass index, MUAC=mid-upper arm circumference, HIV=Human immunodeficiency virus, cm=centimeter

**Figure R2 in S1 Appendix.** Adjusted risk ratios and 95% confidence intervals for the associations between demongraphic, anthropometric, substance use, and clinical risk factors and excessive GWG (1-stage model) among women with a third trimester weight measurement (n=69,659). Circles represent risk ratios and bars represent 95% confidence intervals.


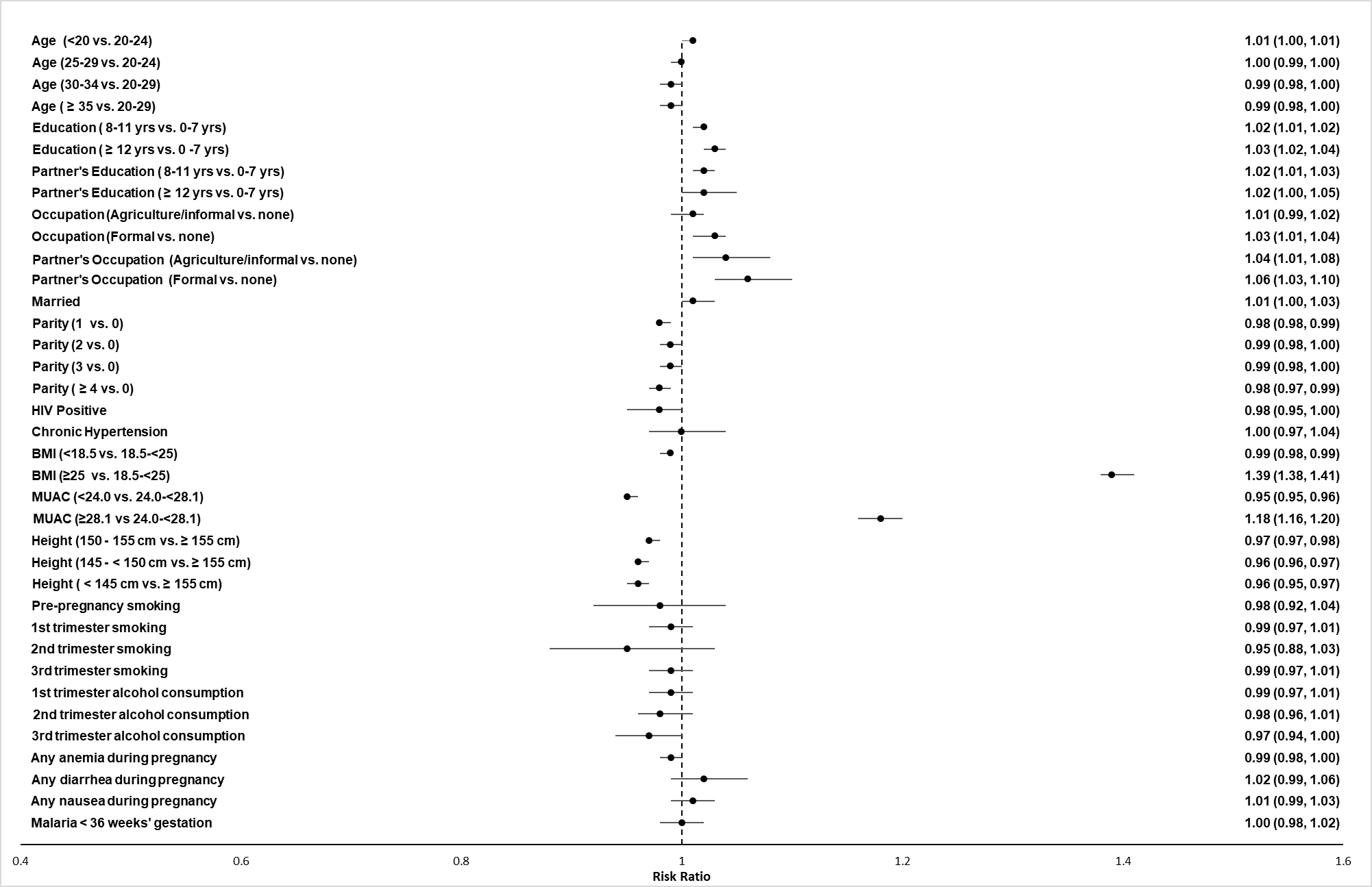


GWG=Gestational weight gain, BMI=body mass index, MUAC=mid-upper arm circumference, HIV=Human immunodeficiency virus, cm=centimeter

**Figure S1 in S1 Appendix.** Unadusted risk ratios and 95% confidence intervals for the associations between demongraphic, anthropometric, substance use, and clinical risk factors and severely inadequate GWG (1-stage model) using the lower limit of the IOM recommendations to calculate expected GWG (n=79,748). Circles represent risk ratios and bars represent 95% confidence intervals.


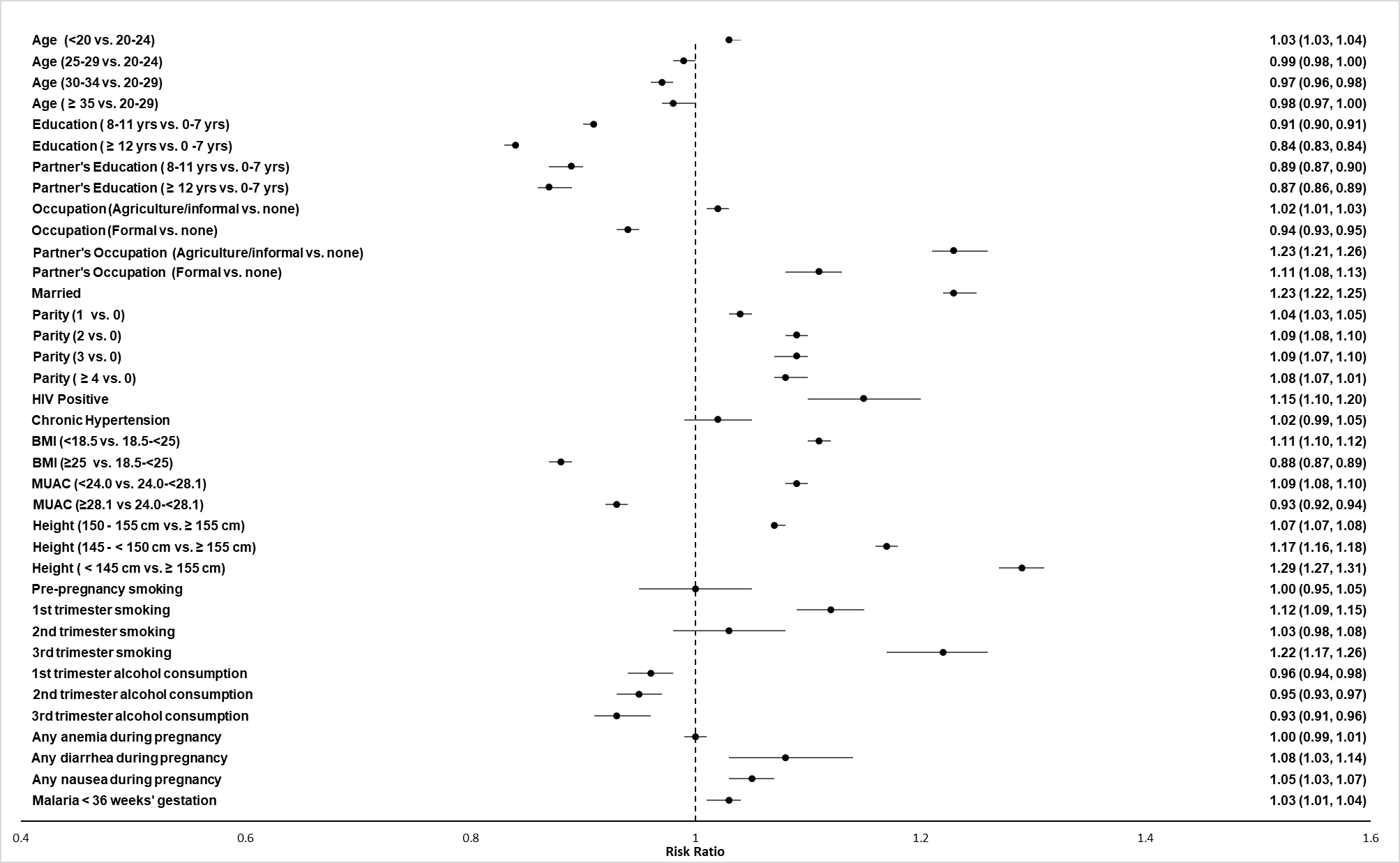


GWG=Gestational weight gain, BMI=body mass index, MUAC=mid-upper arm circumference, HIV=Human immunodeficiency virus, cm=centimeter

**Figure S2 in S1 Appendix.** Adjusted risk ratios and 95% confidence intervals for the associations between demongraphic, anthropometric, substance use, and clinical risk factors and severely inadequate GWG (1-stage model) using the lower limit of the IOM recommendations to calculate expected GWG (n=79,748). Circles represent risk ratios and bars represent 95% confidence intervals.


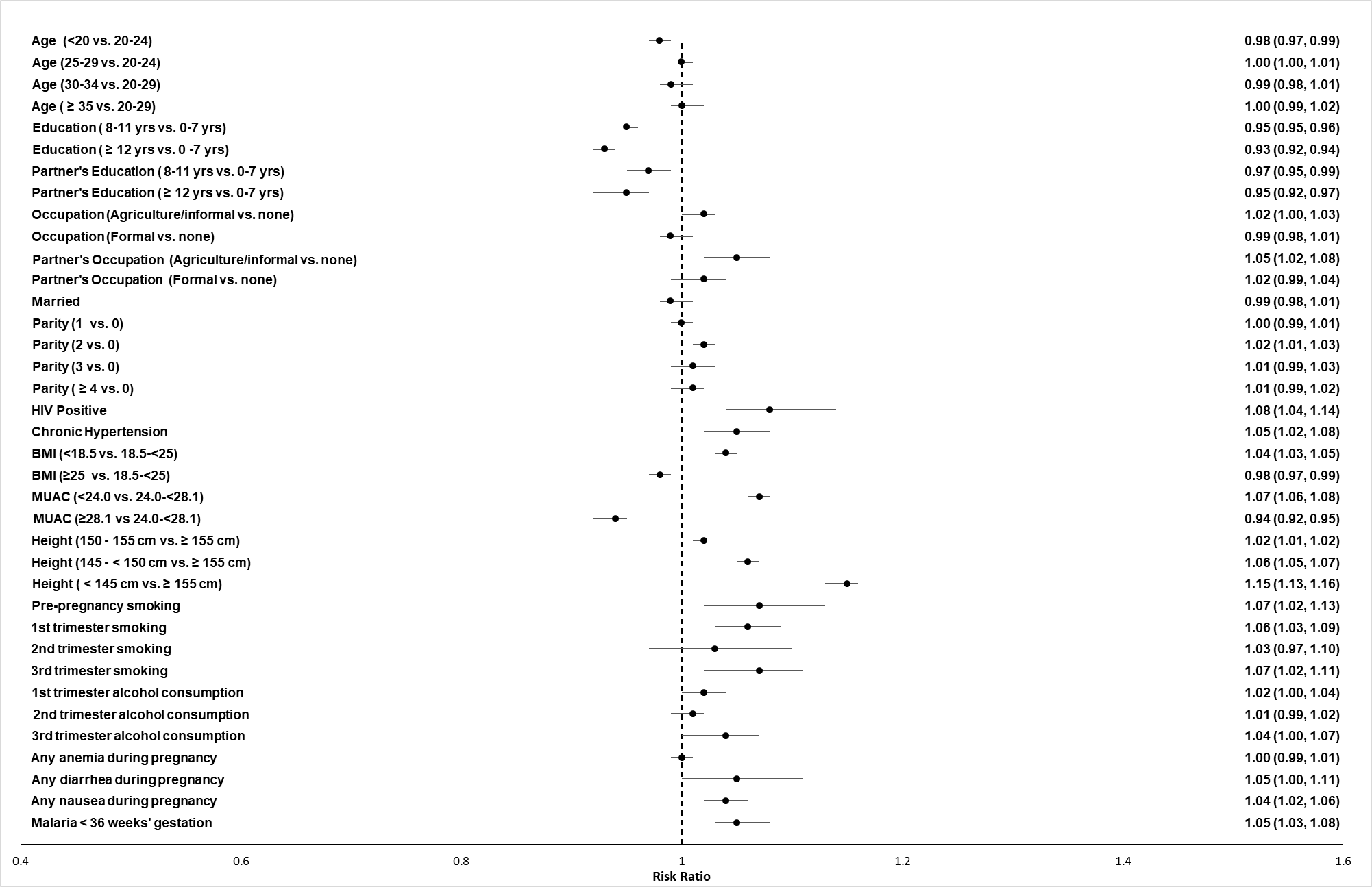


GWG=Gestational weight gain, BMI=body mass index, MUAC=mid-upper arm circumference, HIV=Human immunodeficiency virus, cm=centimeter

**Figure T1 in S1 Appendix.** Adjusted risk ratios and 95% confidence intervals for the associations between demongraphic, anthropometric, substance use, and clinical risk factors and inadequate GWG (1-stage model) using the lower limit of the IOM recommendations to calculate expected GWG (n=79,748). Circles represent risk ratios and bars represent 95% confidence intervals.


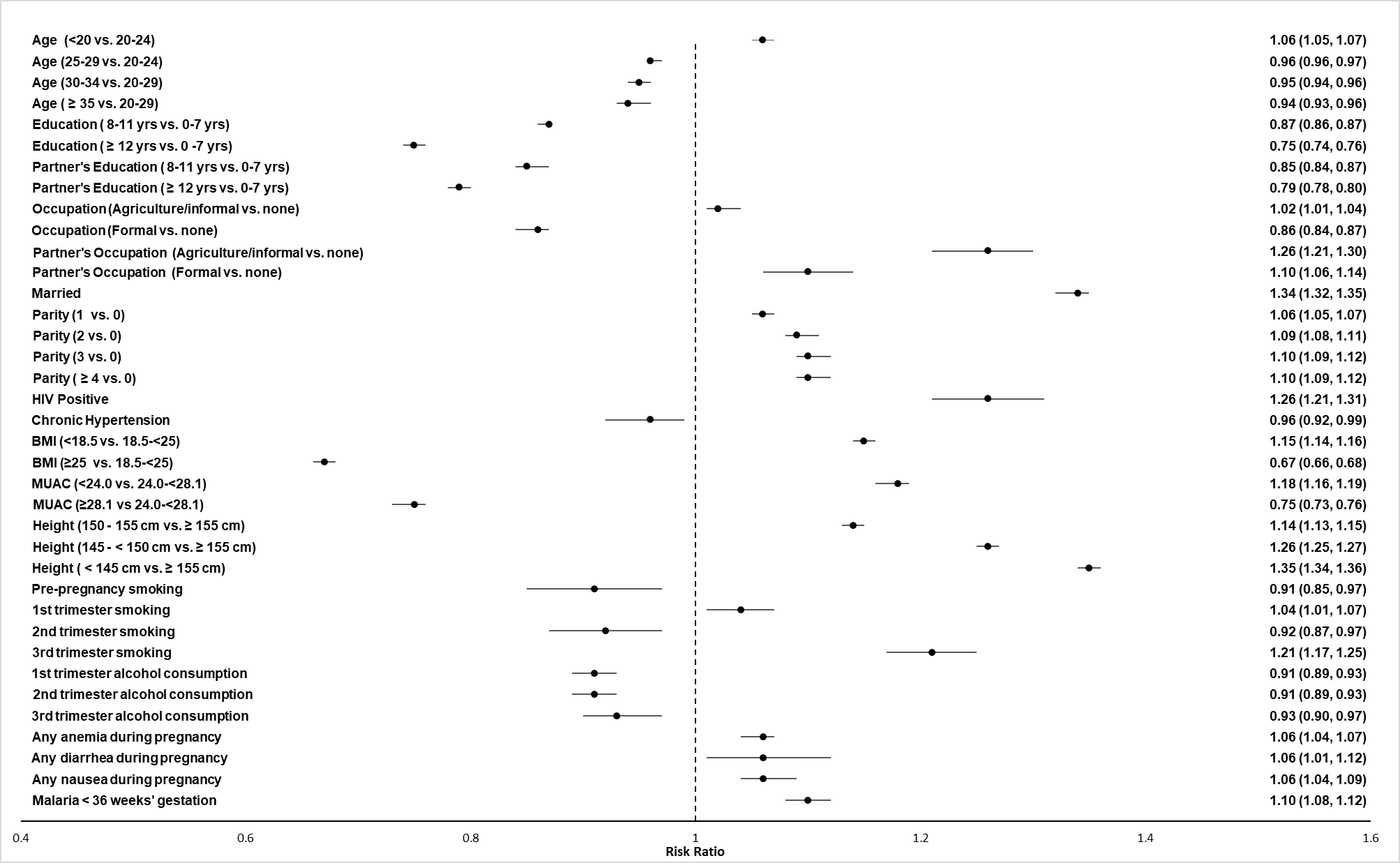


GWG=Gestational weight gain, BMI=body mass index, MUAC=mid-upper arm circumference, HIV=Human immunodeficiency virus, cm=centimeter

**Figure T2 in S1 Appendix.** Unadjusted risk ratios and 95% confidence intervals for the associations between demongraphic, anthropometric, substance use, and clinical risk factors and inadequate GWG (1-stage model) using the lower limit of the IOM recommendations to calculate expected GWG (n=79,748). Circles represent risk ratios and bars represent 95% confidence intervals.


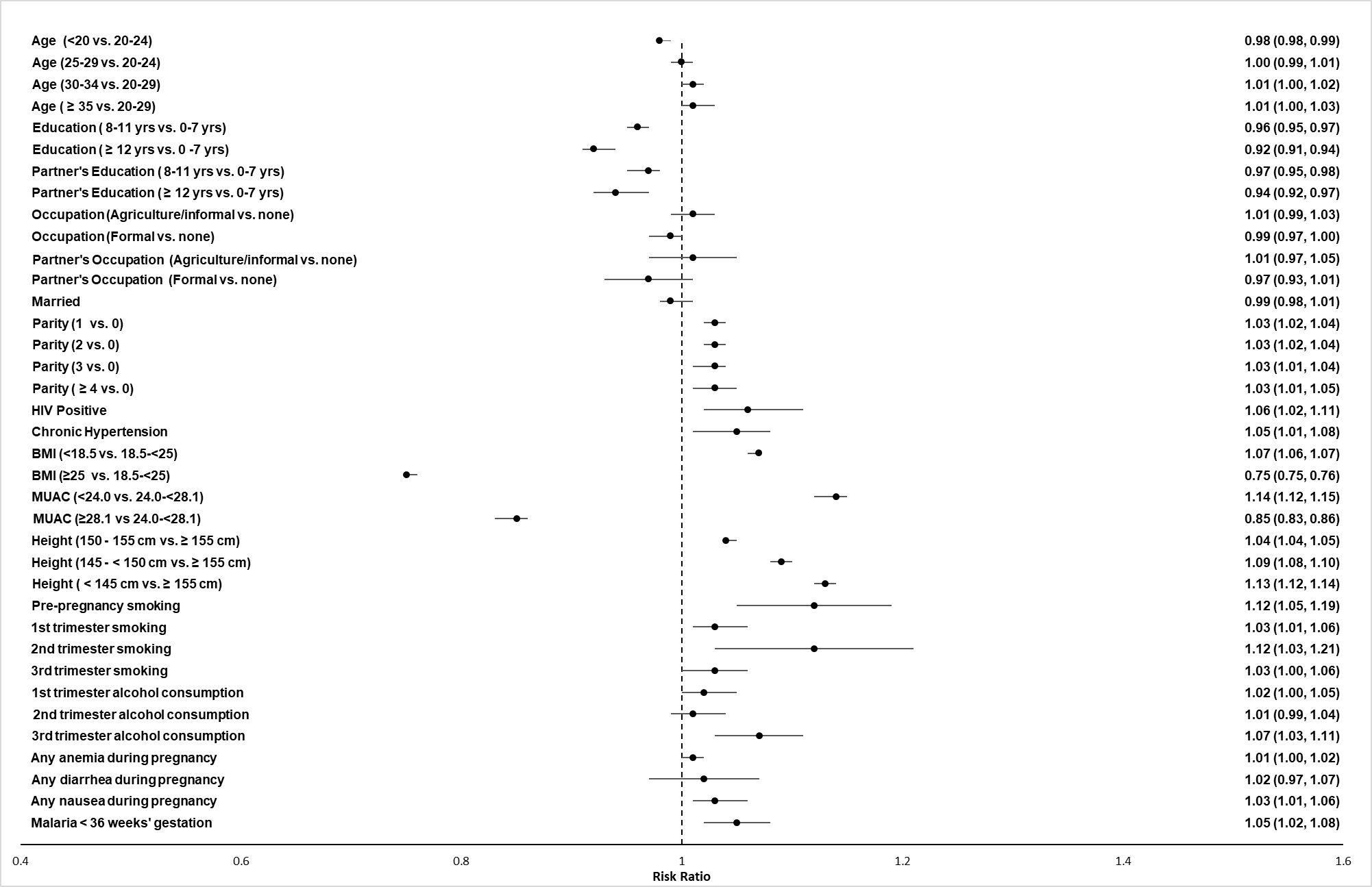


GWG=Gestational weight gain, BMI=body mass index, MUAC=mid-upper arm circumference, HIV=Human immunodeficiency virus, cm=centimeter

**Figure U1 in S1 Appendix.** Unadjusted risk ratios and 95% confidence intervals for the associations between demongraphic, anthropometric, substance use, and clinical risk factors and excessive GWG (1-stage model) using the lower limit of the IOM recommendations to calculate expected GWG (n=79,748). Circles represent risk ratios and bars represent 95% confidence intervals.


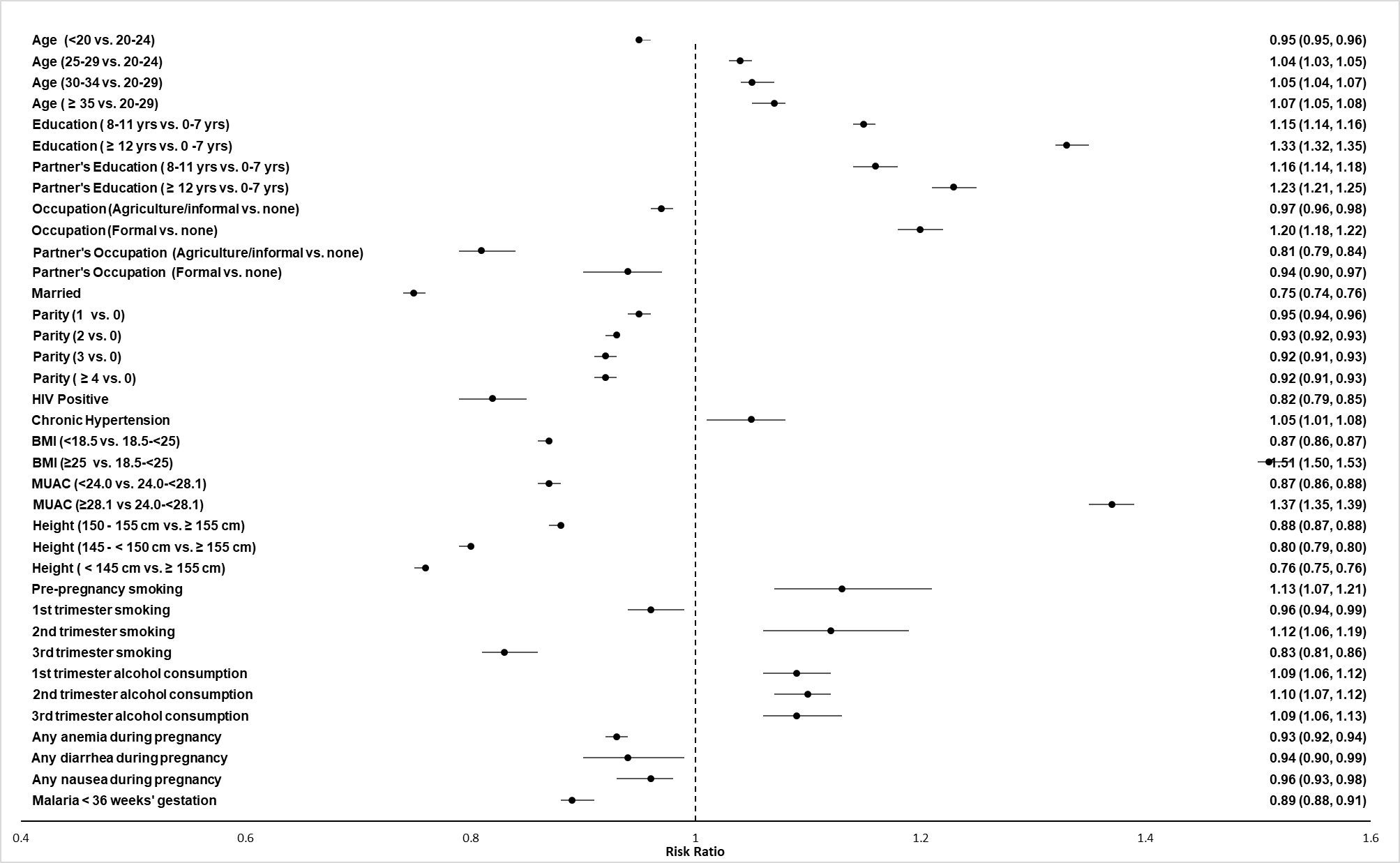


GWG=Gestational weight gain, BMI=body mass index, MUAC=mid-upper arm circumference, HIV=Human immunodeficiency virus, cm=centimeter

**Figure U2 in S1 Appendix.** Adjusted risk ratios and 95% confidence intervals for the associations between demongraphic, anthropometric, substance use, and clinical risk factors and excessive GWG (1-stage model) using the lower limit of the IOM recommendations to calculate expected GWG (n-79,748). Circles represent risk ratios and bars represent 95% confidence intervals.


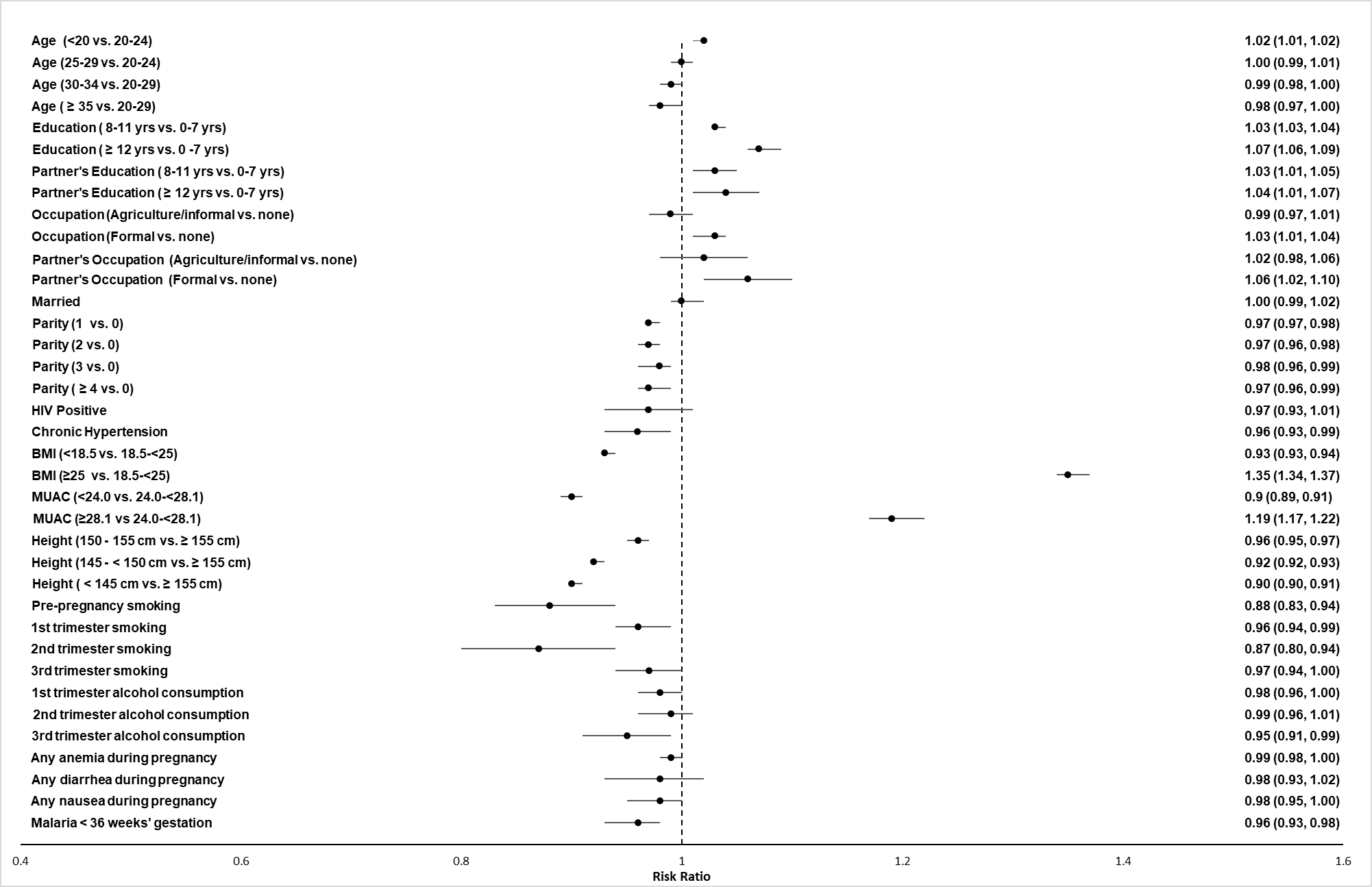


GWG=Gestational weight gain, BMI=body mass index, MUAC=mid-upper arm circumference, HIV=Human immunodeficiency virus, cm=centimeter

**Figure V1 in S1 Appendix.** Unadjusted risk ratios and 95% confidence intervals for the associations between demongraphic, anthropometric, substance use, and clinical risk factors and severely inadequate GWG (2-stage model) using an Asia-specific body mass index cutoff to define overweight/obesity for Asian participants (n=138,286). Circles represent risk ratios and bars represent 95% confidence intervals.


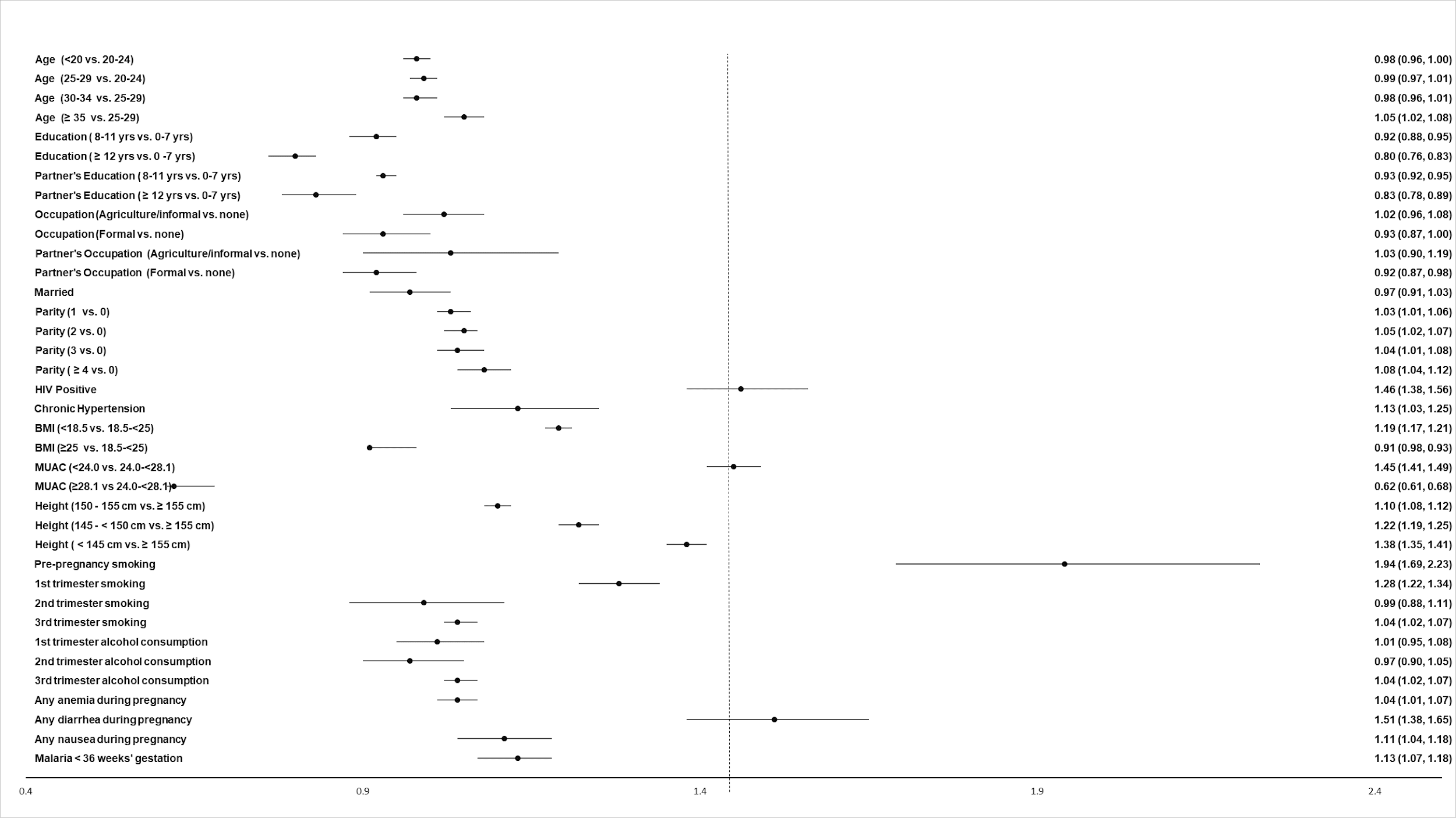


GWG=Gestational weight gain, BMI=body mass index, MUAC=mid-upper arm circumference, HIV=Human immunodeficiency virus, cm=centimeter

**Figure V2 in S1 Appendix.** Adjusted risk ratios and 95% confidence intervals for the associations between demongraphic, anthropometric, substance use, and clinical risk factors and severely inadequate GWG (2-stage model) using an Asia-specific body mass index cutoff to define overweight/obesity for Asian participants (n=138,286). Circles represent risk ratios and bars represent 95% confidence intervals.


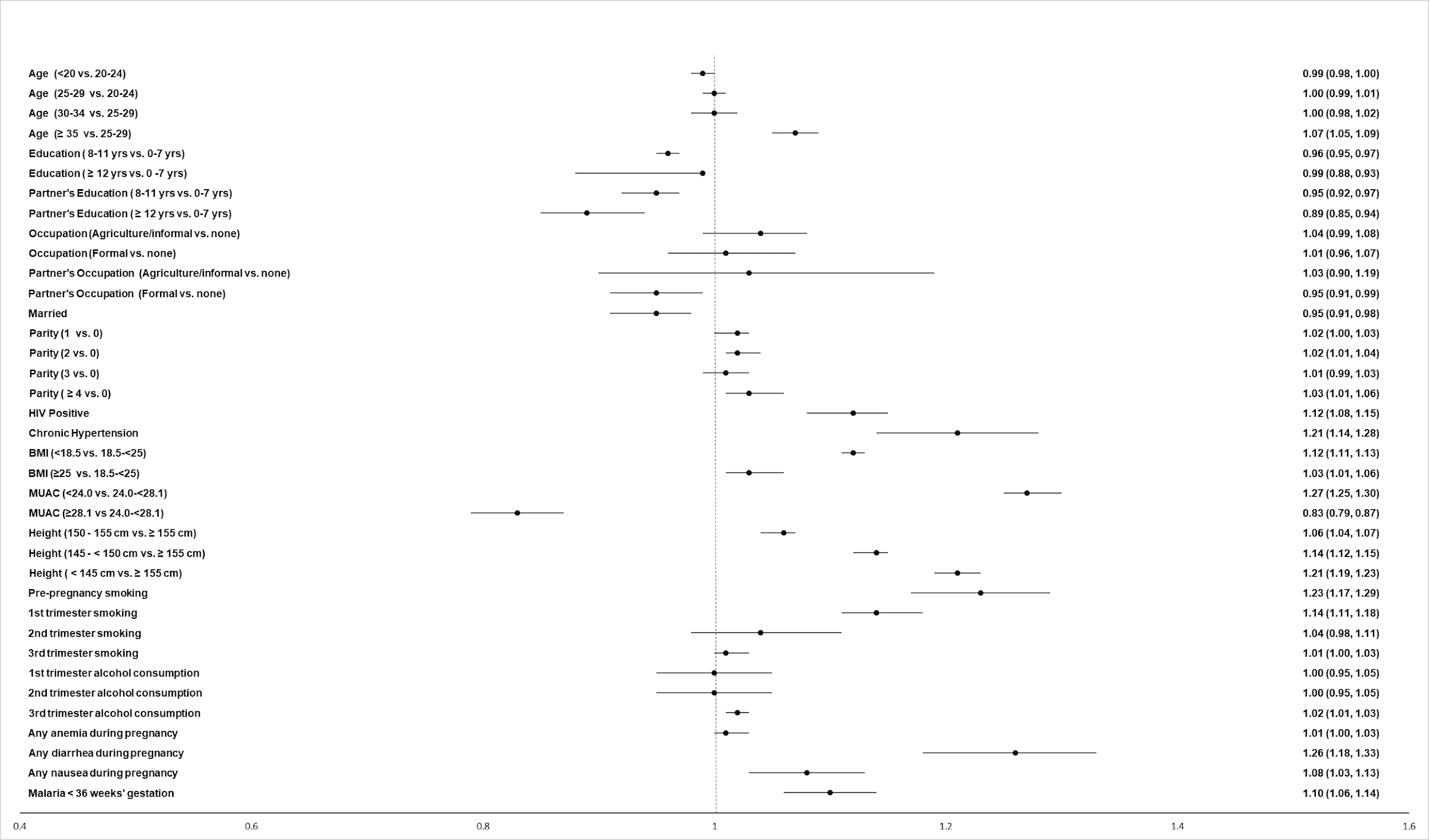


GWG=Gestational weight gain, BMI=body mass index, MUAC=mid-upper arm circumference, HIV=Human immunodeficiency virus, cm=centimeter

**Figure W1 in S1 Appendix.** Unadjusted risk ratios and 95% confidence intervals for the associations between demongraphic, anthropometric, substance use, and clinical risk factors and inadequate GWG (2-stage model) using an Asia-specific body mass index cutoff to define overweight/obesity for Asian participants (n=138,286). Circles represent risk ratios and bars represent 95% confidence intervals.


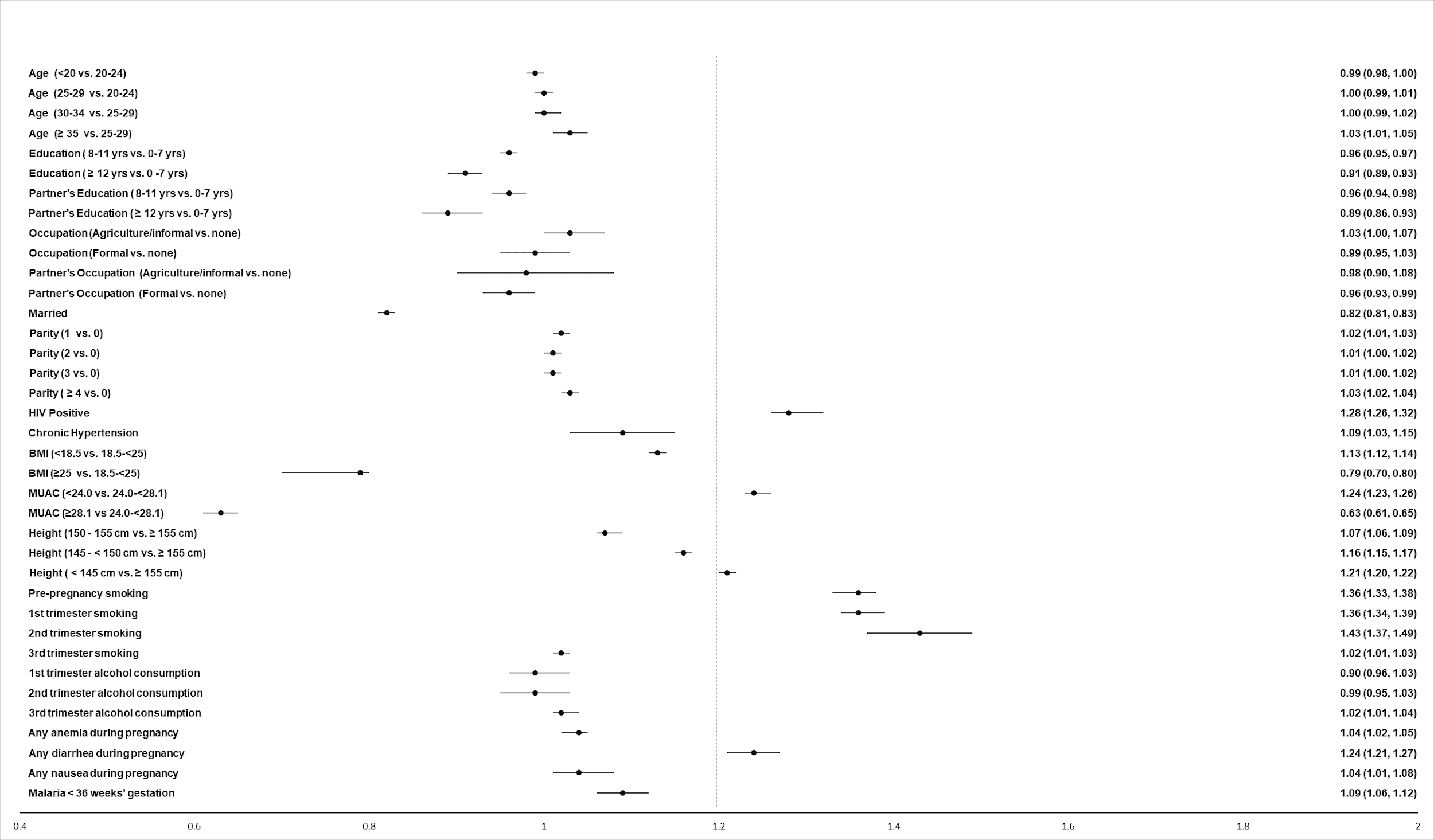


GWG=Gestational weight gain, BMI=body mass index, MUAC=mid-upper arm circumference, HIV=Human immunodeficiency virus, cm=centimeter

**Figure W2 in S1 Appendix.** Adjusted risk ratios and 95% confidence intervals for the associations between demongraphic, anthropometric, substance use, and clinical risk factors and inadequate GWG (2-stage model) using an Asia-specific body mass index cutoff to define overweight/obesity for Asian participants (n=138,286). Circles represent risk ratios and bars represent 95% confidence intervals.


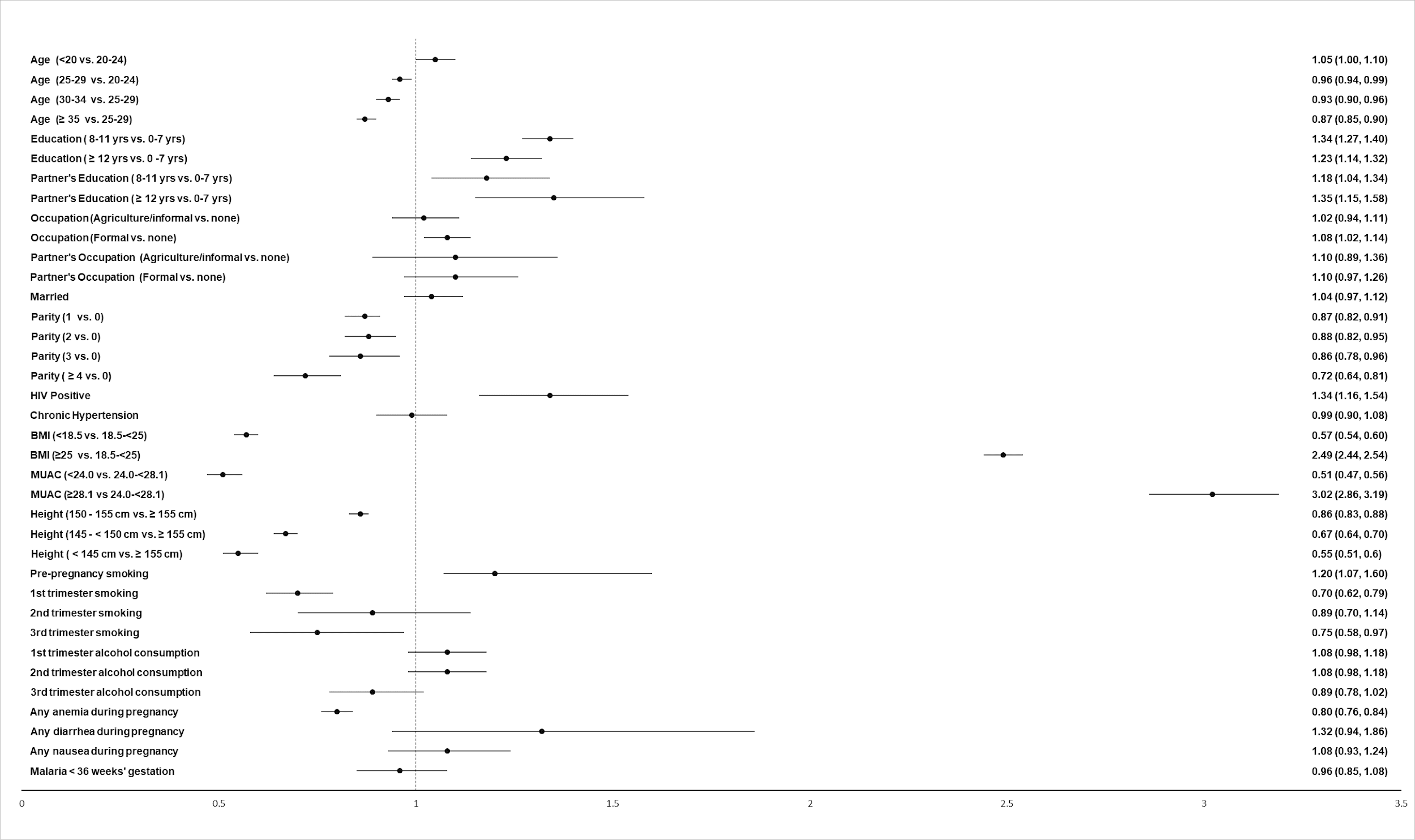


GWG=Gestational weight gain, BMI=body mass index, MUAC=mid-upper arm circumference, HIV=Human immunodeficiency virus, cm=centimeter

**Figure X1 in S1 Appendix.** Unadjusted risk ratios and 95% confidence intervals for the associations between demongraphic, anthropometric, substance use, and clinical risk factors and excessive GWG (2-stage model) using an Asia-specific body mass index cutoff to define overweight/obesity for Asian participants (n=138,286). Circles represent risk ratios and bars represent 95% confidence intervals.


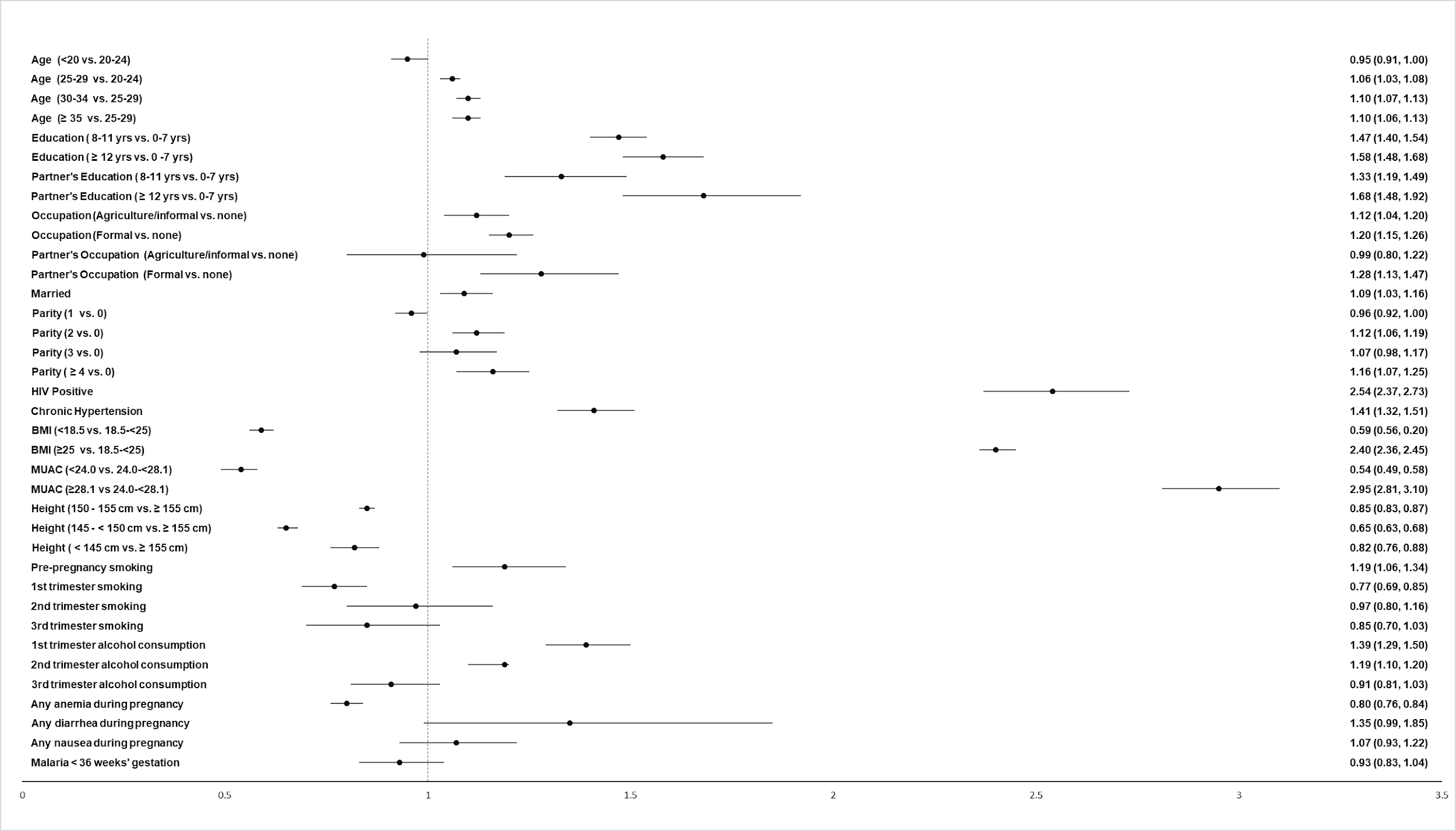


GWG=Gestational weight gain, BMI=body mass index, MUAC=mid-upper arm circumference, HIV=Human immunodeficiency virus, cm=centimeter

**Figure X2 in S1 Appendix.** Adjusted risk ratios and 95% confidence intervals for the associations between demongraphic, anthropometric, substance use, and clinical risk factors and excessive GWG (2-stage model) using an Asia-specific body mass index cutoff to define overweight/obesity for Asian participants (n=138,286). Circles represent risk ratios and bars represent 95% confidence intervals.


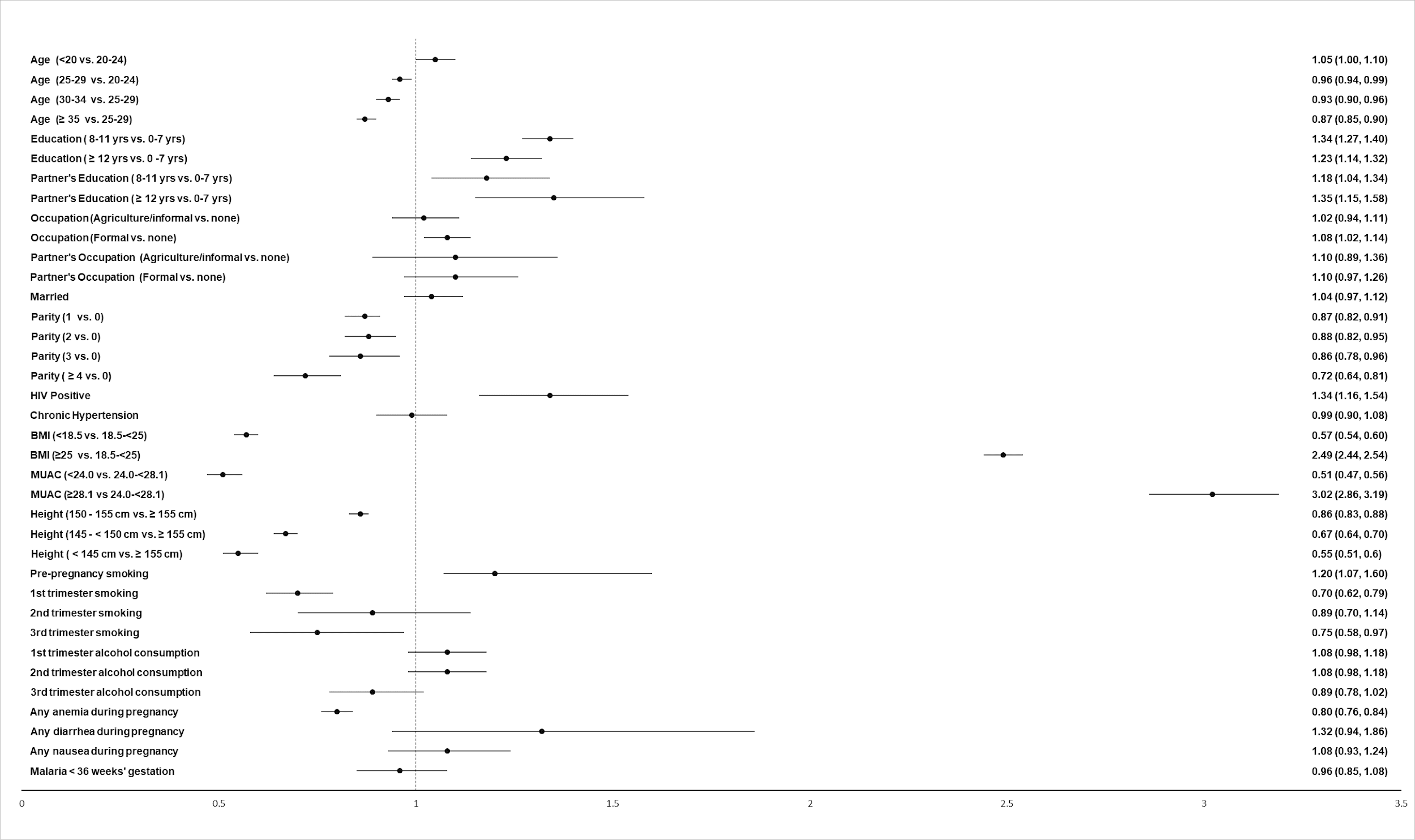


GWG=Gestational weight gain, BMI=body mass index, MUAC=mid-upper arm circumference, HIV=Human immunodeficiency virus, cm=centimeter

**Figure Y1 in S1 Appendix.** Unadjusted risk ratios and 95% confidence intervals for the associations between demongraphic, anthropometric, substance use, and clinical risk factors and severely inadequate GWG (2-stage model) using those with adequate GWG as the reference category (n=138,286). Circles represent risk ratios and bars represent 95% confidence intervals.


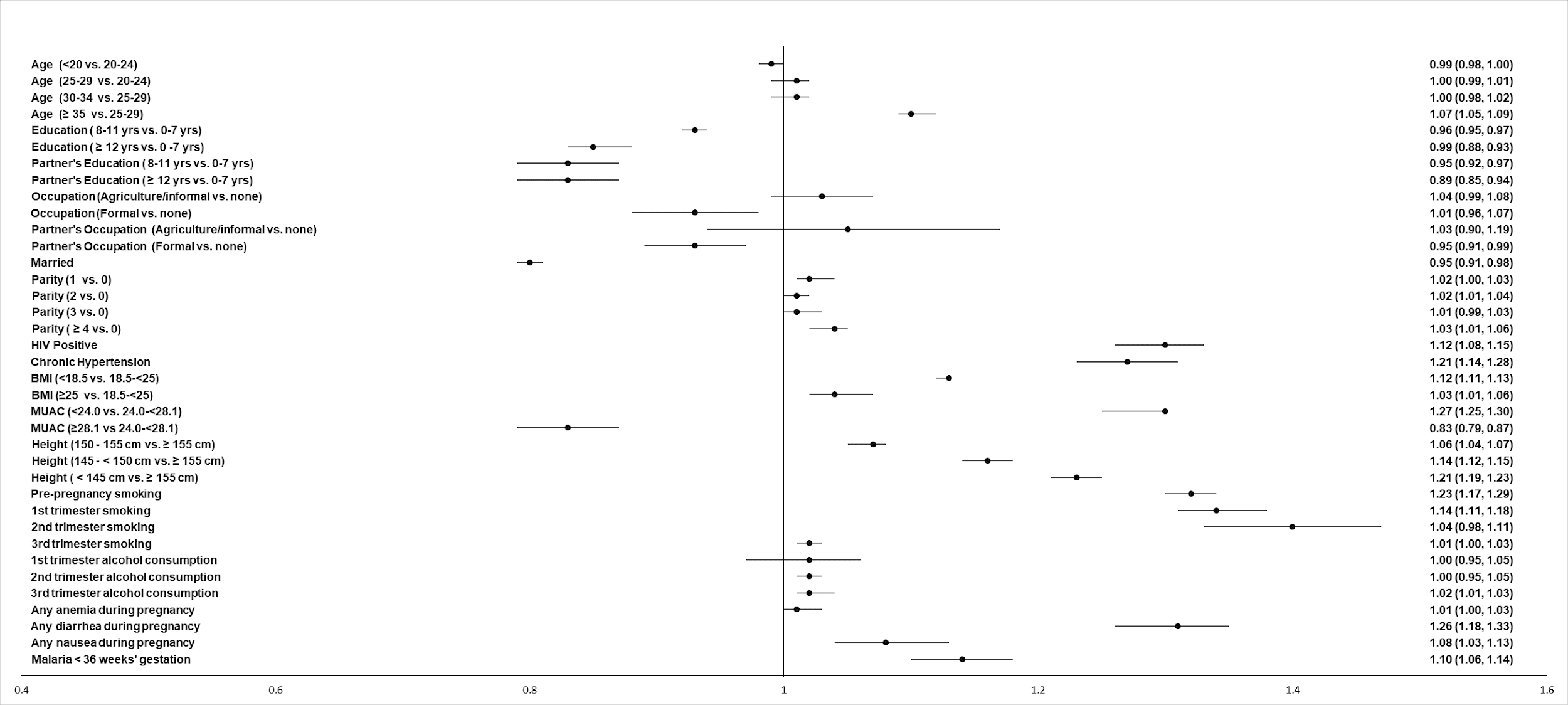


GWG=Gestational weight gain, BMI=body mass index, MUAC=mid-upper arm circumference, HIV=Human immunodeficiency virus, cm=centimeter

**Figure Y2 in S1 Appendix.** Adjusted risk ratios and 95% confidence intervals for the associations between demongraphic, anthropometric, substance use, and clinical risk factors and severely inadequate GWG (2-stage model) using those with adequate GWG as the reference category (138,286). Circles represent risk ratios and bars represent 95% confidence intervals.


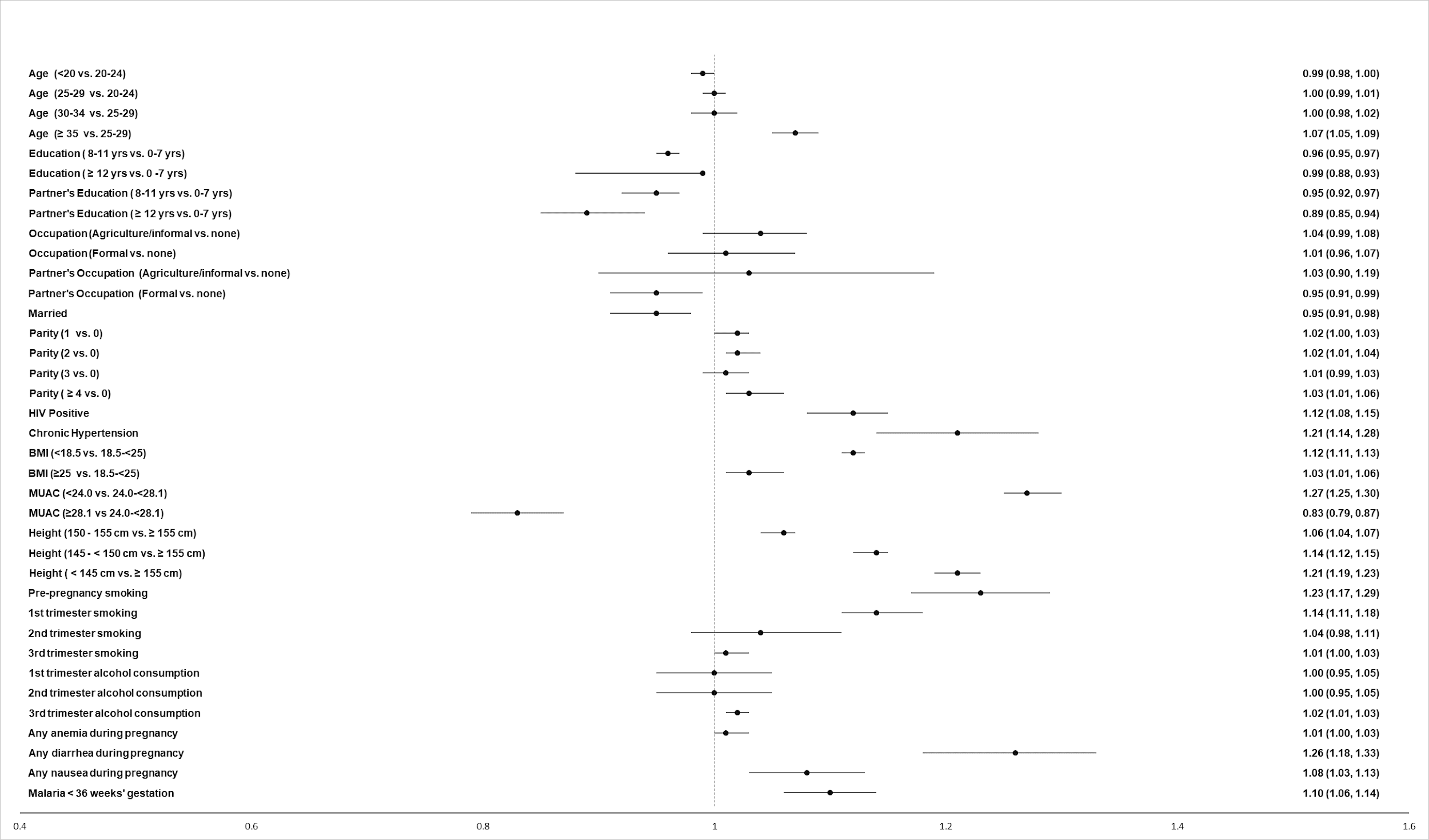


GWG=Gestational weight gain, BMI=body mass index, MUAC=mid-upper arm circumference, HIV=Human immunodeficiency virus, cm=centimeter

**Figure Z1 in S1 Appendix.** Unadjusted risk ratios and 95% confidence intervals for the associations between demongraphic, anthropometric, substance use, and clinical risk factors and inadequate GWG (2-stage model) using those with adequate GWG as the reference category (n=138,286). Circles represent risk ratios and bars represent 95% confidence intervals.


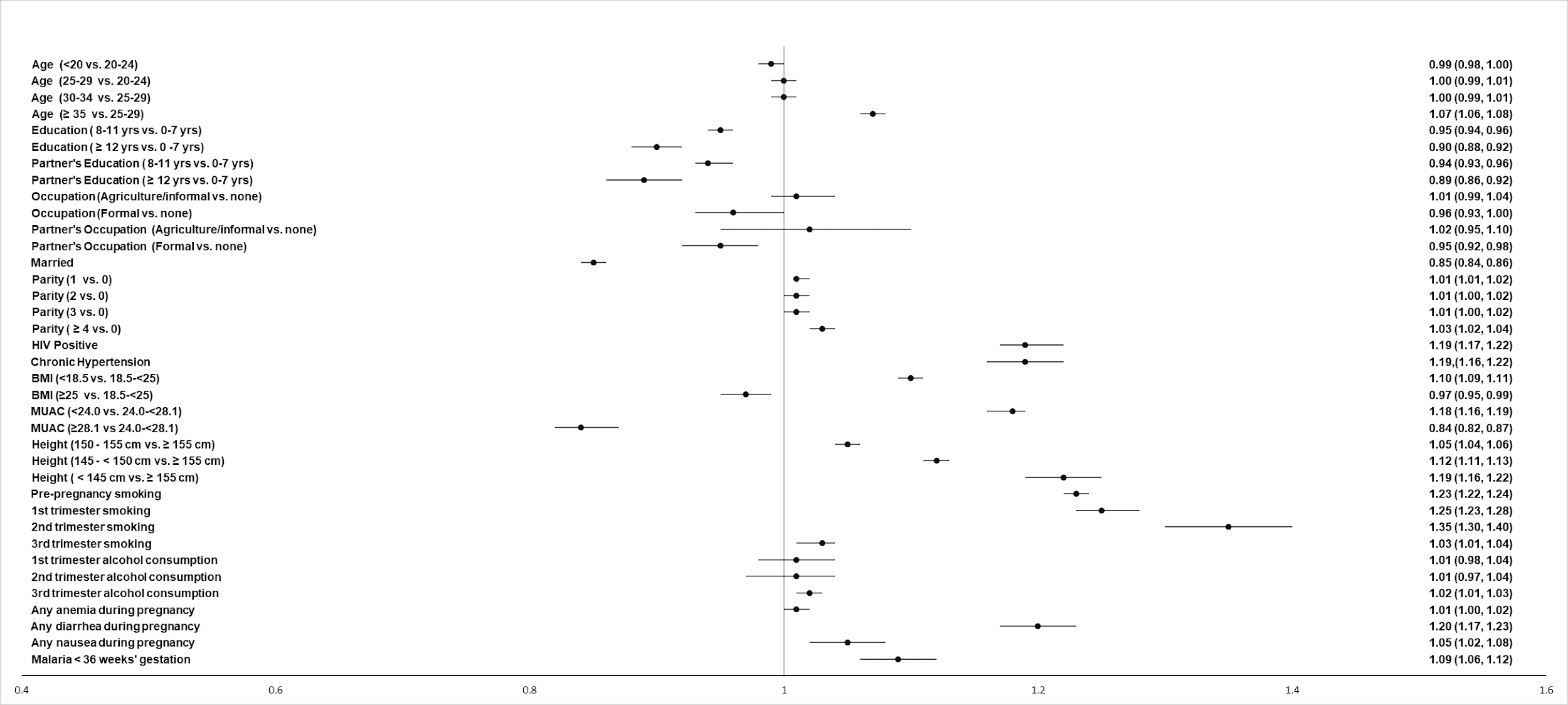


GWG=Gestational weight gain, BMI=body mass index, MUAC=mid-upper arm circumference, HIV=Human immunodeficiency virus, cm=centimeter

**Figure Z2 in S1 Appendix.** Adjusted risk ratios and 95% confidence intervals for the associations between demongraphic, anthropometric, substance use, and clinical risk factors and inadequate GWG (2-stage model) using those with adequate GWG as the reference category (n=138,286). Circles represent risk ratios and bars represent 95% confidence intervals.


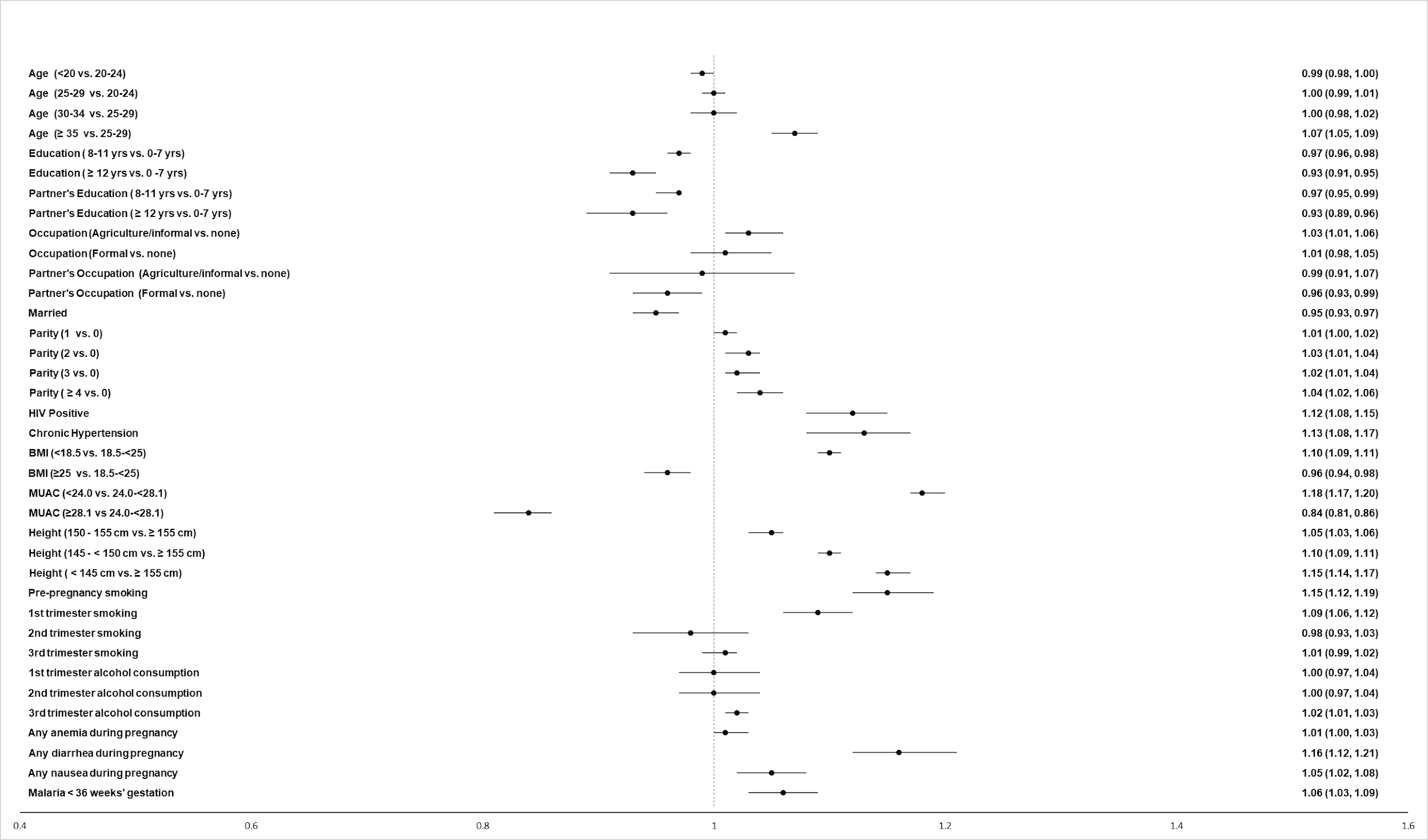


GWG=Gestational weight gain, BMI=body mass index, MUAC=mid-upper arm circumference, HIV=Human immunodeficiency virus, cm=centimeter

**Figure AA1 in S1 Appendix.** Unadjusted risk ratios and 95% confidence intervals for the associations between demongraphic, anthropometric, substance use, and clinical risk factors and excessive GWG (2-stage model) using those with adequate GWG as the reference category (n=138,286). Circles represent risk ratios and bars represent 95% confidence intervals.


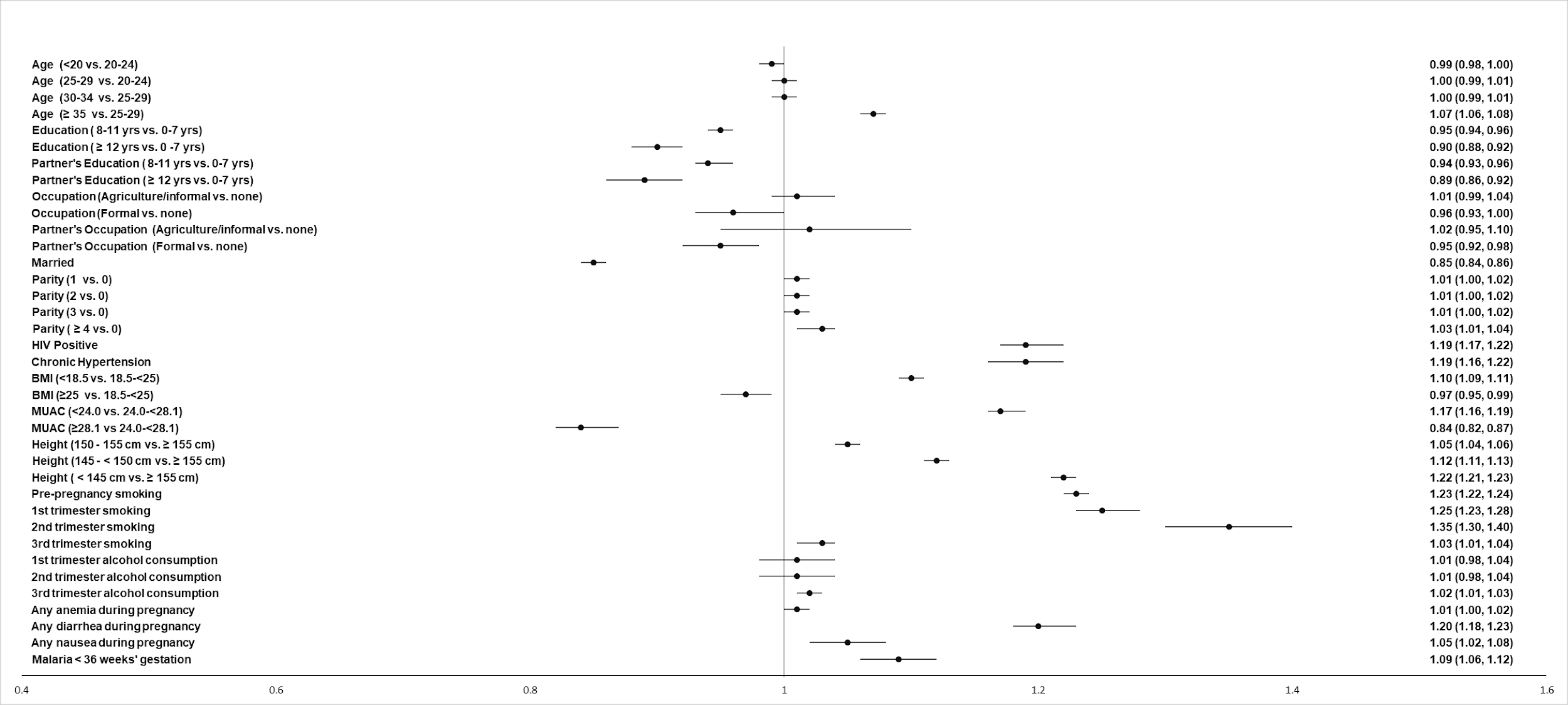


GWG=Gestational weight gain, BMI=body mass index, MUAC=mid-upper arm circumference, HIV=Human immunodeficiency virus, cm=centimeter

**Figure AA2 in S1 Appendix.** Adjusted risk ratios and 95% confidence intervals for the associations between demongraphic, anthropometric, substance use, and clinical risk factors and excessive GWG (2-stage model) using those with adequate GWG as the reference category (n=138,286). Circles represent risk ratios and bars represent 95% confidence intervals.


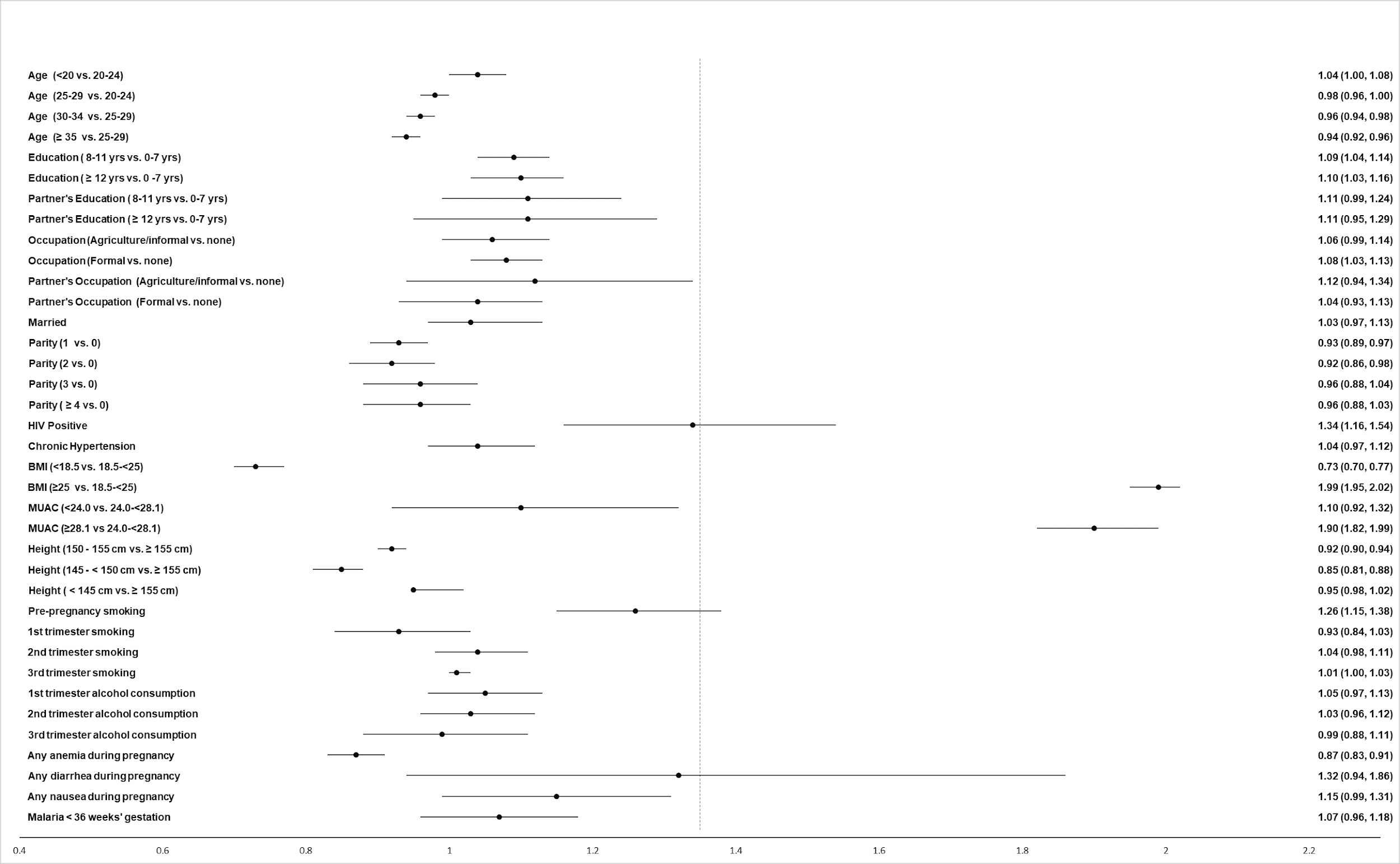


GWG=Gestational weight gain, BMI=body mass index, MUAC=mid-upper arm circumference, HIV=Human immunodeficiency virus, cm=centimeter

**Figure BB1 in S1 Appendix.** Unadjusted risk ratios and 95% confidence intervals for the associations between demongraphic, anthropometric, substance use, and clinical risk factors and severely inadequate GWG (1-stage model) among those who did not receive randomized nutritional interventions. Circles represent risk ratios and bars represent 95% confidence intervals (n=45,389). GWG=Gestational weight gain, BMI=body mass index, MUAC=mid-upper arm circumference, HIV=Human immunodeficiency virus, cm=centimeter


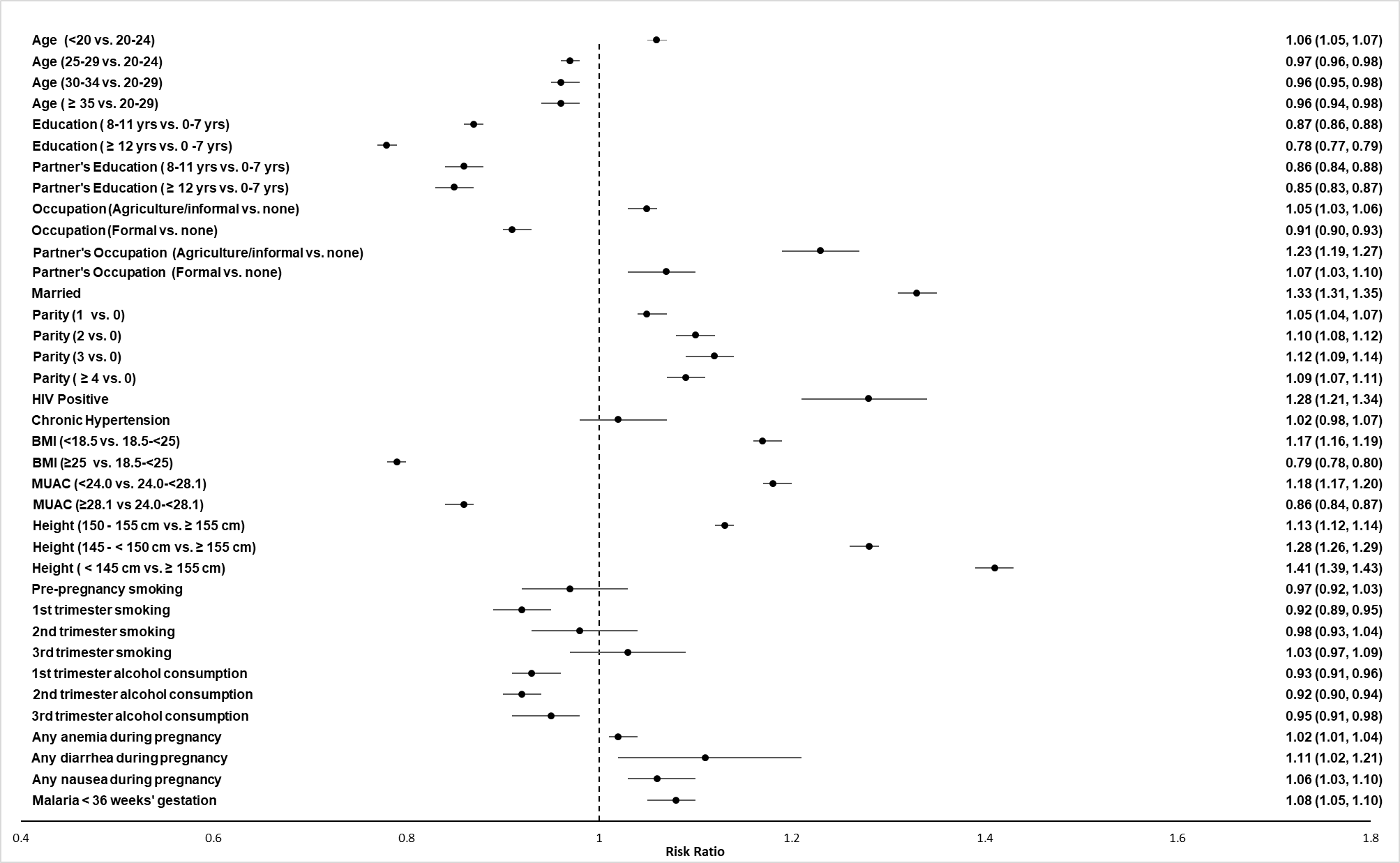


GWG=Gestational weight gain, BMI=body mass index, MUAC=mid-upper arm circumference, HIV=Human immunodeficiency virus, cm=centimeter

**Figure BB2 in S1 Appendix.** Adjusted risk ratios and 95% confidence intervals for the associations between demongraphic, anthropometric, substance use, and clinical risk factors and severely inadequate GWG (1-stage model) among those who did not receive randomized nutritional interventions. Circles represent risk ratios and bars represent 95% confidence intervals (n=45,389). GWG=Gestational weight gain, BMI=body mass index, MUAC=mid-upper arm circumference, HIV=Human immunodeficiency virus, cm=centimeter


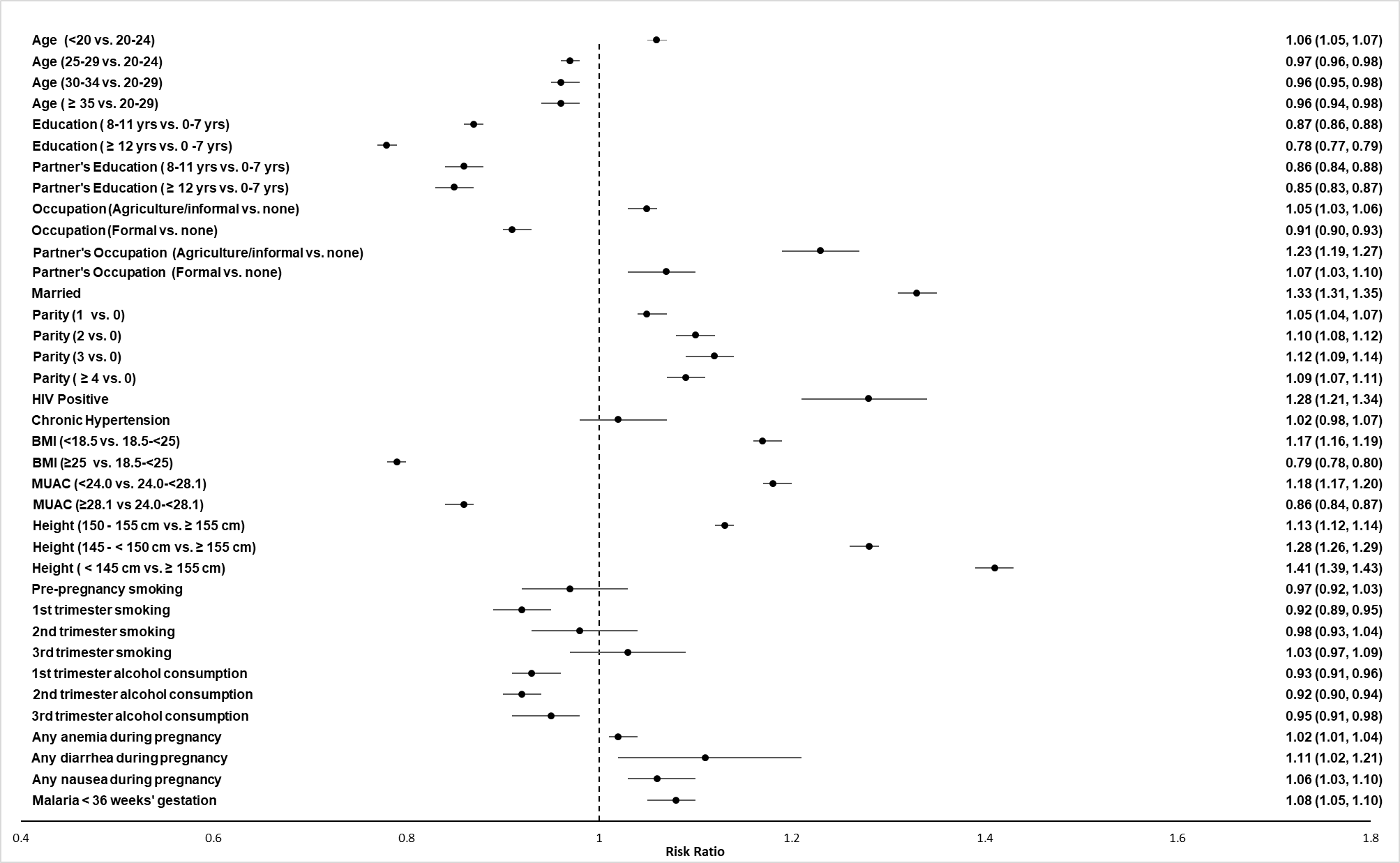


GWG=Gestational weight gain, BMI=body mass index, MUAC=mid-upper arm circumference, HIV=Human immunodeficiency virus, cm=centimeter

**Appendix Figure CC1.** Unadjusted risk ratios and 95% confidence intervals for the associations between demongraphic, anthropometric, substance use, and clinical risk factors and inadequate GWG (1-stage model) among those who did not receive randomized nutritional interventions. Circles represent risk ratios and bars represent 95% confidence intervals (n=45,389). GWG=Gestational weight gain, BMI=body mass index, MUAC=mid-upper arm circumference, HIV=Human immunodeficiency virus, cm=centimeter


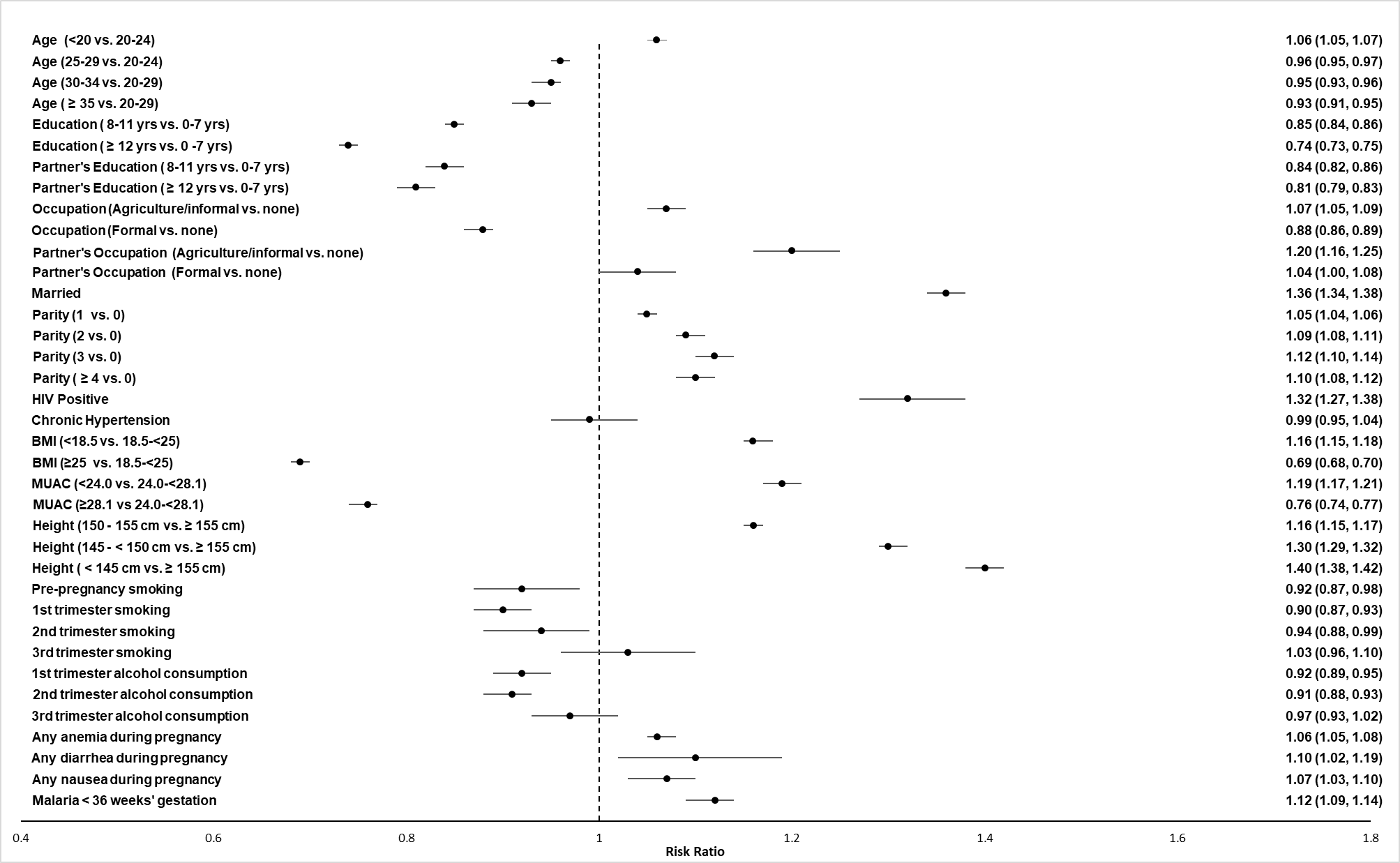


GWG=Gestational weight gain, BMI=body mass index, MUAC=mid-upper arm circumference, HIV=Human immunodeficiency virus, cm=centimeter

**Figure CC2 in S1 Appendix.** Adjusted risk ratios and 95% confidence intervals for the associations between demongraphic, anthropometric, substance use, and clinical risk factors and inadequate GWG (1-stage model) among those who did not receive randomized nutritional interventions. Circles represent risk ratios and bars represent 95% confidence intervals (n=45,389). GWG=Gestational weight gain, BMI=body mass index, MUAC=mid-upper arm circumference, HIV=Human immunodeficiency virus, cm=centimeter


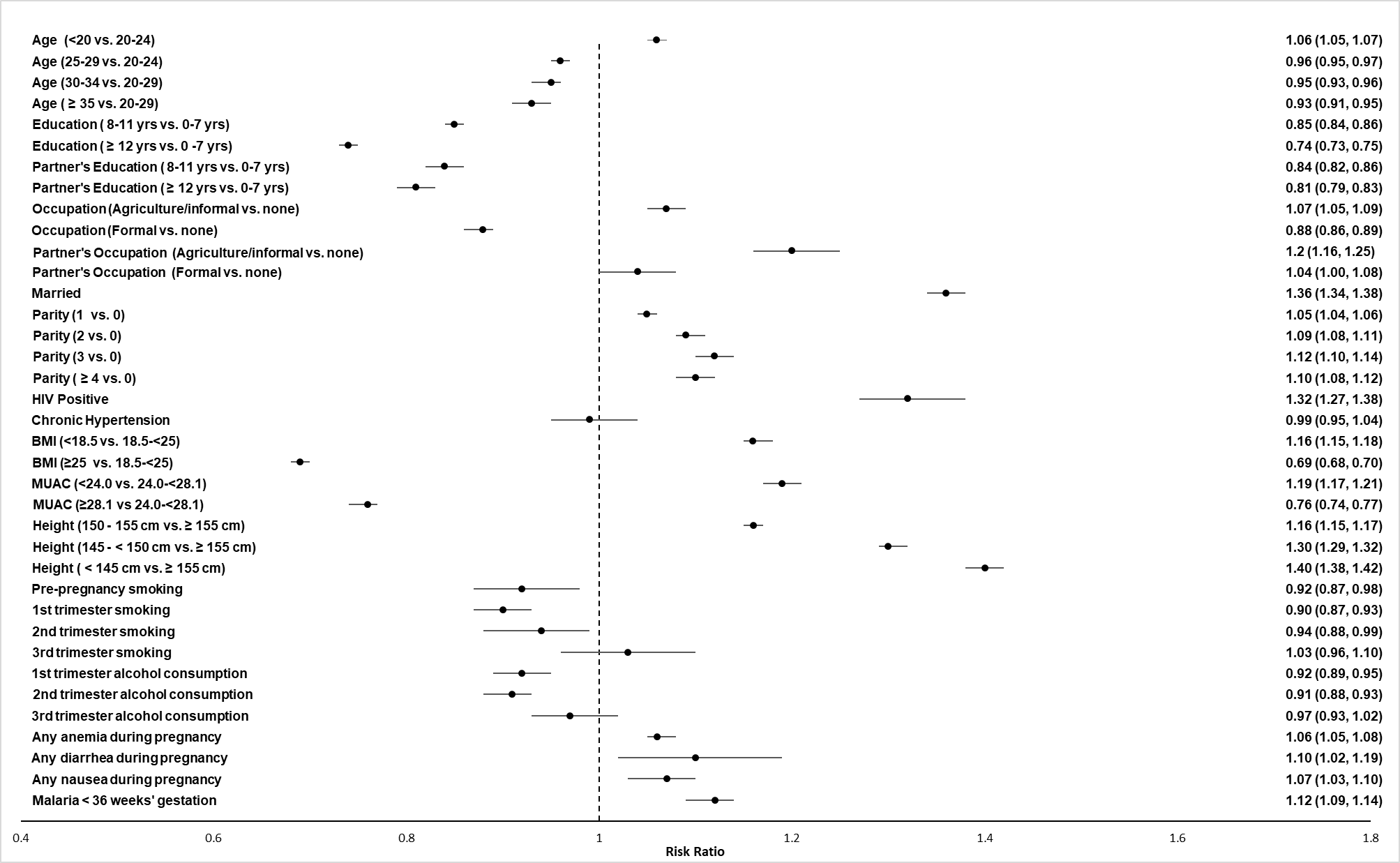


GWG=Gestational weight gain, BMI=body mass index, MUAC=mid-upper arm circumference, HIV=Human immunodeficiency virus, cm=centimeter

**Figure DD1 in S1 Appendix.** Unadjusted risk ratios and 95% confidence intervals for the associations between demongraphic, anthropometric, substance use, and clinical risk factors and excessive GWG (1-stage model) among those who did not receive randomized nutritional interventions. Circles represent risk ratios and bars represent 95% confidence intervals (n=45,389). GWG=Gestational weight gain, BMI=body mass index, MUAC=mid-upper arm circumference, HIV=Human immunodeficiency virus, cm=centimeter


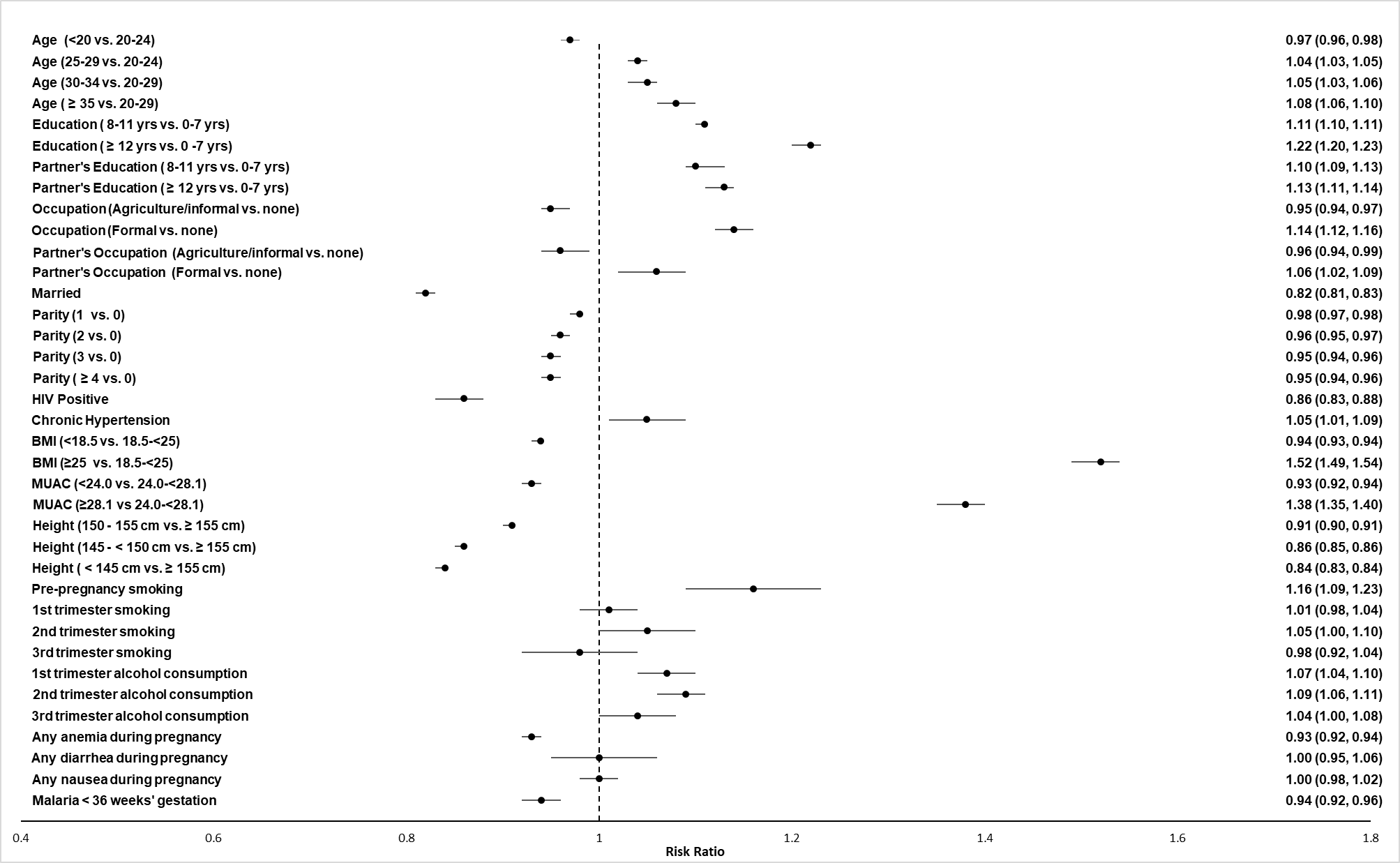


GWG=Gestational weight gain, BMI=body mass index, MUAC=mid-upper arm circumference, HIV=Human immunodeficiency virus, cm=centimeter

**Figure DD2 in S1 Appendix.** Adjusted risk ratios and 95% confidence intervals for the associations between demongraphic, anthropometric, substance use, and clinical risk factors and excessive GWG (1-stage model) among those who did not receive randomized nutritional interventions. Circles represent risk ratios and bars represent 95% confidence intervals. (n=45,389). GWG=Gestational weight gain, BMI=body mass index, MUAC=mid-upper arm circumference, HIV=Human immunodeficiency virus, cm=centimeter


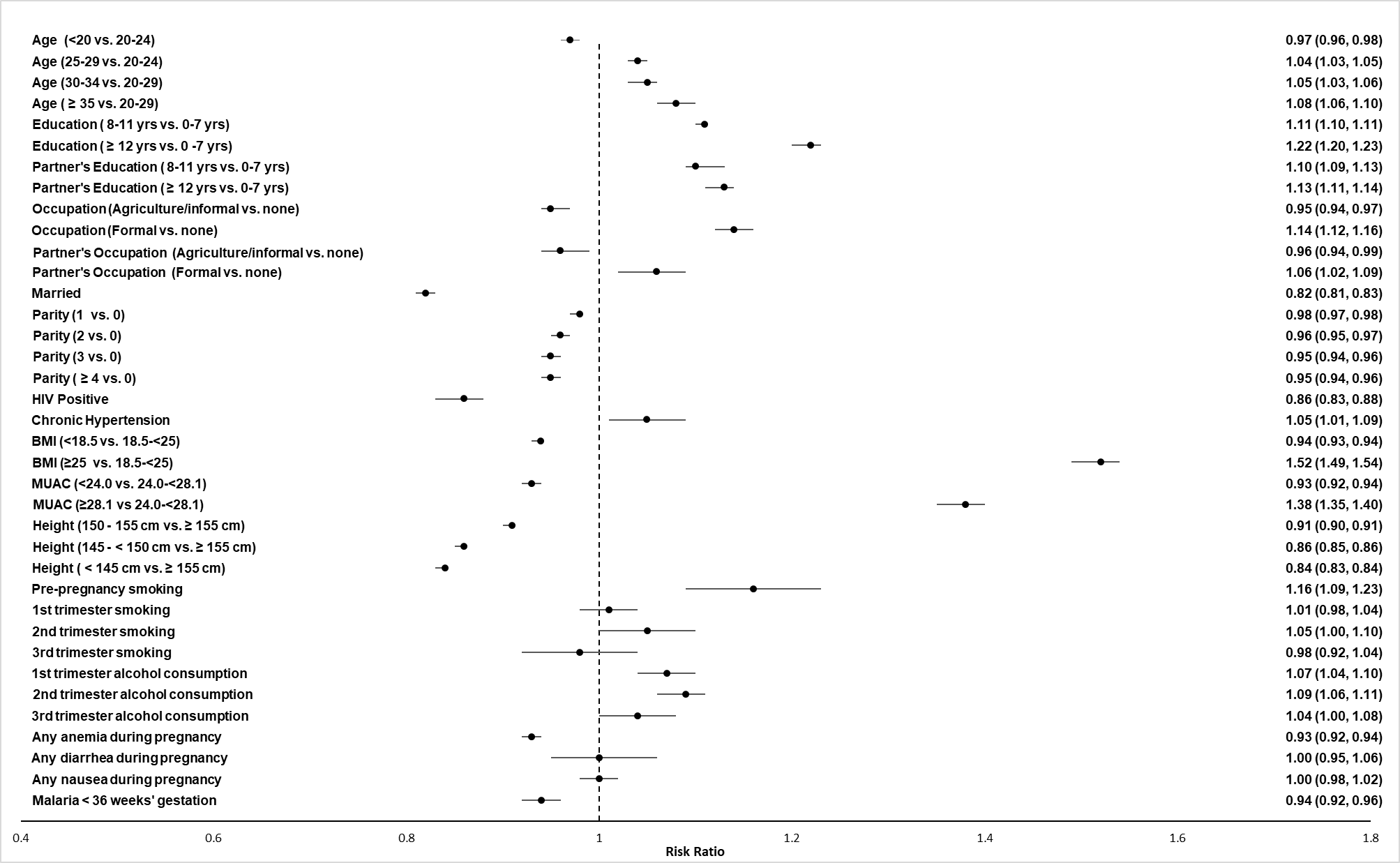


GWG=Gestational weight gain, BMI=body mass index, MUAC=mid-upper arm circumference, HIV=Human immunodeficiency virus, cm=centimeter
